# Effect of anthocyanins on gut health markers, *Firmicutes*-*Bacteroidetes* ratio and short-chain fatty acids: a systematic review via meta-analysis

**DOI:** 10.1038/s41598-023-28764-0

**Published:** 2023-01-31

**Authors:** Payal Kapoor, Apoorv Tiwari, Saloni Sharma, Vandita Tiwari, Bhawna Sheoran, Usman Ali, Monika Garg

**Affiliations:** 1 National Agri-Food Biotechnology Institute, Mohali, Punjab 140308 India; 2 Department of Biochemistry, Panjab University, Chandigarh, India; 3 Regional Centre of Biotechnology, Faridabad, Haryana India

**Keywords:** Biotechnology, Health care

## Abstract

Researchers discovered that diets rich in anthocyanin-rich fruits and vegetables significantly impacted gut flora. To conclude, large-scale randomized controlled clinical trials are challenging to conduct; therefore, merging data from multiple small studies may aid. A systematic review collects and analyses all research on a particular subject and design. This comprehensive review and meta-analysis examined the influence of dietary anthocyanins on *Firmicutes/Bacteroide* (Fir/Bac) and short-chain fatty acids (SCFAs) content. The current meta-analysis followed the guidelines of PRISMA—the preferred reporting items for systematic reviews and meta-analyses. Diets high in anthocyanins substantially reduced the Fir/Bac ratio in the assessed trials. Among three SCFAs, the highest impact was observed on acetic acid, followed by propionic acid, and then butanoic acid. The meta-analysis results also obtained sufficient heterogeneity, as indicated by I^2^ values. There is strong evidence that anthocyanin supplementation improves rodent gut health biomarkers (Fir/Bac and SCFAs), reducing obesity-induced gut dysbiosis, as revealed in this systematic review/meta-analysis. Anthocyanin intervention duration and dosage significantly influenced the Fir/Bac ratio and SCFA. Anthocyanin-rich diets were more effective when consumed over an extended period and at a high dosage.

## Introduction

Polyphenols are phytochemicals in various foods, including fruits and vegetables, tea, coffee, chocolate, legumes, and cereals. The primary function of polyphenols is to act as antioxidants and quench free radicals^1^. Dietary polyphenols are gaining scientific attention due to their health benefits. Several clinical studies have found that polyphenols can help protect against cancer, cardiovascular disease, aging, and neurodegenerative diseases^2–4^. Anthocyanins are among the most potent polyphenols due to their chemical structure, i.e., the abundance of hydroxyl groups. Anthocyanins are pigments that give plants vibrant color [purple, blue, and red] and have antioxidant properties^5^. Several studies have discovered that they can aid in preventing obesity, diabetes, and metabolic disorders by improving gut health and microbiota^6–9^. An individual's gut microbiota is complex, containing thousands of different bacterium species and trillions of microbes^10^. The gut microbiota varies with the dietary pattern, co-evolves with the host, and has a symbiotic relationship^11^. The majority of the gut microbiota is considered non-pathogenic. Scientific data from numerous experimental and clinical studies have established the health benefits of healthy gut microbiota^12,13^. However, specific stimuli may change their composition over time, leading to a condition known as dysbiosis, favoring pathogenic microbes and negatively affecting the gastrointestinal tract, immune system, central nervous system, and metabolic machinery. These conditions lead to irritable bowel syndrome [IBS], inflammatory bowel diseases, allergies, Alzheimer's and Parkinson's, and type 1 diabetes, among others^14–16^. As a result, it is vital to identify potentially beneficial bacteria that could aid in developing treatments that protect people from the adverse effects of gut dysbiosis. The ratio of two major microbial phyla, *Firmicutes/Bacteroidetes* [Fir/Bac], and the level of short-chain fatty acids [SCFAs] are frequently regarded as vital indicators of an individual's gut health status. Obese people, for example, have a higher Fir/Bac ratio than lean people^17,18^. The healthy gut microbiota metabolizes indigestible dietary components to SCFAs^17,18^. SCFAs such as acetic acid, propionic acid, and butyric acid, acidify the intestinal pH and inhibit pathogenic bacteria such as Enterobacteriaceae from propagating^19^. Propionate is essential for gluconeogenesis, whereas acetate is important for lipogenesis^20^. Butyrate gives energy to colon cells, keeps the structure of the biological membrane stable, and encourages the growth of colonocytes^21^.

Anthocyanins can pass through the gastrointestinal mucosa in their natural state. Hydrolytic enzymes in the small intestine absorb them as phenolic aglycone, especially in the jejunum. The anthocyanins do not pass through the colon. The colonic microbiota metabolizes unabsorbed anthocyanins into simpler metabolites. These metabolites have been shown to influence the proliferation of beneficial bacteria such as bifidobacterium^22^, Fir/Bac ratio, and SCFA production^22–27^.

The health effects of a molecule are usually concluded based on large-scale randomized controlled clinical trials, which are notoriously difficult to conduct. However, combining data from several small studies can aid in the conclusion. A systematic review compiles all possible studies on a specific topic and design, then reviews and analyses their findings. During the systematic review process, the quality of the studies is evaluated, and a statistical meta-analysis of the study results is performed based on their quality. A meta-analysis is a legal, objective, and scientific method of analyzing and combining different results. Previous meta-analysis studies looked into the effects of anthocyanin-rich diets on cardiovascular health and oxidative stress^28,29^. Nonetheless, the effect of anthocyanins on the gut microbiota, particularly the Fir/Bac ratio and SCFA concentration, has yet to be thoroughly reviewed. Thus, this systematic review and meta-analysis aim to conclude the effect of dietary anthocyanins on the Fir/Bac ratio and SCFA content.

## Materials and methods

The current meta-analysis study followed the Preferred Reporting Items for Systematic Reviews and Meta-analyses guidelines (PRISMA)^30^.

### Literature search

Scientific databases, including Scopus, PubMed, Science Direct, Web of Science, and MEDLINE, were searched up to 2022. The search terms or keywords included gut microbiota and anthocyanins; Gut microbiota, anthocyanins, and animal study; Anthocyanin-rich fruits and gut microbiota; Anthocyanin-rich vegetables and gut microbiota; and Anthocyanin-rich vegetables and gut microbiota; Anthocyanins, *Firmicutes, Bacteroidetes*; Anthocyanins and in vivo gut microbiota; Anthocyanins, gut microbiota, short-chain fatty acids. We have also formulated the search using the PICO framework for evidence-based practice (STable 1). Because the PICO framework is used in systematic reviews to create literature search tactics that are both thorough and objective.

### Criteria for study selection, inclusion, and exclusion

Revised ARRIVE guidelines (Animal Research: Reporting In Vivo Experiments)^31^ designed to help researchers and publishers identify the minimum necessary information for scientific reporting of in vivo experiments, such as inclusion and exclusion criteria, were followed. The titles of the collected articles were examined first, followed by the selection of abstracts and confirmation of manuscript content. The following inclusion criteria were specified during the study selection process. (a) Clearly stated study design; (b) Animal studies [mice and rats]; (c) A minimum of three subjects (d) Anthocyanin supplementation in purified, extract, whole fruit or juice form; (e) Control mentioned (f) Intervention duration > one week; (g) Data for *Firmicutes* to *Bacteroidetes* ratio, acetic acid, propionic acid, and butyric acid. English-language studies were preferred.

Two authors reviewed the studies from the initial search to identify those relevant to the study. This allowed us to exclude studies that did not address the purpose of the study or the previously stated requirements. A kappa analysis can be performed to check for consistency in interpreting the selection criteria between the two reviewers. Using Cohen's kappa coefficient^32^, we could determine if there was substantial agreement between reviewers in each study. The formula for Cohen's kappa is calculated as follows:

$$k = \left( {po - pe} \right) \div \left( {1 - pe} \right)$$

where po: Relative observed agreement among raters, pe: Hypothetical probability of chance agreement.

### Data extraction

The following information was extracted from each article: author, year of publication, subjects’ clinical characteristics, sample size, study duration, source of anthocyanins, daily dosage, means, and standard deviations (SD) of the Fir-Bac ratio and SCFAs^33^. If the trial included standard errors (SE), the SE was converted to SD by multiplying the SE by the square root of the sample size. The unit of SCFAs (acetic acid, propionic acid, butyric acid) was µmol/gm. Different anthocyanin interventions given to animals were formalized to mg/kg body weight in all studies. For dose conversion in mg/kg of body weight, average weight and diet considered for mice were 22 g and 2.5 g, and for rats, 200 g and 11 g, respectively (STable 2, 3).

### Statistical analysis

The standardized mean difference (SMD) was calculated using Hedges' adjusted g. The weighted mean differences (MD) for net change and 95 percent confidence intervals (CI) were used to estimate the effect of anthocyanins on the Fir/Bac ratio and SCFA concentration^34^. The Forest plots were created to display the SMDs and CIs, which represent each study's observed effect, confidence interval, and weight^35^. Statistical tests for heterogeneity I^2^, Chi^2^, and Tau^2^ were used to assess the consistency of the study's results. The I^2^ values of 25%, 50%, and 75% were considered low, moderate, and high heterogeneity, respectively. The treatment groups receiving low and high doses of anthocyanin were chosen for dose comparison through meta-analysis. In studies with more than two anthocyanin treatments or anthocyanin-rich food interventions, each treatment group was compared to the control group^36^. The influence of anthocyanins on the Fir/Bac ratio and SCFAs was calculated using a random-effects analysis model. Subgroup analyses were performed to identify potential contributory variables^37^. In the Fir/Bac analysis, studies were classified into subgroups based on the duration (less than ten weeks vs. equal to and more than ten weeks), dose (higher and lower doses as per respective studies), an animal model type (High fat diet-induced obesity, diabetes, and other diseases). For SCFA analysis, studies were classified similarly into subgroups based on the duration (less than four weeks vs. equal to and more than 4 weeks), dose, and animal model type. The RevMan 5.4 package^38^ and the R script (meta-package) were used for all statistical studies^39^.

### Data evaluation/testing

GRADEprofiler (GRADEpro) tool was used for the analysis of data quality. For systematic reviews and recommendations in healthcare, the Grading of Recommendations Assessment, Development, and Evaluation (GRADE) provides a transparent and structured approach for creating and presenting evidence summaries, including the quality of that evidence^40^. Using GRADE, we classified the quality of our meta-analysis results into two categories: higher and lower.

### Publication bias test

Begg's and Egger's regression asymmetry tests were used for estimating publication bias in various forms, such as time-lag bias (caused by delayed publication), duplicate or multiple publications, outcome reporting bias (only reporting good results), and language bias. Egger test used linear regression to test asymmetry with numbers by examining the relationship between the standardized effect estimates and the standard error^41^. Begg's test assessed the significance of the correlation between the ranks of the effect estimates and the ranks of their variances^42^. The minor corrections were implemented using the Trim-Fill correction method in all the studies, including Fir/Bac and SCFA.

### Bibliometric analysis

A bibliometric study was conducted by selecting the articles published (indexed in the Pubmed database) till March 2022 using the search terms *anthocyanin*, *gut microbiota*, and/or *SCFA* to know the research output progress on anthocyanin. The publications were downloaded in the Medline file format. After selection, visualization of the thematic contiguity of the articles was carried out using the Vos Viewer tool, which enabled the network charts. The network visualization consists of multiple-colored bubbles. Each bubble with a single color belongs to a ‘cluster.’ Bubbles that are distantly located from others have a weak relationship among them. Moreover, the number of links between two bubbles depicted the level of interaction between the items under consideration.

## Result

### Literature search

**Figure 1:**
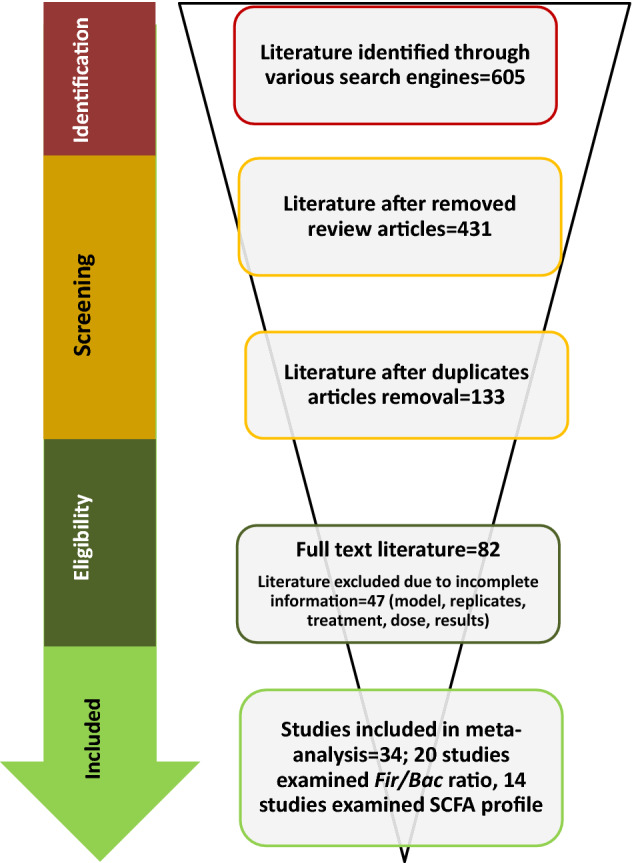
Meta-Analysis flow chart as per PRISM guidelines.

Detailed information on the search strategy and the process followed for the meta-analysis has been displayed in the PRISMA flowchart (Fig. 1). We identified 605 articles using various search engines through a literature survey. Of these articles, 173 were review articles, and 432 were research articles. From the total research articles selected for the study, 298 were duplicates and therefore removed. Afterward, article abstracts and the full text of 133 articles were read thoroughly and checked to determine whether they met the eligibility criteria. Those studies that did not meet the eligibility criteria (“Criteria for study selection, inclusion, and exclusion” section) were also removed. Thirty-four studies met the eligibility criteria. Out of it, 20 and 14 articles examining the effect of anthocyanins on the Fir/Bac ratio and the concentration of SCFAs, respectively, were included for meta-analysis. Figure 1 shows the flow and data extraction of the current study.

The first criteria we applied for screening total studies was 605; in that case % of the agreement was 96.89%, Cohen’s k: 0.91. The second screening was performed on 432 selected articles; in that case % of the agreement was 97.85%, and Cohen’s k was 0.95. In the third screening, the % of agreement: was 96.33%, Cohen’s k: 0.91, and in the fourth screening total of 34 studies were selected with % of agreement: 97.47% and Cohen’s k: 0.93. To pass the test, you should aim for a kappa score of 0.5 or higher. Near-perfect agreement in the selection and filtering of studies was observed, with values over 0.9; differences were discussed and resolved by consensus.

### Study characteristics

The characteristics of the studies examining the effects of anthocyanins on the Fir/Bac ratio are mentioned in Table 1. Of the total studies, 14 investigated the impact of the intervention of anthocyanins from various berry fruits. The remaining studies included interventions from other sources like cereals and pulses (Table 1). Seventeen studies were conducted on males, one was conducted on female mice models, and two were performed on male rats.

**Table 1:** Characteristics of the studies used to investigate the effect of anthocyanins on the Fir/Bac ratio.

S.no.	Animal	Age [weeks]	Model type	Source	Intervention compound	Dose	Intervention duration [weeks]	References	Doi
1	Male C57BL/6 J mice	7–9	High fat diet	Roselle	Flower water extract [**Phenolic extract**]	1 mg/kg body weight [L]	6	^43^	https://doi.org/10.1016/j.foodres.2019.108722
						10, mg/kg body weight [M]			
						25 mg/kg body weight [H]			
2	Male C57BL/6Cnc mice	4	Highfat, highfructose diet	Grape	Fruit ethanol extract [**Phenolic extract**]	405 mg/kg body weight	12	^44^	https://doi.org/10.1002/mnfr.202000149
3	Male C57BL/6 J mice	4	High fat diet	Russian box thorn	Fruit based commercial powder** [Anthocyanins]**	50 mg/kg body weight [L]	12	^24^	https://doi.org/10.1002/mnfr.202000745
						100 mg/kg body weight [M]			
						200 mg/kg body weight [H]			
4	Male Wistar rats	NA	High fat diet	Blackberry	Fruit acidified ethanol extract [**Anthocyanins**]	25 mg/kg body weight	17	^45^	https://doi.org/10.1038/s41598-018-29744-5
5	Male C57BL/6 mice	6	High fat diet	Blueberry	Fruit fermented juice	4 ml/kg	17	^46^	https://doi.org/10.1039/D0FO00334D
6	Male C57BL/6JbomTac mice	6	High fat diet	Lingonberries	Fruit freeze dried	22725 mg/kg body weight	11	^47^	https://doi.org/10.3402/fnr.v60.29993
					**Phenolic compound**	138.6 mg/kg body weight			
7	Male C57BL/6 J mice	6	High fat, high sucrose diet	Blueberries	Fruit hydro-ethanolic extract [**Fraction rich in anthocyanin and phenolic acids**]	32 mg/kg body weight [L]	8	^48^	https://doi.org/10.1038/s41598-020-58863-1
					**Fraction rich in oligomeric PACs, phenolic acids and flavonols**	53 mg/kg body weight [H]			
					**Fraction rich in polymeric PACs**	37 mg/kg body weight [M]			
8	Male C57BL/6 J	6	High fat diet	Tea [Purple-leaf]	Leaves dried	1137 mg/kg body weight [L]	10	^49^	https://doi.org/10.1186/s12906-020-03171-4
						3409 mg/kg body weight[H]			
					**Phenolic compounds**	12.6 [L], 37.8[H] mg/kg body weight			
					**Anthocyanins**	1.8 [L], 5.4 [M]mg/kg body weight			
9	Male C57BL/6N mice	5	Western diet	Bilberry	Fruitsdried	2273 mg/kg body weight	18	^50^	https://doi.org/10.3390/nu12113252
					**Anthocyanins**	5.7 mg/kg body weight			
10	Male C57BL6/J mice	6	Cholesterol diet	Black rice	Fruit based commercial extract [**Anthocyanins**]	13.6 mg/kgbody weight [L]	12	^51^	https://doi.org/10.1002/mnfr.201900876
						27.3 mg/kg body weight [M]			
						54.4 mg/kg body weight [H]			
11	Male C57BL/6 mice	5	Dextran sodium sulfate induced colitis	Russian box thorn	Fruithydro-acidic ethanolic extract [**Anthocyanins**]	200 mg/kg body weight	2.2	^23^	https://doi.org/10.1016/j.freeradbiomed.2019.04.005
12	Male C57BL/6 J mice	4	High fat diet	Black currant	Fruithydro-acidic ethanolic extract [**Anthocyanins**]	150 mg/kg body weight	14	^52^	https://doi.org/10.1002/mnfr.202001090
13	Female C57BL/6 mice	8	Colon cancer	Bilberry	Fruit based commercial powder [**Anthocyanins**]	25 mg/kg body weight	2	^53^	https://doi.org/10.3390/microorganisms8020175
14	Male diabetic Zucker rats	3	High fat diet	Bilberries and purple potato	Bilberry fruit [a,b] and potato tuber [c,d] commercial extract [50:50 ratio] [**Anthocyanins**]	25 mg/kg body weight [L:a,c]	8	^54^	https://doi.org/10.1016/j.foodres.2022.110978
						50 mg/kg body weight [H: b,d]			
15	Male C57BL/6 J mice	5	High fat diet	Russian box thorn	Fruithydro-acidic ethanolic extract [**Anthocyanins**]	100 mg/kg body weight	11	^55^	https://doi.org/10.3390/foods11010098
16	Male C57BL/6 J mice	NA	High fat diet	Undefined	Commercial powder [**Anthocyanins**]	40 mg/kg body weight	14	^56^	https://doi.org/10.1016/j.redox.2019.101269
17	Male C57BL/6 J mice	5	High fat diet	Blueberry [a,b] and cranberry [c,d]	Commercial powder[50:50 ratio] [**Anthocyanins**]	1137 mg/kg body weight [L:a,c]	24	^57^	https://doi.org/10.1007/s00394-020-02446-3
						2273 mg/kg body weight [H:b,d]			
18	Male C57BL/6 mice	7	High fat diet	Jamun [black plum]	Fruit pulp hydro-ethanol/acetone extract [**Phenolic extract**]	100 mg/kg body weight	8	^58^	https://doi.org/10.1002/mnfr.201801307
19	Male C57BL/6 J mice	6	High fat, cholesterol diet	Purple sweet potato	Tuber acidified methanolic extract [**Anthocyanins**]	340 mg/kg body weight [L]	12	^59^	https://doi.org/10.1111/1750-3841.16130
						681.8 mg/kg body weight [M]			
						1022.6 mg/kg body weight [H]			
20	Male Swiss-albino mice	6–8	Normal	Purple and black wheat	Seed powder [a,c] and cooked chapatti powder [b,d]	96.6 g/kg body weight [L: a, c]	11	^60^	https://doi.org/10.1016/j.jcs.2022.103433
						104.5 g/kg body weight [H: b,d]			
					**Anthocyanins**	Purple Flour = 5.7 [a] Black flour = 15.4 [b] Purple Chapatti = 2.0 [c] Black chapatti = 10.3 [d] [mg/kg body weight]			

^#^For understanding the effect of dose, different interventions given to animals were uniformalised to mg/kg body weight. For dose conversion in mg/kg of body weight, the average weight and diet considered for mice were 22 g and 2.5 g, and for rats were 200 g and 11 g, respectively.

Study characteristics examining the effect of anthocyanins on SCFA profile (acetic, propionic, and butyric acid) were mentioned in Table 2. Ten studies were conducted on male mice, one on female mice, and three on male rats. Twelve studies that looked at the effect of the anthocyanins-rich diet intervention on the concentration of SCFAs in the cecal matter of the different subjects looked at the effect of berries, and one study each looked at the effect of black rice and purple sweet potatoes.

**Table 2:** Characteristics of the studies used to investigate the effect of anthocyanins on the short chain fatty acids [SCFA’s].

	Animal	Age [weeks]	Model type	Source	Intervention compound	Dose	Duration [weeks]	References	Doi
1	Male C57BL/6 J mice	8	High fat, high sucrose diet	Blueberry	Fruits dried	727.2 mg/kg body weight	8	^61^	https://doi.org/10.1152/ajpendo.00560.2019
					**Anthocyanins** [Size based fractionation]	77.3 mg/kg body weight			
					P**roanthocyanins** [Size based fractionation]	4.6 mg/kg body weight			
2	Kunming mice	NA	Diphenoxylate induced constipation	Mulberry	Fruit dried	142 mg/kg body weight [L]	2	^62^	https://doi.org/10.1039/C9FO00132H
						284.1 mg/kg body weight [M]			
						568.1 mg/kg body weight [H]			
					**Anthocyanins**	0.7 [L], 1.4 [M], and 6.82 [H] mg/kg body weight			
3	Male C57BL/6 mice	6	Western diet	Montmorency tart cherry	Fruit dried	5681.3 mg/kg of body weight [L]	12	^63^	https://doi.org/10.1016/j.nutres.2021.10.003
						11,362 mg/kg of body weight [H]			
					**Anthocyanins**	1.58 [L], 3.16 [H] mg/kg of body weight			
4	Male C57BL/6 mice	4–5	High fat diet	Raspberry	Fruit pulp hydro-acidified methanolic extract [**Anthocyanins**]	22.8 mg/kg body weight	12	^64^	https://doi.org/10.1039/C7FO02061A
5	Male db/db mice with C57BL/6 J background	6	Diabetic Mice	Wild raspberry	Fruit hydro-acidified methanolic extract [**Anthocyanins**]	150 mg/kg of body weight	8	^65^	http://doi.org/10.1021/acs.jafc.9b03338
6	Male C57BL6/J mice	6	High fat, cholesterol diet	Black rice	Fruit based commercial extract [**Anthocyanins**]	13.6 mg/kg body weight [L]	12	^51^	http://doi.org/10.1002/mnfr.201900876
						27.3 mg/kg body weight [M]			
						54.6 mg/kg body weight [H]			
7	Male Wistar rats	3	Normal	Brazilian berry	Fruit peel water extract [**Phenolic extract**]	Undefined	7	^66^	https://doi.org/10.1111/jfbc.12705
8	Male Wistar rats	3	Colitis model	Brazilian berry	Fruit peel water extract [**Phenolic extract**]	141 and 151 mg/kg of body weight mg/kg of body weight [Short term treatment-L]	7	^67^	https://doi.org/10.3390/nu11112776
						215 and 208 mg/kg of body weight mg/kg of body weight [Long term treatment-H]			
9	Male Wistar rats	NA	High fat diet	Blueberry	Fruit Dried	113.63 mg/kg body weight	8	^68^	https://doi.org/10.1093/jn/nxx027
					**Phenolic + anthocyanin extract**	6.79 mg/kg body weight			
10	Male C57BL/6 mice	5	Normal	Russian box thorn	Fruit hydro-ethanolic extract [**Phenolic extract**]	200 mg/kg of body weight	12	^69^	https://doi.org/10.1016/j.foodres.2019.108952
11	Male C57BL/6 J mice	4	High fat diet	Russian box thorn	Fruit **Anthocyanins** extract	50 mg/kg body weight [L]	12	^24^	https://doi.org/10.1002/mnfr.202000745
						100 mg/kg body weight [M]			
						200 mg/kg body weight [H]			
12	Male C57BL/6 mice	5	DSS-colitis model	Russian box thorn	Fruit hydro- ethanolic extract [**Phenolic extract**]	200 mg/kg body weight [ACN:a, P3G:b]	2.4	^23^	https://doi.org/10.1016/j.freeradbiomed.2019.04.005
13	Female C57BL/6 mice	8	Colon Cancer	Bilberry	Fruit based commercial powder [**Anthocyanins**]	25 mg/kg body weight	2	^53^	https://doi.org/10.3390/microorganisms8020175
14	Male C57BL/6 J mice	6	High fat, cholesterol diet	Sweet potato	Tuber based commercial powder [**Anthocyanins**]	340.8 mg/kg body weight [L]	12	^59^	https://doi.org/10.1111/1750-3841.16130
						681.8 mg/kg body weight [M]			
						1022.6 mg/kg body weight [H]			

^#^For understanding the effect of dose, different interventions given to animals were uniformalised to mg/kg body weight. For dose conversion in mg/kg of body weight, the average weight and diet considered for mice were 22 g and 2.5 g, and for rats were 200 g and 11 g, respectively.

### Effect of anthocyanins on the Fir/Bac

**Figure 2:**
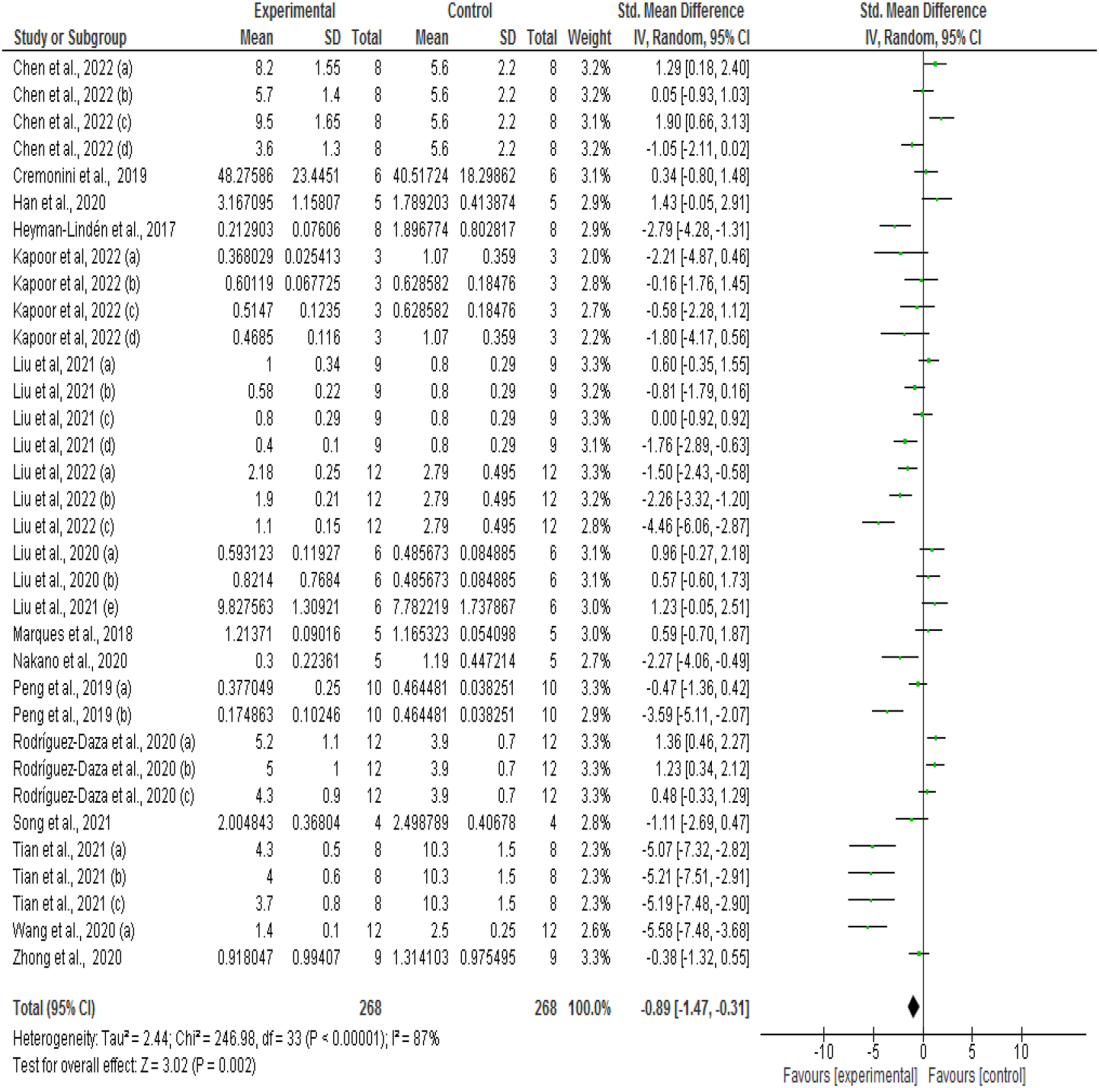
Forest plot of studies investigating the effect of anthocyanin supplementation on the *Firmicutes* to *Bacteroidetes* ratio [Fir/Bac]. Pooled effect estimates [diamonds] for Fir/Bac are shown after removing highly influencing studies. Values are standardized mean differences with 95% CIs determined with the use of random-effects models. Heterogeneity was quantified by I^2^, inverse variance and standardised mean difference [SMD].

The anthocyanin-rich diet intervention significantly decreased the Fir/Bac ratio (SMD: − 1.80; 95% CI − 2.48, − 1.12; I^2^ = 90%; *P* < 0.00001) in all the studies under consideration (Table 3 and Supplemental Fig. 1). The meta-analysis result also obtained sufficient heterogeneity, as indicated by I^2^ values. Regarding the contribution of individual studies, some showed non-significant results, and others had a relatively higher influence on overall value than others. However, a comprehensive Fir/Bac ratio study produced statistically significant positive results. Four studies, including Diez-Echave et al.^43^, Wang et al.^51^ (Medium and High doses); Lin et al.^49^; and Xu et al.^58^, had more comprehensive cumulative interval ranges, which means there was more uncertainty about the usefulness of these interventions. When the studies mentioned above were deleted before analysis, the Fir/Bac ratio significantly reduced, but overall values changed (SMD: − 0.89; 95% CI − 1.47, − 0.31; I^2^ = 87%; *P* 0.002) (Fig. 2).

**Table 3:** All-inclusive, high-influencer subtracted and sub-group [intervention duration, dose, and animal model type] analysis to understand the effects of anthocyanins on the Fir/Bac ratio.

Study/ Subgroup type	SMD	95% CI	*P* value	I^2^[%]
Whole study	− 1.80	− 2.48, − 1.12	0.00001	90
After removing Highly influencing studies	− 0.89	− 1.47, − 0.31	0.002	87
Study duration				
< 10 weeks	− 1.49	− 2.64, − 0.33	0.01	92
After removing Highly influencing studies	0.30	− 0.43, 1.03	0.42	82
≥ 10 weeks	− 2.16	− 2.96, − 1.36	0.0001	87
After removing Highly influencing studies	− 1.81	− 2.56, − 1.05	0.00001	86
Dose of anthocyanin				
Lower Dose	− 1.12	− 2.47,0.23	0.11	91
After removing Highly influencing studies	− 0.60	− 1.74,0.54	0.30	89
Higher Dose	− 2.33	− 3.69, − 0.98	0.0007	91
After removing Highly influencing studies	− 1.79	− 2.95, − 0.64	0.002	89
Study model type				
High fat diet model	− 2.31	− 3.31, − 1.31	0.0001	92
After removing Highly influencing studies	− 0.94	− 1.78, − 0.11	0.03	90
Other models [Western diet, tumour, colitis]	− 2.25	− 3.64, − 0.86	0.002	89
After removing Highly influencing studies	− 1.72	− 2.97, − 0.46	0.007	86

Pooled effect sizes and 95% CI were determined using random effects model.

*SMD* Standardised mean difference, *I*^2^ Heterogeneity.

Similar meta-analyses, i.e., without highly influencing studies with wider cumulative interval ranges, were performed in each sub-group. Forest plots in Supplemental Figs. 2 and 3 show subgroup analyses investigating the effect of anthocyanin-rich diet intervention on the Fir/Bac ratio based on the duration, anthocyanin dose, and study model type. The meta-analyses results indicated that intervention duration of the more extended period, i.e., ≥ 10 weeks, significantly reduced the Fir/Bac ratio (SMD = − 1.81; 95% CI − 2. 56, − 1.05; I^2^ = 86%; *P* < 0.0001), whereas intervention study for a shorter period, i.e., less than 10 weeks had no effect (SMD = 0.30; 95% CI − 0.43, 1.03; I^2^ = 82%; *P* < 0.42). Similarly, the effect of higher intervention doses was more pronounced (SMD = − 1.79; 95% CI − 2.95, − 0.64; I^2^ = 89%; *P* < 0.002) as compared to lower doses (SMD = − 0.60; 95% CI − 1.74, − 0.54; I^2^ = 89%; *P* < 0.30). There was no effect of the type of study model. Anthocyanin-rich intervention remarkably reduced the Fir/Bac ratio irrespective of the study model type. It reduced in high fat/cholesterol diet-induced obese subjects (SMD = − 0.94; 95% CI − 1.78, − 0.11; I^2^ = 90%; *P* < 0.03) as well as in other model studies including western diet, dextran sodium sulphate [DSS]-induced colitis, and tumor (SMD = − 1.72; 95% CI − 2.97, − 0.46; I^2^ = 86%; *P* < 0.007) (Table 3, Supplemental Fig. 2 and 3). Finalized data quality was evaluated by Grade Tool (Supplemental Fig. 4) and showed moderate heterogeneity that is serious inconsistency.

**Figure 3:**
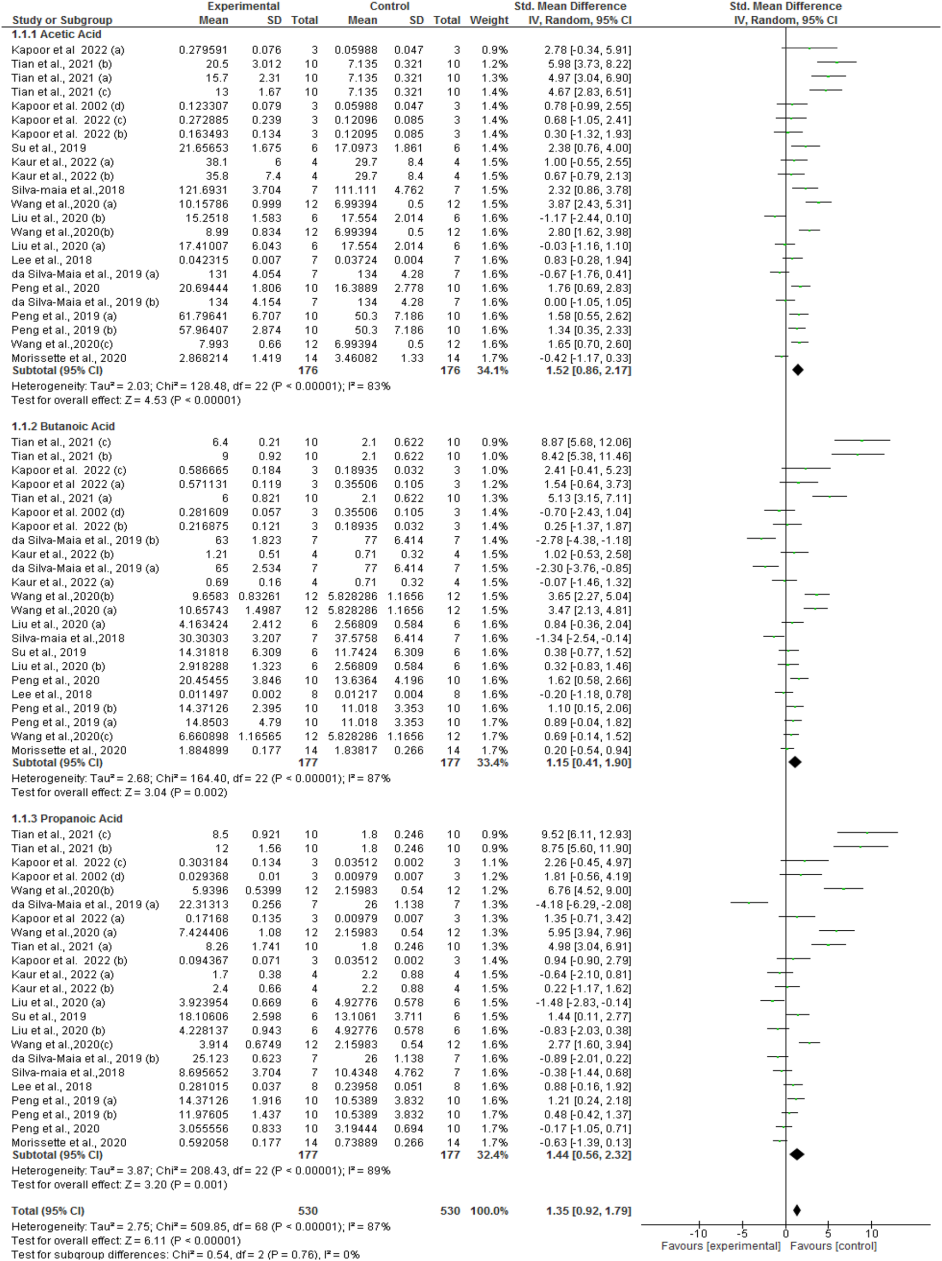
Forest plot of studies investigating the effect of anthocyanin supplementation on the SCFA profile, sub-grouped by short chain fatty acid type. Pooled effect estimates are shown by diamonds after removing highly influencing studies. Values are standardized mean differences with 95% CIs determined with the use of random-effects models. Heterogeneity was quantified by I^2^, inverse variance and standardised mean difference [SMD].

### Effect of anthocyanins on the short chain fatty acids (SCFA’s) production

The meta-analyses showed a significant effect of the anthocyanin-rich diet intervention on acetic, propionic, and butanoic acid concentration (Table 4, Fig. 3, and Supplemental Fig. 5). Of the three SCFAs, the highest impact was observed on the acetic acid (SMD:1.52; 95% CI 0.86,2.17 µmole/gm; I^2^ = 83%; *P* < 0.00001]; followed by propionic acid (SMD:1.44; 95% CI 0.56, 2.32 µmole/gm; I^2^ = 89%; *P* = 0.001) and then butanoic acid (SMD: 1.15; 95% CI 0.41, 1.90 µmole/gm; I^2^: 87%; *P* value = 0.002). High heterogeneity was obtained, as indicated by I^2^ values.

**Table 4:** Pooled effects of anthocyanins from various sources on short chain fatty acid profile including all-inclusive and high-influencer studies.

Parameters	Acetic acid	After removing Highly influencing studies	Butanoic acid	After removing Highly influencing studies	Propionic acid	After removing Highly influencing studies
SMD	2.69	1.52	1.60	1.15	2.33	1.44
95% CI [µmole/gm]	1.88, 3.50	0.86, 2.17	0.82, 2.39	0.41, 1.90	1.45, 3.22	0.56, 2.32
*P* value	0.00001	0.00001	0.0001	0.002	0.00001	0.001
I^2^[%]	90	83	90	87	92	89

Pooled effect sizes and 95% CI were determined using Random effects model.

*SMD* Standardised mean difference, *I*^2^ Heterogeneity.

Each short-chain fatty acid was sub-grouped based on intervention duration, anthocyanin dose, and model type. We found a considerable increase in acetic acid concentration when the intervention was continued for ≥ 4 weeks (SMD: 1.78; 95% CI 1.01, 2.54 µmole/gm; I^2^: 84%; *P* < 0.00001) as compared to the nonsignificant effect of intervention followed for less than 4 weeks (SMD:0.47; 95% CI − 0.73, 1.68µmole/gm; I^2^: 79%; *P* < 0.44). (Table 5 and Supplemental Figs. 6 and 7). The intervention of anthocyanin at a higher dose imparted a remarkable impact on acetic acid (SMD: 2.58; 95% CI 0.92, 4.24 µmole/gm, I^2^: 76%; *P* = 0.002) compared to a lower dose (SMD: 1.65; 95% CI 0.33, 2.98 µmole/gm, I^2^: 74%; *P* = 0.01). The anthocyanins exerted a significant effect on acetic acid concentration in high fat/cholesterol diet model type (SMD: 2.89; 95% CI 1.40, 4.37µmole/gm, I^2^: 91%; *P* = 0.0.0001) as compared to another model type (SMD: 0.81; 95% CI 0.24, 1.37µmole/gm, I^2^: 62%; *P* = 0.005) (Table 5; Supplemental Fig. 6 and 7).

**Table 5:** All-inclusive, high-influencer subtracted and sub-group [intervention duration, dose, and animal model type] analysis to understand the effects of anthocyanins on the acetic acid.

Study/ Subgroup type	SMD	95% CI	*P* value	I^2^[%]
Study duration				
< 4 weeks	2.97	1.01, 4.93	0.003	92
After removing Highly influencing studies	0.47	− 0.73, 1.68	0.44	79
≥ 4 weeks	2.63	1.73, 3.54	0.00001	89
After removing Highly influencing studies	1.78	1.01, 2.54	0.00001	84
Dose of anthocyanin				
Lower Dose	2.65	1.12, 4.19	0.0007	86
After removing Highly influencing studies	1.65	0.33, 2.98	0.01	74
Higher Dose	3.81	1.41, 6.20	0.002	92
After removing Highly influencing studies	2.58	0.92, 4.24	0.002	76
Study model type				
High fat diet model	4.36	2.80, 5.92	0.00001	93
After removing Highly influencing studies	2.89	1.40, 4.37	0.0001	91
Other models [Western diet, tumour, colitis]	1.63	0.80, 2.47	0.0001	83
After removing Highly influencing studies	0.81	0.24, 1.37	0.005	62

Pooled effect sizes and 95% CI were determined using Random effects model.

*SMD* Standardised mean difference, *I*^2^ Heterogeneity.

We have found a higher rise in the butanoic acid concentration for a more extended period of study duration (SMD: 1.30; 95% CI 0.36, 2.25µmole/gm; I^2^: 89%; *P* < 0.007) as compared to a shorter period, i.e., < 4 weeks (SMD:0.82; 95% CI 0.30, 1.34 µmole/gm; I^2^: 0%; *P* < 0.002) (Table 6; Supplemental Fig. 8 and 9). Also, the butanoic acid concentration was significantly higher in the subjects taking a higher dose of anthocyanins (SMD: 3.32; 95% CI 1.53, 5.11 µmole/gm, I^2^: 79%; *P* = 0.0003) compared to the subjects administered lower dose (SMD: 0.97; 95% CI − 0.57, 2.50 µmole/gm, I^2^: 83%; *P* = 0.22). The study subjects showed a remarkable rise in butanoic acid concentration in the high-fat diet-induced obesity model (SMD: 3.34; 95% CI 1.65, 5.03µmole/gm, I^2^: 93%; *P* = 0.0001) compared to other model types (SMD: 0.17; 95% CI − 0.49, 0.83µmole/gm, I^2^: 72%; *P* = 0.61).

**Table 6:** All-inclusive, high-influencer subtracted and sub-group [intervention duration, dose, and animal model type] analysis to understand the effects of anthocyanins on the Butanoic acid.

Study/ Subgroup type	SMD	95% CI	*P* value	I^2^[%]
Study duration				
< 4 weeks	0.93	− 0.80, 2.56	0.29	91
After removing Highly influencing studies	0.82	0.30, 1.34	0.002	0
≥ 4 weeks	1.81	0.90, 2.73	0.0001	90
After removing Highly influencing studies	1.30	0.36, 2.25	0.007	89
Dose of anthocyanin				
Lower Dose	0.31	− 1.27, 1.89	0.70	90
After removing Highly influencing studies	0.97	− 0.57, 2.50	0.22	83
Higher Dose	3.08	1.50, 4.66	0.0001	86
After removing Highly influencing studies	3.32	1.53, 5.11	0.0003	79
Study model type				
High fat diet model	3.64	2.31, 4.97	0.00001	92
After removing Highly influencing studies	3.34	1.65, 5.03	0.0001	93
Other models [Western diet, tumour, colitis]	0.33	− 0.57, 1.24	0.47	86
After removing Highly influencing studies	0.17	− 0.49,0.83	0.61	72

Pooled effect sizes and 95% CI were determined using Random effects model.

*SMD* Standardised mean difference, *I*^2^ Heterogeneity.

A remarkable rise in propionic acid was observed in the studies followed for a longer period i.e., ≥ 4 weeks of anthocyanin intervention (SMD: 2.40, 95% CI 1.34, 3.47 µmole/gm; I^2^: 90%; *P* = 0.0001) compared to the studies followed for less than 4 weeks (SMD: − 0.08, 95% CI − 1.22, 1.06 µmole/gm; I^2^: 77%; *P* = 0.89) (Table 7; Supplemental Fig. 10 and 11). The study subjects showed a significant rise in propionic acid when a higher dose was supplemented (SMD: 4.15, 95% CI 0.73, 7.57 µmole/gm; I^2^: 90%; *P* = 0.02) compared to the lower dose (SMD: 2.03, 95% CI 0.16, 3.91 µmole/gm; I^2^: 83%; *P* = 0.03). The propionic acid levels were significantly increased in the subjects with high-fat diet-induced obesity (SMD: 4.60, 95% CI 2.30, 6.90 µmole/gm; I^2^: 95%; *P* = 0.0001) in comparison to other study model type (SMD: 0.19, 95% CI − 0.56, 0.93 µmole/gm; I^2^: 79%; *P* = 0.62).

**Table 7:** All-inclusive, high-influencer subtracted and sub-group [intervention duration, dose, and animal model type] analysis to understand the effects of anthocyanins on the propionic acid.

Study/ Subgroup type	SMD	95% CI [µmole/gm]	*P* value	I^2^[%]
Study duration				
< 4 weeks	1.52	0.01, 3.03	0.05	90
After removing Highly influencing studies	− 0.08	− 1.22, 1.06	0.89	77
≥ 4 weeks	2.82	1.74, 3.91	0.00001	92
After removing Highly influencing studies	2.40	1.34, 3.47	0.00001	90
Dose of anthocyanin				
Lower Dose	2.39	0.48, 4.30	0.01	90
After removing Highly influencing studies	2.03	0.16, 3.91	0.03	83
Higher Dose	4.24	1.60, 6.87	0.002	93
After removing Highly influencing studies	4.15	0.73, 7.57	0.02	90
Study model type				
High fat diet model	5.27	3.38, 7.16	0.00001	94
After removing Highly influencing studies	4.60	2.30, 6.90	0.0001	95
Other models [Western diet, tumour, colitis]	0.65	− 0.50, 1.45	0.11	84
After removing Highly influencing studies	0.19	− 0.56, 0.93	0.62	79

Pooled effect sizes and 95% CI were determined using Random effects model.

*SMD* Standardised mean difference, *I*^2^ = Heterogeneity.

The data quality of the SCFA meta-analysis was also evaluated by Grade Tool (Supplemental Fig. 12) and showed moderate heterogeneity that is serious inconsistency.

### Publication bias

The publication bias in our study was predicted by applying Egger’s test for a regression intercept and Begg and Mazumdar’s test for rank correlation by using the Trim-Fill method. The funnel plot for Figure S1 shows no evidence of publication bias. For FIR/BAC ratio Egger’s test for a regression intercept gave a *p*-value of 0.6873. Begg and Mazumdar’s test for rank correlation showed a *p*-value of 0.6612, indicating no evidence of publication bias. In the case of SCFA, the *p*-value of Egger’s test for a regression intercept and Begg and Mazumdar’s test for rank correlation is > 0.05, which indicates no publication bias exists in the studies (Table 8 and Supplemental Fig. 13A–D).

**Table 8:** Data stability analysis of the studies using Egger’s regression and Beggs test.

Treatment	SMD	Reg.*P*	Begg.*P*
FIR/BAC	− 0.1051	0.6873	0.6612
Acetic Acid	0.7288	0.7827	0.9700
Butanoic Acid	0.7407	0.7498	0.6072
Propionic Acid	0.0418	0.9816	0.9715

*The test is not significant [Reg.*P* < 0.001], indicating funnel plot asymmetry.

### Research trends related to anthocyanin

To depict the active collaborations in anthocyanin, gut microbiota, and SCFA research, we tried to detect the network level among the authors (Supplemental Fig. 14A,B). We selected authors with minimum criteria of 10 articles in the chosen field and observed 14 clusters represented in the author network. Out of 46,427 authors, 168 fulfilled the minimum criteria. Supplemental Fig. 14A represents the network visualization among authors, while Supplemental Fig. 14B represents the overlay visualization year-wise work. It indicates that Chen, and Zhang, are the leading researcher in anthocyanin, gut microbiota, and SCFA-related studies, with 37 and 35 articles. Most research work relevant to anthocyanin and gut studies has been carried out recently, i.e., between 2018 and 2022 (Supplemental Fig. 14B).

We also attempted to track the institution and department collaborations through visualization analysis (Supplemental Fig. 15A,B). Out of 21,846 organizations, only 16 met the threshold criteria, i.e., each with a minimum of two articles. These constituted 5 clusters (Supplemental Fig. 15A). This analysis shows that the microbiology laboratory at Wageningen University in the Netherlands published the most articles^18^, followed by the State key laboratory of animal nutrition at China Agricultural University in Beijing, which published 17 papers. Both are among the top institutions working on anthocyanin and gut microbiota (Supplemental Fig. 15A).

On the other hand, when we performed the independent analysis of the same organizations, 905 out of 21,846 fulfilled the criteria. The results reveal the same observations, even with no linkages (Supplemental Fig. 15A,B). On visualizing the year-wise work of organizations, it depicted that most of the collaborative studies were carried out in the 2014–2016 year by top working institutes, and independent research was carried out in recent years (Supplemental Fig. 15B and 14B).

## Discussion

Edible parts of plants carry several health promoting compounds like, proteins, minerals, vitamins and coloured anthocyanins^70–72^. Numerous studies have discovered the health-promoting properties of anthocyanin-rich foods. Anthocyanins have anti-obesity properties, as they help to maintain energy balance and satiety while inhibiting the accumulation of body fat and the development of insulin resistance, dyslipidemia, and inflammation^73,74^. A diet of anthocyanin-rich fruits and vegetables substantially influences the gut flora^13,75^. After being consumed, anthocyanins have limited bioavailability in the body due to their resistance to complete absorption. Five percent to ten percent of total polyphenol consumption is absorbed in the small intestine. More importantly, most dietary anthocyanins arrive intact in the colon, where they may interact with the microbiota and undergo biotransformation before being absorbed via the intestinal mucosa^76^. This systematic review and meta-analysis demonstrated that dietary anthocyanin supplementation profoundly improves rodent models' gut health biomarkers (Fir/Bac and SCFAs). This finding was supported by studies carried out after cut off time limit of this studies^77–81^.

Several studies have shown that obesity is associated with the gut microbiome, which differs between obese and lean animals. The gut health biomarker Fir/ Bac ratio is relevant in human gut microbiota composition. According to certain research articles, the Fir/Bac ratio is a defining characteristic of obesity. Current meta-analyses revealed that anthocyanins effectively reduced the Fir/Bac ratio and mitigated the gut dysbiosis induced by high-fat diet-induced obesity and other factors. Anthocyanin intervention time and dose had a substantial impact on the Fir/Bac ratio in a variety of ways. The impact was more pronounced when the anthocyanin-rich diet was followed for a more extended period and at larger dosages. Our data analysis from rodent models will also help future investigators with the utility of rodent research in understanding the effect of anthocyanins on human models and planning such clinical trials.

Gut health biomarker SCFAs also have significant relevance in human gut microbiota composition. The healthy gut microbiota metabolizes indigestible dietary components to SCFAs^82,83^. The present meta-analysis of laboratory studies on rodents found that anthocyanin-rich diet interventions efficiently improved the gut's SCFAs, including acetic, propionic, and butyric acid profiles. Here also, the longer duration of the anthocyanin-rich diet intervention was more efficient in enhancing the levels of all three main SCFAs. Similarly, the higher dosage of the anthocyanin-rich food intervention was more effective. Aside from that, anthocyanins had more significant impacts on the concentrations of all SCFAs in high-fat diet-induced obesity models than in other disease models.

During meta-analysis, it was observed that a few studies with wider cumulative interval values had more influence on the overall results than a large number of normal studies. Therefore, additional analysis was carried out after removing such studies. Thus, all the analyses were carried out without such studies, and we recommend the same. This improved the outcomes of the meta-analysis. We also noticed substantial methodological and experimental variances in the research. Animal care procedures, oral dosing, and water purification protocols are some examples of unbiased observed variables that must be recorded. Since these factors significantly affect therapy outcomes^9^.

Publication bias is an important parameter in meta-analysis. It includes time lag, duplication, outcome reporting, linguistics, etc. Many electronic databases are examined to eliminate the likelihood of publication bias. To eliminate data supply bias, we employ individual searches and extractions. Participant differences, as well as the intervention's intensity and duration, all contributed to variability. The individuals' health, other therapies they were receiving simultaneously, supplement doses and contents, follow-up durations, treatment modalities, and so on all differed significantly among the trials. These variations may have had a significant role in the funnel plot's original asymmetry. The appearance of an asymmetrical funnel plot is purely coincidental^84,85^. The Trim-Fill correction method made minor changes to all studies, and associated funnel plots revealed a symmetrical distribution of SE and SMD with *p*-values greater than 0.05. The funnel plot indicated that the studies chosen for our research are not biased. Additionally, both Begg's and Egger's tests produced non-significant *P*-values [*P* > 0.05], further supporting the non-existence of any substantial systematic publishing bias in our study. It has also been observed that the discrepancy displayed by the GRADE tool is significant only when it affects confidence in the results concerning a specific decision. Even if the inconsistency is significant, it may still maintain confidence in the conclusion of a particular decision^86^.

The variability is significant, but the disparities between small and large treatment effects could be the source of the substantial heterogeneity.

For the first time, a comprehensive meta-analysis of the influence of anthocyanins on the Fir/Bac ratio and the concentrations of three main SCFAs, acetic acid, propionic acid, and butanoic acid, was performed. Bibliographic coupling analysis of leading researchers and institutes indicated that most research work relevant to anthocyanin and gut studies had recently been carried out in animal models, i.e., between 2018 and 2022. It is envisaged that several such human studies will be published in the near future to validate that current finding.

However, some important qualifiers to this study should be mentioned. As a limitation, PROSPERO, a central international database platform that helps to eliminate data duplication and reduces the chance for reporting bias by permitting comparison of the finished review with what was planned in the protocol, was not notified that this study was being conducted. Furthermore, the substantial amount of missing data for published studies and the exclusion of studies with incomplete data diminish the statistical power of our meta-analysis.

## Supplementary Information

Supplementary Information 1.

Supplementary Figures.

Supplementary Tables.

## Data Availability

The data we used can be found in the references listed and also given in the attached supplementary files. All the figures represented in this manuscript have been produced by authors itself.

## Abbreviations

Fir/Bac: *Firmicutes/Bacteroides*
SCFAs: Short chain fatty acids
SMD: Standardized mean difference
CI: Confidence interval
