# Supplementary material for: Effect of anthocyanins on gut health markers, Firmicutes-Bacteroidetes ratio and short-chain fatty acids: a systematic review via meta-analysis
Source: Sci Rep. 2023 Jan 31;13:1729. doi: 10.1038/s41598-023-28764-0 (PMC9889808; doi:10.1038/s41598-023-28764-0)
Supplement: Supplementary file 2 — Supplementary Figures. [file 41598_2023_28764_MOESM2_ESM.pdf]

Effect of anthocyanins on gut health markers, *Firmicutes-Bacteroidetes* ratio and short-chain fatty acids: A systematic review via meta-analysis

Payal Kapoor

Study or Subgroup	Experimental Mean	Experimental SD	Experimental Total	Control Mean	Control SD	Control Total	Weight	Std. Mean Difference IV, Random, 95% CI
Chen et al., 2022 (a)	8.2	1.55	8	5.6	2.2	8	2.8%	1.29 [0.18, 2.40]
Chen et al., 2022 (b)	5.7	1.4	8	5.6	2.2	8	2.8%	0.05 [-0.93, 1.03]
Chen et al., 2022 (c)	9.5	1.65	8	5.6	2.2	8	2.7%	1.90 [0.66, 3.13]
Chen et al., 2022 (d)	3.6	1.3	8	5.6	2.2	8	2.8%	-1.05 [-2.11, 0.02]
Cremonini et al., 2019	48.27586	23.4451	6	40.51724	18.29862	6	2.7%	0.34 [-0.80, 1.48]
Han et al., 2020	3.167095	1.15807	5	1.789203	0.413874	5	2.6%	1.43 [-0.05, 2.91]
Heyman-Lindén et al., 2017	0.212903	0.07606	8	1.896774	0.802817	8	2.6%	-2.79 [-4.28, -1.31]
Kapoor et al, 2022 (a)	0.368029	0.025413	3	1.07	0.359	3	2.0%	-2.21 [-4.87, 0.46]
Kapoor et al, 2022 (b)	0.60119	0.067725	3	0.628582	0.18476	3	2.5%	-0.16 [-1.76, 1.45]
Kapoor et al, 2022 (c)	0.5147	0.1235	3	0.628582	0.18476	3	2.5%	-0.58 [-2.28, 1.12]
Kapoor et al, 2022 (d)	0.4685	0.116	3	1.07	0.359	3	2.2%	-1.80 [-4.17, 0.56]
Lin et al., 2020	0.863787	0.21262	3	2.697674	0.225914	3	0.8%	-6.69 [-13.34, -0.03]
Liu et al, 2021 (a)	1	0.34	9	0.8	0.29	9	2.8%	0.60 [-0.35, 1.55]
Liu et al, 2021 (b)	0.58	0.22	9	0.8	0.29	9	2.8%	-0.81 [-1.79, 0.16]
Liu et al, 2021 (c)	0.8	0.29	9	0.8	0.29	9	2.8%	0.00 [-0.92, 0.92]
Liu et al, 2021 (d)	0.4	0.1	9	0.8	0.29	9	2.7%	-1.76 [-2.89, -0.63]
Liu et al, 2022 (a)	2.18	0.25	12	2.79	0.495	12	2.8%	-1.50 [-2.43, -0.58]
Liu et al, 2022 (b)	1.9	0.21	12	2.79	0.495	12	2.8%	-2.26 [-3.32, -1.20]
Liu et al, 2022 (c)	1.1	0.15	12	2.79	0.495	12	2.6%	-4.46 [-6.06, -2.87]
Liu et al., 2020 (a)	0.593123	0.11927	6	0.485673	0.084885	6	2.7%	0.96 [-0.27, 2.18]
Liu et al., 2020 (b)	0.8214	0.7684	6	0.485673	0.084885	6	2.7%	0.57 [-0.60, 1.73]
Liu et al., 2021 (e)	9.827563	1.30921	6	7.782219	1.737867	6	2.7%	1.23 [-0.05, 2.51]
Marques et al., 2018	1.21371	0.09016	5	1.165323	0.054098	5	2.7%	0.59 [-0.70, 1.87]
Nakano et al., 2020	0.3	0.22361	5	1.19	0.447214	5	2.5%	-2.27 [-4.06, -0.49]
Patricia Diez-Echave et al., 2020 (a)	4	0.5	10	16	1.3	10	1.4%	-11.67 [-15.80, -7.54]
Patricia Diez-Echave et al., 2020 (b)	1.7	0.3	10	16	1.3	10	1.1%	-14.52 [-19.61, -9.42]
Patricia Diez-Echave et al., 2020 (c)	1.3	0.2	10	16	1.3	10	1.1%	-15.14 [-20.45, -9.83]
Peng et al., 2019 (a)	0.377049	0.25	10	0.464481	0.038251	10	2.8%	-0.47 [-1.36, 0.42]
Peng et al., 2019 (b)	0.174863	0.10246	10	0.464481	0.038251	10	2.6%	-3.59 [-5.11, -2.07]
Rodríguez-Daza et al., 2020 (a)	5.2	1.1	12	3.9	0.7	12	2.8%	1.36 [0.46, 2.27]
Rodríguez-Daza et al., 2020 (b)	5	1	12	3.9	0.7	12	2.8%	1.23 [0.34, 2.12]
Rodríguez-Daza et al., 2020 (c)	4.3	0.9	12	3.9	0.7	12	2.8%	0.48 [-0.33, 1.29]
Song et al., 2021	2.004843	0.36804	4	2.498789	0.40678	4	2.6%	-1.11 [-2.69, 0.47]
Tian et al., 2021 (a)	4.3	0.5	8	10.3	1.5	8	2.2%	-5.07 [-7.32, -2.82]
Tian et al., 2021 (b)	4	0.6	8	10.3	1.5	8	2.2%	-5.21 [-7.51, -2.91]
Tian et al., 2021 (c)	3.7	0.8	8	10.3	1.5	8	2.2%	-5.19 [-7.48, -2.90]
Wang et al., 2020 (a)	1.4	0.1	12	2.5	0.25	12	2.4%	-5.58 [-7.48, -3.68]
Wang et al., 2020 (b)	1.6	0.1	12	2.5	0.25	12	2.5%	-4.56 [-6.19, -2.94]
Wang et al., 2020 (c)	0.7	0.075	12	2.5	0.25	12	1.9%	-9.42 [-12.44, -6.39]
Xu et al., 2019	0.88712	0.3112	7	2.7156	0.2412	7	1.9%	-6.15 [-9.03, -3.26]
Zhong et al., 2020	0.918047	0.99407	9	1.314103	0.975495	9	2.8%	-0.38 [-1.32, 0.55]
**Total (95% CI)**			**332**			**332**	**100.0%**	**-1.80 [-2.48, -1.12]**

Heterogeneity: Tau² = 4.08; Chi² = 409.44, df = 40 (P < 0.00001); I² = 90%
Test for overall effect: Z = 5.17 (P < 0.00001)

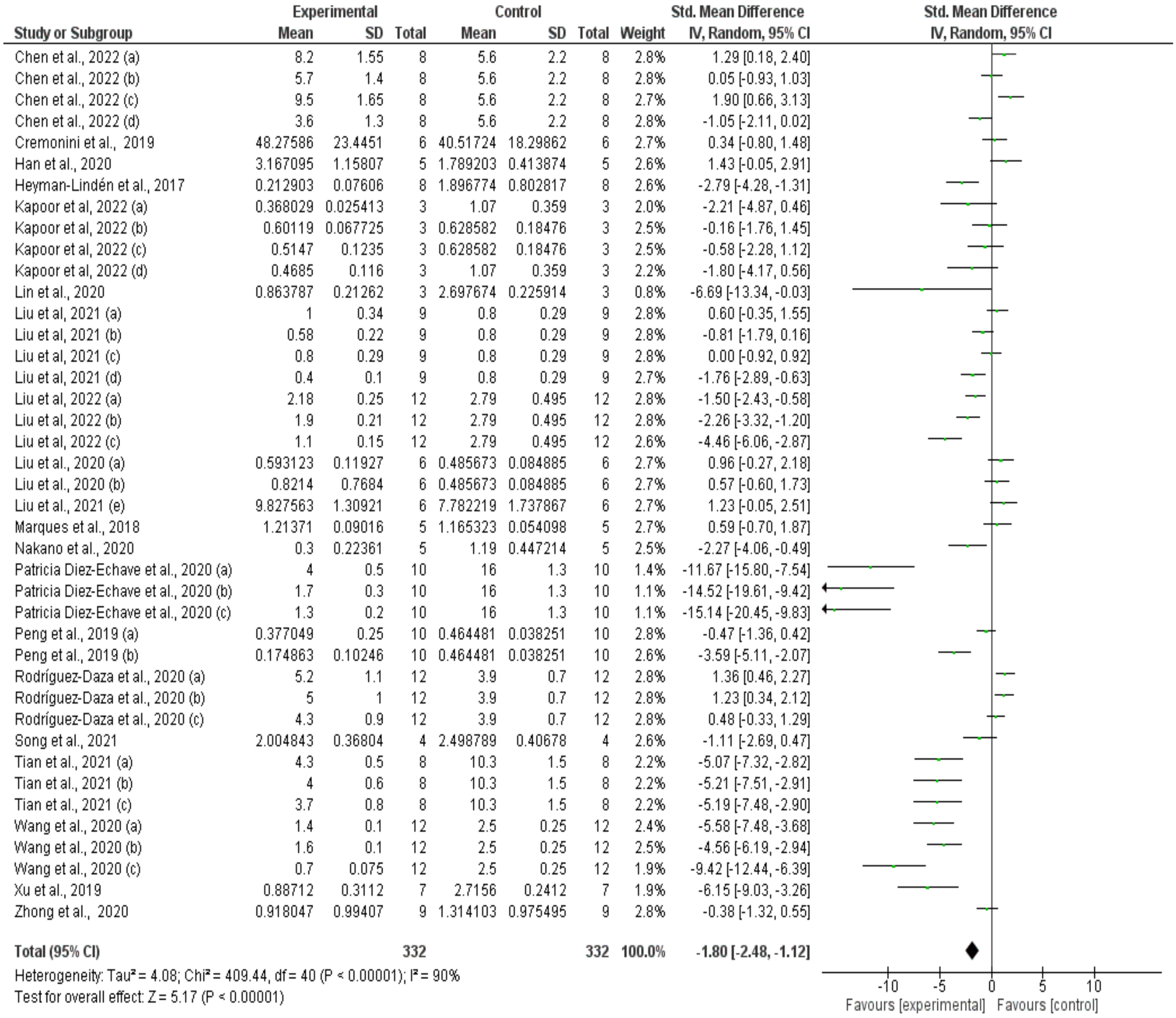

S Figure 1: Forest plot of studies investigating the effect of anthocyanin supplementation on the *Firmicutes* to *Bacteroidetes* ratio (Fir/Bac). Pooled effect estimates (diamonds) for Fir/Bac are shown. Values are standardized mean differences with 95% CIs determined with the use of random-effects models. Heterogeneity was quantified by I^2, inverse variance and standardised mean difference (SMD).

Study or Subgroup	Experimental Mean	Experimental SD	Experimental Total	Control Mean	Control SD	Control Total	Weight	Std. Mean Difference IV, Random, 95% CI
**2.1.1 Study less than 10 week**								
Chen et al., 2022 (a)	8.2	1.55	8	5.6	2.2	8	1.1%	1.29 [0.18, 2.40]
Chen et al., 2022 (b)	5.7	1.4	8	5.6	2.2	8	1.1%	0.05 [-0.93, 1.03]
Chen et al., 2022 (c)	9.5	1.65	8	5.6	2.2	8	1.1%	1.90 [0.66, 3.13]
Chen et al., 2022 (d)	3.6	1.3	8	5.6	2.2	8	1.1%	-1.05 [-2.11, 0.02]
Liu et al., 2020 (a)	0.593123	0.11927	6	0.485673	0.084885	6	1.1%	0.96 [-0.27, 2.18]
Liu et al., 2020 (b)	0.8214	0.7684	6	0.485673	0.084885	6	1.1%	0.57 [-0.60, 1.73]
Patricia Diez-Echave et al., 2020 (a)	4	0.5	10	16	1.3	10	0.6%	-11.67 [-15.80, -7.54]
Patricia Diez-Echave et al., 2020 (b)	1.7	0.3	10	16	1.3	10	0.5%	-14.52 [-19.61, -9.42]
Patricia Diez-Echave et al., 2020 (c)	1.3	0.2	10	16	1.3	10	0.5%	-15.14 [-20.45, -9.83]
Peng et al., 2019 (a)	0.377049	0.25	10	0.464481	0.038251	10	1.1%	-0.47 [-1.36, 0.42]
Peng et al., 2019 (b)	0.174863	0.10246	10	0.464481	0.038251	10	1.0%	-3.59 [-5.11, -2.07]
Rodríguez-Daza et al., 2020 (a)	5.2	1.1	12	3.9	0.7	12	1.1%	1.36 [0.46, 2.27]
Rodríguez-Daza et al., 2020 (b)	5	1	12	3.9	0.7	12	1.1%	1.23 [0.34, 2.12]
Rodríguez-Daza et al., 2020 (c)	4.3	0.9	12	3.9	0.7	12	1.1%	0.48 [-0.33, 1.29]
Xu et al., 2019	0.88712	0.3112	7	2.7156	0.2412	7	0.8%	-6.15 [-9.03, -3.26]
**Subtotal (95% CI)**			**137**			**137**	**14.4%**	**-1.49 [-2.64, -0.33]**
Heterogeneity: Tau² = 4.20; Chi² = 170.58, df = 14 (P < 0.00001); I² = 92%								
Test for overall effect: Z = 2.53 (P = 0.01)								
**2.1.2 study equal to or more than 10 week**								
Cremonini et al., 2019	5.956	1.125	6	40.51724	7.12	6	0.7%	-6.26 [-9.52, -3.00]
Han et al., 2020	3.167095	1.15807	5	1.789203	0.413874	5	1.0%	1.43 [-0.05, 2.91]
Heyman-Lindén et al., 2017	0.212903	0.07606	8	1.896774	0.802817	8	1.0%	-2.79 [-4.28, -1.31]
Kapoor et al, 2022 (a)	0.368029	0.025413	3	1.07	0.359	3	0.8%	-2.21 [-4.87, 0.46]
Kapoor et al, 2022 (b)	0.60119	0.067725	3	0.628582	0.18476	3	1.0%	-0.16 [-1.76, 1.45]
Kapoor et al, 2022 (c)	0.5147	0.1235	3	0.628582	0.18476	3	1.0%	-0.58 [-2.28, 1.12]
Kapoor et al, 2022 (d)	0.4685	0.116	3	1.07	0.359	3	0.9%	-1.80 [-4.17, 0.56]
Lin et al., 2020	5.831202	2.19949	8	12.12276	2.058824	8	1.0%	-2.79 [-4.28, -1.31]
Liu et al, 2021 (a)	1	0.34	9	0.8	0.29	9	1.1%	0.60 [-0.35, 1.55]
Liu et al, 2021 (b)	0.58	0.22	9	0.8	0.29	9	1.1%	-0.81 [-1.79, 0.16]
Liu et al, 2021 (c)	0.8	0.29	9	0.8	0.29	9	1.1%	0.00 [-0.92, 0.92]
Liu et al, 2021 (d)	0.4	0.1	9	0.8	0.29	9	1.1%	-1.76 [-2.89, -0.63]
Liu et al, 2022 (a)	2.18	0.25	12	2.79	0.495	12	1.1%	-1.50 [-2.43, -0.58]
Liu et al, 2022 (b)	1.9	0.21	12	2.79	0.495	12	1.1%	-2.26 [-3.32, -1.20]
Liu et al, 2022 (c)	1.1	0.15	12	2.79	0.495	12	1.0%	-4.46 [-6.06, -2.87]
Liu et al., 2021 (e)	9.827563	1.30921	6	7.782219	1.737867	6	1.1%	1.23 [-0.05, 2.51]
Marques et al., 2018	1.21371	0.09016	5	1.165323	0.054098	5	1.1%	0.59 [-0.70, 1.87]
Nakano et al., 2020	0.3	0.22361	5	1.19	0.447214	5	1.0%	-2.27 [-4.06, -0.49]
Song et al., 2021	2.004843	0.36804	4	2.498789	0.40678	4	1.0%	-1.11 [-2.69, 0.47]
Tian et al., 2021 (a)	4.3	0.5	8	10.3	1.5	8	0.9%	-5.07 [-7.32, -2.82]
Tian et al., 2021 (b)	4	0.6	8	10.3	1.5	8	0.9%	-5.21 [-7.51, -2.91]
Tian et al., 2021 (c)	3.7	0.8	8	10.3	1.5	8	0.9%	-5.19 [-7.48, -2.90]
Wang et al., 2020 (a)	1.4	0.1	12	2.5	0.25	12	1.0%	-5.58 [-7.48, -3.68]
Wang et al., 2020 (b)	1.6	0.1	12	2.5	0.25	12	1.0%	-4.56 [-6.19, -2.94]
Wang et al., 2020 (c)	0.7	0.075	12	2.5	0.25	12	0.8%	-9.42 [-12.44, -6.39]
Zhong et al., 2020	0.918047	0.99407	9	1.314103	0.975495	9	1.1%	-0.38 [-1.32, 0.55]
**Subtotal (95% CI)**			**200**			**200**	**26.0%**	**-2.16 [-2.96, -1.36]**
Heterogeneity: Tau² = 3.57; Chi² = 199.76, df = 25 (P < 0.00001); I² = 87%								
Test for overall effect: Z = 5.28 (P < 0.00001)								
**2.1.3 Lower Dose**								
Chen et al., 2022 (a)	8.2	1.55	8	5.6	2.2	8	1.1%	1.29 [0.18, 2.40]
Chen et al., 2022 (c)	9.5	1.65	8	5.6	2.2	8	1.1%	1.90 [0.66, 3.13]
Kapoor et al, 2022 (a)	0.368029	0.025413	3	1.07	0.359	3	0.8%	-2.21 [-4.87, 0.46]
Kapoor et al, 2022 (c)	0.5147	0.1235	3	0.628582	0.18476	3	1.0%	-0.58 [-2.28, 1.12]
Liu et al, 2021 (a)	0.593123	0.11927	6	0.485673	0.084885	6	1.1%	0.96 [-0.27, 2.18]
Liu et al, 2021 (c)	8.2	1.55	8	5.6	2.2	8	1.1%	1.29 [0.18, 2.40]
Liu et al, 2022 (a)	9.5	1.65	8	5.6	2.2	8	1.1%	1.90 [0.66, 3.13]
Liu et al., 2020 (a)	2.18	0.25	12	2.79	0.495	12	1.1%	-1.50 [-2.43, -0.58]
Patricia Diez-Echave et al., 2020 (a)	4	0.5	10	16	1.3	10	0.6%	-11.67 [-15.80, -7.54]
Rodríguez-Daza et al., 2020 (a)	1.4	0.1	12	2.5	0.25	12	1.0%	-5.58 [-7.48, -3.68]
Tian et al., 2021 (a)	4.3	0.5	8	10.3	1.5	8	0.9%	-5.07 [-7.32, -2.82]
Wang et al., 2020 (a)	0.8	0.29	9	0.8	0.29	9	1.1%	0.00 [-0.92, 0.92]
**Subtotal (95% CI)**			**95**			**95**	**12.0%**	**-1.12 [-2.47, 0.23]**
Heterogeneity: Tau² = 4.92; Chi² = 127.23, df = 11 (P < 0.00001); I² = 91%								
Test for overall effect: Z = 1.62 (P = 0.11)								
**2.1.4 Higher Dose**								
Chen et al., 2022 (b)	5.7	1.4	8	5.6	2.2	8	1.1%	0.05 [-0.93, 1.03]
Chen et al., 2022 (d)	3.6	1.3	8	5.6	2.2	8	1.1%	-1.05 [-2.11, 0.02]
Kapoor et al, 2022 (b)	0.60119	0.067725	3	0.628582	0.18476	3	1.0%	-0.16 [-1.76, 1.45]
Kapoor et al, 2022 (d)	0.4685	0.116	3	1.07	0.359	3	0.9%	-1.80 [-4.17, 0.56]
Liu et al, 2021 (b)	0.58	0.22	9	0.8	0.29	9	1.1%	-0.81 [-1.79, 0.16]
Liu et al, 2021 (d)	0.4	0.1	9	0.8	0.29	9	1.1%	-1.76 [-2.89, -0.63]
Liu et al, 2022 (c)	1.1	0.15	12	2.79	0.495	12	1.0%	-4.46 [-6.06, -2.87]
Liu et al., 2020 (b)	0.8214	0.7684	6	0.485673	0.084885	6	1.1%	0.57 [-0.60, 1.73]
Patricia Diez-Echave et al., 2020 (c)	1.7	0.3	10	16	1.3	10	0.5%	-14.52 [-19.61, -9.42]
Rodríguez-Daza et al., 2020 (c)	5	1	12	3.9	0.7	12	1.1%	1.23 [0.34, 2.12]
Tian et al., 2021 (c)	3.7	0.8	8	10.3	1.5	8	0.9%	-5.19 [-7.48, -2.90]
Wang et al., 2020 (c)	0.7	0.075	12	2.5	0.25	12	0.8%	-9.42 [-12.44, -6.39]
**Subtotal (95% CI)**			**100**			**100**	**11.7%**	**-2.33 [-3.69, -0.98]**
Heterogeneity: Tau² = 4.83; Chi² = 126.00, df = 11 (P < 0.00001); I² = 91%								
Test for overall effect: Z = 3.37 (P = 0.0007)								
**2.1.5 High Fat/Cholesterol Model**								
Chen et al., 2022 (a)	8.2	1.55	8	5.6	2.2	8	1.1%	1.29 [0.18, 2.40]
Chen et al., 2022 (b)	5.7	1.4	8	5.6	2.2	8	1.1%	0.05 [-0.93, 1.03]
Chen et al., 2022 (c)	9.5	1.65	8	5.6	2.2	8	1.1%	1.90 [0.66, 3.13]
Chen et al., 2022 (d)	3.6	1.3	8	5.6	2.2	8	1.1%	-1.05 [-2.11, 0.02]
Han et al., 2020	3.167095	1.15807	5	1.789203	0.413874	5	1.0%	1.43 [-0.05, 2.91]
Heyman-Lindén et al., 2017	0.212903	0.07606	8	1.896774	0.802817	8	1.0%	-2.79 [-4.28, -1.31]
Lin et al., 2020	5.831202	2.19949	8	12.12276	2.058824	8	1.0%	-2.79 [-4.28, -1.31]
Liu et al, 2022 (a)	2.18	0.25	12	2.79	0.495	12	1.1%	-1.50 [-2.43, -0.58]
Liu et al, 2022 (b)	1.9	0.21	12	2.79	0.495	12	1.1%	-2.26 [-3.32, -1.20]
Liu et al, 2022 (c)	1.1	0.15	12	2.79	0.495	12	1.0%	-4.46 [-6.06, -2.87]
Liu et al., 2021 (e)	9.827563	1.30921	6	7.782219	1.737867	6	1.1%	1.23 [-0.05, 2.51]
Marques et al., 2018	1.21371	0.09016	5	1.165323	0.054098	5	1.1%	0.59 [-0.70, 1.87]
Patricia Diez-Echave et al., 2020 (a)	4	0.5	10	16	1.3	10	0.6%	-11.67 [-15.80, -7.54]
Patricia Diez-Echave et al., 2020 (b)	1.7	0.3	10	16	1.3	10	0.5%	-14.52 [-19.61, -9.42]
Patricia Diez-Echave et al., 2020 (c)	1.3	0.2	10	16	1.3	10	0.5%	-15.14 [-20.45, -9.83]
Rodríguez-Daza et al., 2020 (a)	5.2	1.1	12	3.9	0.7	12	1.1%	1.36 [0.46, 2.27]
Rodríguez-Daza et al., 2020 (b)	5	1	12	3.9	0.7	12	1.1%	1.23 [0.34, 2.12]
Rodríguez-Daza et al., 2020 (c)	4.3	0.9	12	3.9	0.7	12	1.1%	0.48 [-0.33, 1.29]
Song et al., 2021	2.004843	0.36804	4	2.498789	0.40678	4	1.0%	-1.11 [-2.69, 0.47]
Tian et al., 2021 (a)	4.3	0.5	8	10.3	1.5	8	0.9%	-5.07 [-7.32, -2.82]
Tian et al., 2021 (b)	4	0.6	8	10.3	1.5	8	0.9%	-5.21 [-7.51, -2.91]
Tian et al., 2021 (c)	3.7	0.8	8	10.3	1.5	8	0.9%	-5.19 [-7.48, -2.90]
Wang et al., 2020 (c)	0.7	0.075	12	2.5	0.25	12	0.8%	-9.42 [-12.44, -6.39]
Xu et al., 2019	0.88712	0.3112	7	2.7156	0.2412	7	0.8%	-6.15 [-9.03, -3.26]
Zhong et al., 2020	0.918047	0.99407	9	1.314103	0.975495	9	1.1%	-0.38 [-1.32, 0.55]
**Subtotal (95% CI)**			**222**			**222**	**24.2%**	**-2.31 [-3.31, -1.31]**
Heterogeneity: Tau² = 5.50; Chi² = 319.63, df = 24 (P < 0.00001); I² = 92%								
Test for overall effect: Z = 4.53 (P < 0.00001)								
**2.1.6 Other Animal Model**								
Kapoor et al, 2022 (a)	0.368029	0.025413	3	1.07	0.359	3	0.8%	-2.21 [-4.87, 0.46]
Kapoor et al, 2022 (b)	0.60119	0.067725	3	0.628582	0.18476	3	1.0%	-0.16 [-1.76, 1.45]
Kapoor et al, 2022 (c)	0.5147	0.1235	3	0.628582	0.18476	3	1.0%	-0.58 [-2.28, 1.12]
Kapoor et al, 2022 (d)	0.4685	0.116	3	1.07	0.359	3	0.9%	-1.80 [-4.17, 0.56]
Liu et al., 2020 (a)	0.593123	0.11927	6	0.485673	0.084885	6	1.1%	0.96 [-0.27, 2.18]
Liu et al., 2020 (b)	0.8214	0.7684	6	0.485673	0.084885	6	1.1%	0.57 [-0.60, 1.73]
Nakano et al., 2020	0.3	0.22361	5	1.19	0.447214	5	1.0%	-2.27 [-4.06, -0.49]
Peng et al., 2019 (a)	0.377049	0.25	10	0.464481	0.038251	10	1.1%	-0.47 [-1.36, 0.42]
Peng et al., 2019 (b)	0.174863	0.10246	10	0.464481	0.038251	10	1.0%	-3.59 [-5.11, -2.07]
Wang et al., 2020 (a)	1.4	0.1	12	2.5	0.25	12	1.0%	-5.58 [-7.48, -3.68]
Wang et al., 2020 (b)	1.6	0.1	12	2.5	0.25	12	1.0%	-4.56 [-6.19, -2.94]
Wang et al., 2020 (c)	0.7	0.075	12	2.5	0.25	12	0.8%	-9.42 [-12.44, -6.39]
**Subtotal (95% CI)**			**85**			**85**	**11.8%**	**-2.25 [-3.64, -0.86]**
Heterogeneity: Tau² = 5.18; Chi² = 102.03, df = 11 (P < 0.00001); I² = 89%								
Test for overall effect: Z = 3.17 (P = 0.002)								
**Total (95% CI)**			**839**			**839**	**100.0%**	**-2.00 [-2.45, -1.54]**
Heterogeneity: Tau² = 4.60; Chi² = 1118.83, df = 101 (P < 0.00001); I² = 91%								
Test for overall effect: Z = 8.60 (P < 0.00001)								
Test for subgroup differences: Chi² = 3.26, df = 5 (P = 0.66), I² = 0%								

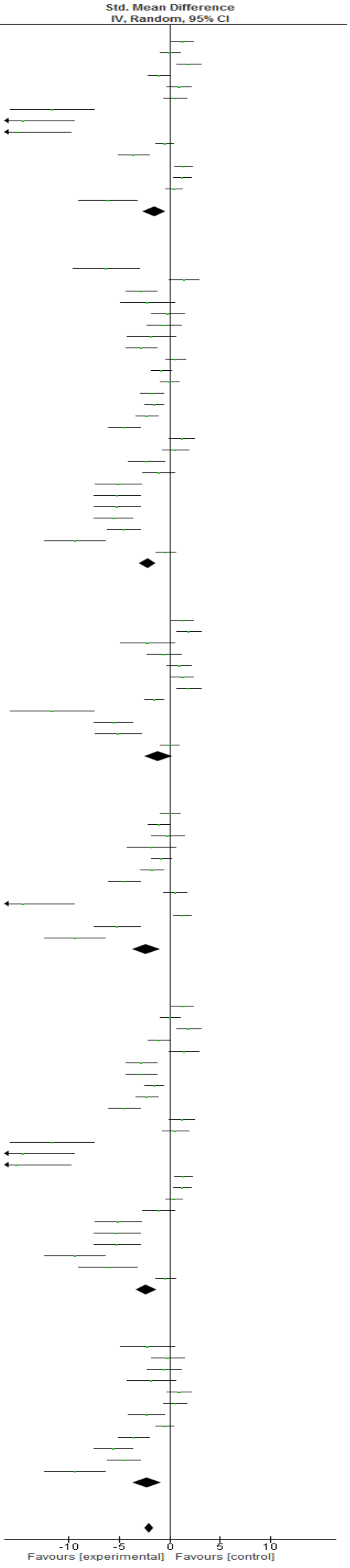

S Figure 2: Forest plot of the sub-group analysis to study effect of anthocyanin supplementation on the *Firmicutes* to *Bacteroidetes* ratio (Fir/Bac). Sub-grouping was on the basis of duration of the study, dose of the anthocyanin given and disease status of the subjects (animal models). Pooled effect estimates (diamonds) for Fir/Bac are shown. Values are standardized mean differences with 95% CIs determined with the use of random-effects models. Heterogeneity was quantified by I^2, inverse variance and standardised mean difference (SMD).

Study or Subgroup	Experimental Mean	Experimental SD	Experimental Total	Control Mean	Control SD	Control Total	Weight	Std. Mean Difference IV, Random, 95% CI
**2.1.1 Study less than 10 week**								
Rodríguez-Daza et al., 2020 (c)	4.3	0.9	12	3.9	0.7	12	1.3%	0.48 [-0.33, 1.29]
Rodríguez-Daza et al., 2020 (b)	5	1	12	3.9	0.7	12	1.3%	1.23 [0.34, 2.12]
Peng et al., 2019 (a)	0.377049	0.25	10	0.464481	0.038251	10	1.3%	-0.47 [-1.36, 0.42]
Rodríguez-Daza et al., 2020 (a)	5.2	1.1	12	3.9	0.7	12	1.3%	1.36 [0.46, 2.27]
Chen et al., 2022 (b)	5.7	1.4	8	5.6	2.2	8	1.2%	0.05 [-0.93, 1.03]
Chen et al., 2022 (d)	3.6	1.3	8	5.6	2.2	8	1.2%	-1.05 [-2.11, 0.02]
Chen et al., 2022 (a)	8.2	1.55	8	5.6	2.2	8	1.2%	1.29 [0.18, 2.40]
Liu et al., 2020 (b)	0.8214	0.7684	6	0.485673	0.084885	6	1.2%	0.57 [-0.60, 1.73]
Liu et al., 2020 (a)	0.593123	0.11927	6	0.485673	0.084885	6	1.2%	0.96 [-0.27, 2.18]
Chen et al., 2022 (c)	9.5	1.65	8	5.6	2.2	8	1.2%	1.90 [0.66, 3.13]
Peng et al., 2019 (b)	0.174863	0.10246	10	0.464481	0.038251	10	1.1%	-3.59 [-5.11, -2.07]
**Subtotal (95% CI)**			**100**			**100**	**13.5%**	**0.30 [-0.43, 1.03]**
Heterogeneity: Tau² = 1.23; Chi² = 54.41, df = 10 (P < 0.00001); I² = 82%								
Test for overall effect: Z = 0.80 (P = 0.42)								
**2.1.2 Study equal to or more than 10 week**								
Liu et al, 2021 (c)	0.8	0.29	9	0.8	0.29	9	1.3%	0.00 [-0.92, 0.92]
Liu et al, 2022 (a)	2.18	0.25	12	2.79	0.495	12	1.3%	-1.50 [-2.43, -0.58]
Zhong et al., 2020	0.918047	0.99407	9	1.314103	0.975495	9	1.3%	-0.38 [-1.32, 0.55]
Liu et al, 2021 (a)	1	0.34	9	0.8	0.29	9	1.3%	0.60 [-0.35, 1.55]
Liu et al, 2021 (b)	0.58	0.22	9	0.8	0.29	9	1.3%	-0.81 [-1.79, 0.16]
Liu et al, 2022 (b)	1.9	0.21	12	2.79	0.495	12	1.2%	-2.26 [-3.32, -1.20]
Liu et al, 2021 (d)	0.4	0.1	9	0.8	0.29	9	1.2%	-1.76 [-2.89, -0.63]
Liu et al., 2021 (e)	9.827563	1.30921	6	7.782219	1.737867	6	1.2%	1.23 [-0.05, 2.51]
Marques et al., 2018	1.21371	0.09016	5	1.165323	0.054098	5	1.2%	0.59 [-0.70, 1.87]
Han et al., 2020	3.167095	1.15807	5	1.789203	0.413874	5	1.1%	1.43 [-0.05, 2.91]
Heyman-Lindén et al., 2017	0.212903	0.07606	8	1.896774	0.802817	8	1.1%	-2.79 [-4.28, -1.31]
Lin et al., 2020	5.831202	2.19949	8	12.12276	2.058824	8	1.1%	-2.79 [-4.28, -1.31]
Song et al., 2021	2.004843	0.36804	4	2.498789	0.40678	4	1.1%	-1.11 [-2.69, 0.47]
Liu et al, 2022 (c)	1.1	0.15	12	2.79	0.495	12	1.1%	-4.46 [-6.06, -2.87]
Kapoor et al, 2022 (b)	0.60119	0.067725	3	0.628582	0.18476	3	1.1%	-0.16 [-1.76, 1.45]
Wang et al., 2020 (b)	1.6	0.1	12	2.5	0.25	12	1.1%	-4.56 [-6.19, -2.94]
Kapoor et al, 2022 (c)	0.5147	0.1235	3	0.628582	0.18476	3	1.1%	-0.58 [-2.28, 1.12]
Nakano et al., 2020	0.3	0.22361	5	1.19	0.447214	5	1.1%	-2.27 [-4.06, -0.49]
Wang et al., 2020 (a)	1.4	0.1	12	2.5	0.25	12	1.0%	-5.58 [-7.48, -3.68]
Tian et al., 2021 (a)	4.3	0.5	8	10.3	1.5	8	0.9%	-5.07 [-7.32, -2.82]
Tian et al., 2021 (c)	3.7	0.8	8	10.3	1.5	8	0.9%	-5.19 [-7.48, -2.90]
Tian et al., 2021 (b)	4	0.6	8	10.3	1.5	8	0.9%	-5.21 [-7.51, -2.91]
Kapoor et al, 2022 (d)	0.4685	0.116	3	1.07	0.359	3	0.9%	-1.80 [-4.17, 0.56]
Kapoor et al, 2022 (a)	0.368029	0.025413	3	1.07	0.359	3	0.8%	-2.21 [-4.87, 0.46]
**Subtotal (95% CI)**			**182**			**182**	**26.6%**	**-1.81 [-2.56, -1.05]**
Heterogeneity: Tau² = 2.91; Chi² = 163.13, df = 23 (P < 0.00001); I² = 86%								
Test for overall effect: Z = 4.68 (P < 0.00001)								
**2.1.3 Lower Dose**								
Rodríguez-Daza et al., 2020 (a)	5.2	1.1	12	3.9	0.7	12	1.3%	1.36 [0.46, 2.27]
Liu et al, 2021 (c)	0.8	0.29	9	0.8	0.29	9	1.3%	0.00 [-0.92, 0.92]
Liu et al, 2022 (a)	2.18	0.25	12	2.79	0.495	12	1.3%	-1.50 [-2.43, -0.58]
Liu et al, 2021 (a)	1	0.34	9	0.8	0.29	9	1.3%	0.60 [-0.35, 1.55]
Chen et al., 2022 (a)	8.2	1.55	8	5.6	2.2	8	1.2%	1.29 [0.18, 2.40]
Liu et al., 2020 (a)	0.593123	0.11927	6	0.485673	0.084885	6	1.2%	0.96 [-0.27, 2.18]
Chen et al., 2022 (c)	9.5	1.65	8	5.6	2.2	8	1.2%	1.90 [0.66, 3.13]
Kapoor et al, 2022 (c)	0.5147	0.1235	3	0.628582	0.18476	3	1.1%	-0.58 [-2.28, 1.12]
Wang et al., 2020 (a)	1.4	0.1	12	2.5	0.25	12	1.0%	-5.58 [-7.48, -3.68]
Tian et al., 2021 (a)	4.3	0.5	8	10.3	1.5	8	0.9%	-5.07 [-7.32, -2.82]
Kapoor et al, 2022 (a)	0.368029	0.025413	3	1.07	0.359	3	0.8%	-2.21 [-4.87, 0.46]
**Subtotal (95% CI)**			**90**			**90**	**12.5%**	**-0.60 [-1.74, 0.54]**
Heterogeneity: Tau² = 3.14; Chi² = 92.51, df = 10 (P < 0.00001); I² = 89%								
Test for overall effect: Z = 1.03 (P = 0.30)								
**2.1.4 Higher Dose**								
Rodríguez-Daza et al., 2020 (c)	4.3	0.9	12	3.9	0.7	12	1.3%	0.48 [-0.33, 1.29]
Liu et al, 2021 (b)	0.58	0.22	9	0.8	0.29	9	1.3%	-0.81 [-1.79, 0.16]
Chen et al., 2022 (b)	5.7	1.4	8	5.6	2.2	8	1.2%	0.05 [-0.93, 1.03]
Chen et al., 2022 (d)	3.6	1.3	8	5.6	2.2	8	1.2%	-1.05 [-2.11, 0.02]
Liu et al, 2021 (d)	0.4	0.1	9	0.8	0.29	9	1.2%	-1.76 [-2.89, -0.63]
Liu et al., 2020 (b)	0.8214	0.7684	6	0.485673	0.084885	6	1.2%	0.57 [-0.60, 1.73]
Liu et al, 2022 (c)	1.1	0.15	12	2.79	0.495	12	1.1%	-4.46 [-6.06, -2.87]
Kapoor et al, 2022 (b)	0.60119	0.067725	3	0.628582	0.18476	3	1.1%	-0.16 [-1.76, 1.45]
Tian et al., 2021 (c)	3.7	0.8	8	10.3	1.5	8	0.9%	-5.19 [-7.48, -2.90]
Kapoor et al, 2022 (d)	0.4685	0.116	3	1.07	0.359	3	0.9%	-1.80 [-4.17, 0.56]
Wang et al., 2020 (c)	0.7	0.075	12	2.5	0.25	12	0.8%	-9.42 [-12.44, -6.39]
**Subtotal (95% CI)**			**90**			**90**	**12.2%**	**-1.79 [-2.95, -0.64]**
Heterogeneity: Tau² = 3.18; Chi² = 87.46, df = 10 (P < 0.00001); I² = 89%								
Test for overall effect: Z = 3.04 (P = 0.002)								
**2.1.5 High Fat/Cholesterol Model**								
Rodríguez-Daza et al., 2020 (c)	4.3	0.9	12	3.9	0.7	12	1.3%	0.48 [-0.33, 1.29]
Rodríguez-Daza et al., 2020 (b)	5	1	12	3.9	0.7	12	1.3%	1.23 [0.34, 2.12]
Rodríguez-Daza et al., 2020 (a)	5.2	1.1	12	3.9	0.7	12	1.3%	1.36 [0.46, 2.27]
Liu et al, 2022 (a)	2.18	0.25	12	2.79	0.495	12	1.3%	-1.50 [-2.43, -0.58]
Zhong et al., 2020	0.918047	0.99407	9	1.314103	0.975495	9	1.3%	-0.38 [-1.32, 0.55]
Chen et al., 2022 (b)	5.7	1.4	8	5.6	2.2	8	1.2%	0.05 [-0.93, 1.03]
Liu et al, 2022 (b)	1.9	0.21	12	2.79	0.495	12	1.2%	-2.26 [-3.32, -1.20]
Chen et al., 2022 (d)	3.6	1.3	8	5.6	2.2	8	1.2%	-1.05 [-2.11, 0.02]
Chen et al., 2022 (a)	8.2	1.55	8	5.6	2.2	8	1.2%	1.29 [0.18, 2.40]
Chen et al., 2022 (c)	9.5	1.65	8	5.6	2.2	8	1.2%	1.90 [0.66, 3.13]
Liu et al., 2021 (e)	9.827563	1.30921	6	7.782219	1.737867	6	1.2%	1.23 [-0.05, 2.51]
Marques et al., 2018	1.21371	0.09016	5	1.165323	0.054098	5	1.2%	0.59 [-0.70, 1.87]
Han et al., 2020	3.167095	1.15807	5	1.789203	0.413874	5	1.1%	1.43 [-0.05, 2.91]
Heyman-Lindén et al., 2017	0.212903	0.07606	8	1.896774	0.802817	8	1.1%	-2.79 [-4.28, -1.31]
Lin et al., 2020	5.831202	2.19949	8	12.12276	2.058824	8	1.1%	-2.79 [-4.28, -1.31]
Song et al., 2021	2.004843	0.36804	4	2.498789	0.40678	4	1.1%	-1.11 [-2.69, 0.47]
Liu et al, 2022 (c)	1.1	0.15	12	2.79	0.495	12	1.1%	-4.46 [-6.06, -2.87]
Tian et al., 2021 (a)	4.3	0.5	8	10.3	1.5	8	0.9%	-5.07 [-7.32, -2.82]
Tian et al., 2021 (c)	3.7	0.8	8	10.3	1.5	8	0.9%	-5.19 [-7.48, -2.90]
Tian et al., 2021 (b)	4	0.6	8	10.3	1.5	8	0.9%	-5.21 [-7.51, -2.91]
**Subtotal (95% CI)**			**173**			**173**	**23.2%**	**-0.94 [-1.78, -0.11]**
Heterogeneity: Tau² = 3.15; Chi² = 183.59, df = 19 (P < 0.00001); I² = 90%								
Test for overall effect: Z = 2.21 (P = 0.03)								
**2.1.6 Other Animal Model**								
Peng et al., 2019 (a)	0.377049	0.25	10	0.464481	0.038251	10	1.3%	-0.47 [-1.36, 0.42]
Liu et al., 2020 (b)	0.8214	0.7684	6	0.485673	0.084885	6	1.2%	0.57 [-0.60, 1.73]
Liu et al., 2020 (a)	0.593123	0.11927	6	0.485673	0.084885	6	1.2%	0.96 [-0.27, 2.18]
Peng et al., 2019 (b)	0.174863	0.10246	10	0.464481	0.038251	10	1.1%	-3.59 [-5.11, -2.07]
Kapoor et al, 2022 (b)	0.60119	0.067725	3	0.628582	0.18476	3	1.1%	-0.16 [-1.76, 1.45]
Wang et al., 2020 (b)	1.6	0.1	12	2.5	0.25	12	1.1%	-4.56 [-6.19, -2.94]
Kapoor et al, 2022 (c)	0.5147	0.1235	3	0.628582	0.18476	3	1.1%	-0.58 [-2.28, 1.12]
Nakano et al., 2020	0.3	0.22361	5	1.19	0.447214	5	1.1%	-2.27 [-4.06, -0.49]
Wang et al., 2020 (a)	1.4	0.1	12	2.5	0.25	12	1.0%	-5.58 [-7.48, -3.68]
Kapoor et al, 2022 (d)	0.4685	0.116	3	1.07	0.359	3	0.9%	-1.80 [-4.17, 0.56]
Kapoor et al, 2022 (a)	0.368029	0.025413	3	1.07	0.359	3	0.8%	-2.21 [-4.87, 0.46]
**Subtotal (95% CI)**			**73**			**73**	**11.9%**	**-1.72 [-2.97, -0.46]**
Heterogeneity: Tau² = 3.74; Chi² = 73.99, df = 10 (P < 0.00001); I² = 86%								
Test for overall effect: Z = 2.69 (P = 0.007)								
**Total (95% CI)**			**708**			**708**	**100.0%**	**-1.15 [-1.55, -0.76]**
Heterogeneity: Tau² = 2.95; Chi² = 739.93, df = 87 (P < 0.00001); I² = 88%								
Test for overall effect: Z = 5.77 (P < 0.00001)								
Test for subgroup differences: Chi² = 20.02, df = 5 (P = 0.001), I² = 75.0%								

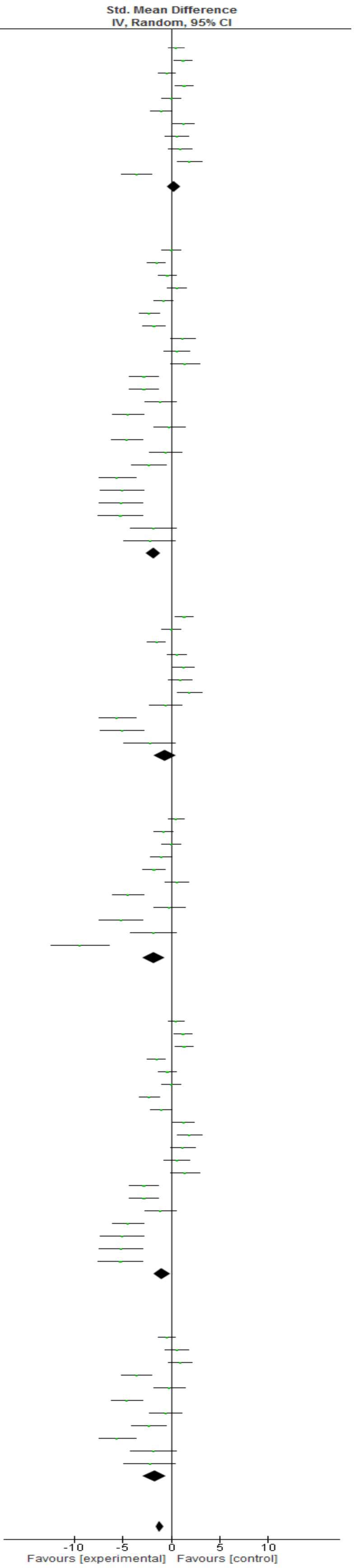

S Figure 3: Forest plot of the sub-group analysis after elimination of high-influencer studies, for understanding the effect of anthocyanin supplementation on the *Firmicutes* to *Bacteroidetes* ratio (Fir/Bac). Sub-grouping was on the basis of duration of the study, dose of the anthocyanin given and disease status of the subjects (animal models). Pooled effect estimates (diamonds) for Fir/Bac are shown. Values are standardized mean differences with 95% CIs determined with the use of random-effects models. Heterogeneity was quantified by I^2, inverse variance and standardised mean difference (SMD).

Question: Should Anthocyanin Rich vs Normal Diet be used for Fir/Bac?

Bibliography: . [Intervention] for [health problem]. Cochrane Database of Systematic Reviews [Year], Issue [Issue].

Quality assessment							Summary of Findings				
Participants (studies) Follow up	Risk of bias	Inconsistency	Indirectness	Imprecision	Publication bias	Overall quality of evidence	Study event rates (%)		Relative effect (95% CI)	Anticipated absolute effects	
							With Normal Diet	With Anthocyanin Rich		Risk with Normal Diet	Risk difference with Anthocyanin Rich (95% CI)
**Fir/Bac** (Better indicated by higher values)											
536 (34 studies)	no serious risk of bias	serious[1]	no serious indirectness	no serious imprecision	undetected	⊕⊕⊕⊕ **HIGH**[1] due to inconsistency, large effect	268	268	-		The mean fir/bac in the intervention groups was **0.89 standard deviations lower** (1.47 to 0.31 lower)

[1] High heterogeneity

S Figure 4: Fir/Bac data quality assessment using GRADE tool.

Study or Subgroup	Experimental Mean	Experimental SD	Experimental Total	Control Mean	Control SD	Control Total	Weight	Std. Mean Difference IV, Random, 95% CI
**1.1.1 Acetic Acid**								
Liu et al., 2022 (c)	11.8792	0.121492	12	4.05	0.809951	12	0.7%	13.05 [8.94, 17.17]
Liu et al., 2022 (b)	10.93434	0.121492	12	4.05	0.809951	12	0.7%	11.48 [7.84, 15.12]
Kapoor et al 2022 (a)	0.279591	0.076	3	0.05988	0.047	3	0.8%	2.78 [-0.34, 5.91]
Hu et al., 2019 (a)	10.58544	0.237	10	6.97785	0.522	10	0.8%	8.52 [5.45, 11.60]
Hu et al., 2019 (c)	10.1	0.215	10	6.97785	0.522	10	0.9%	7.49 [4.76, 10.23]
Tian et al., 2021 (b)	20.5	3.012	10	7.135	0.321	10	1.0%	5.98 [3.73, 8.22]
Hu et al., 2019 (b)	9.75245	0.412	10	6.97785	0.522	10	1.0%	5.65 [3.51, 7.79]
Tian et al., 2021 (a)	15.7	2.31	10	7.135	0.321	10	1.1%	4.97 [3.04, 6.90]
Tian et al., 2021 (c)	13	1.67	10	7.135	0.321	10	1.1%	4.67 [2.83, 6.51]
Kapoor et al. 2002 (d)	0.123307	0.079	3	0.05988	0.047	3	1.1%	0.78 [-0.99, 2.55]
Kapoor et al. 2022 (c)	0.272885	0.239	3	0.12096	0.085	3	1.1%	0.68 [-1.05, 2.41]
Wu et al., 2018	24.59333	2.021	12	15.8341	1.348	12	1.1%	4.92 [3.20, 6.64]
Kapoor et al. 2022 (b)	0.163493	0.134	3	0.12095	0.085	3	1.1%	0.30 [-1.32, 1.93]
Su et al., 2019	21.65653	1.675	6	17.0973	1.861	6	1.1%	2.38 [0.76, 4.00]
Liu et al., 2022 (a)	9.5844	1.499	12	4.05	0.809951	12	1.1%	4.44 [2.85, 6.02]
Kaur et al., 2022 (a)	38.1	6	4	29.7	8.4	4	1.2%	1.00 [-0.55, 2.55]
Kaur et al., 2022 (b)	35.8	7.4	4	29.7	8.4	4	1.2%	0.67 [-0.79, 2.13]
Silva-maia et al.,2018	121.6931	3.704	7	111.111	4.762	7	1.2%	2.32 [0.86, 3.78]
Wang et al.,2020 (a)	10.15786	0.999	12	6.99394	0.5	12	1.2%	3.87 [2.43, 5.31]
Liu et al., 2020 (b)	15.2518	1.583	6	17.554	2.014	6	1.2%	-1.17 [-2.44, 0.10]
Wang et al.,2020(b)	8.99	0.834	12	6.99394	0.5	12	1.2%	2.80 [1.62, 3.98]
Liu et al., 2020 (a)	17.41007	6.043	6	17.554	2.014	6	1.2%	-0.03 [-1.16, 1.10]
Lee et al., 2018	0.042315	0.007	7	0.03724	0.004	7	1.2%	0.83 [-0.28, 1.94]
da Silva-Maia et al., 2019 (a)	131	4.054	7	134	4.28	7	1.2%	-0.67 [-1.76, 0.41]
Peng et al., 2020	20.69444	1.806	10	16.3889	2.778	10	1.2%	1.76 [0.69, 2.83]
da Silva-Maia et al., 2019 (b)	134	4.154	7	134	4.28	7	1.2%	0.00 [-1.05, 1.05]
Peng et al., 2019 (a)	61.79641	6.707	10	50.3	7.186	10	1.2%	1.58 [0.55, 2.62]
Peng et al., 2019 (b)	57.96407	2.874	10	50.3	7.186	10	1.2%	1.34 [0.35, 2.33]
Wang et al.,2020(c)	7.993	0.66	12	6.99394	0.5	12	1.3%	1.65 [0.70, 2.60]
Morissette et al., 2020	2.868214	1.419	14	3.46082	1.33	14	1.3%	-0.42 [-1.17, 0.33]
**Subtotal (95% CI)**			**254**			**254**	**33.3%**	**2.69 [1.88, 3.50]**
Heterogeneity: Tau² = 4.29; Chi² = 276.89, df = 29 (P < 0.00001); I² = 90%								
Test for overall effect: Z = 6.52 (P < 0.00001)								
**1.1.2 Butanoic Acid**								
Wu et al., 2018	64.75091	3.215	12	19.2875	2.985	12	0.6%	14.15 [9.70, 18.60]
Hu et al., 2019 (c)	1.8235	0.021234	10	2.11628	0.039	10	0.8%	-8.93 [-12.14, -5.72]
Tian et al., 2021 (c)	6.4	0.21	10	2.1	0.622	10	0.8%	8.87 [5.68, 12.06]
Tian et al., 2021 (b)	9	0.92	10	2.1	0.622	10	0.9%	8.42 [5.38, 11.46]
Hu et al., 2019 (a)	2.503876	0.054	10	2.11628	0.039	10	0.9%	7.88 [5.02, 10.74]
Kapoor et al. 2022 (c)	0.586665	0.184	3	0.18935	0.032	3	0.9%	2.41 [-0.41, 5.23]
Kapoor et al 2022 (a)	0.571131	0.119	3	0.35506	0.105	3	1.0%	1.54 [-0.64, 3.73]
Tian et al., 2021 (a)	6	0.821	10	2.1	0.622	10	1.1%	5.13 [3.15, 7.11]
Kapoor et al. 2002 (d)	0.281609	0.057	3	0.35506	0.105	3	1.1%	-0.70 [-2.43, 1.04]
Kapoor et al. 2022 (b)	0.216875	0.121	3	0.18935	0.032	3	1.1%	0.25 [-1.37, 1.87]
da Silva-Maia et al., 2019 (b)	63	1.823	7	77	6.414	7	1.1%	-2.78 [-4.38, -1.18]
Kaur et al., 2022 (b)	1.21	0.51	4	0.71	0.32	4	1.2%	1.02 [-0.53, 2.58]
da Silva-Maia et al., 2019 (a)	65	2.534	7	77	6.414	7	1.2%	-2.30 [-3.76, -0.85]
Hu et al., 2019 (b)	2.2215	0.021569	10	2.11628	0.039	10	1.2%	3.20 [1.79, 4.61]
Kaur et al., 2022 (a)	0.69	0.16	4	0.71	0.32	4	1.2%	-0.07 [-1.46, 1.32]
Wang et al.,2020(b)	9.6583	0.83261	12	5.828286	1.1656	12	1.2%	3.65 [2.27, 5.04]
Wang et al.,2020 (a)	10.65743	1.4987	12	5.828286	1.1656	12	1.2%	3.47 [2.13, 4.81]
Liu et al., 2022 (b)	12.82423	2.5648	12	6.15562	0.944943	12	1.2%	3.33 [2.03, 4.64]
Liu et al., 2020 (a)	4.163424	2.412	6	2.56809	0.584	6	1.2%	0.84 [-0.36, 2.04]
Silva-maia et al.,2018	30.30303	3.207	7	37.5758	6.414	7	1.2%	-1.34 [-2.54, -0.14]
Liu et al., 2022 (a)	8.63947	0.80995	12	6.15562	0.944943	12	1.2%	2.73 [1.56, 3.89]
Su et al., 2019	14.31818	6.309	6	11.7424	6.309	6	1.2%	0.38 [-0.77, 1.52]
Liu et al., 2020 (b)	2.918288	1.323	6	2.56809	0.584	6	1.2%	0.32 [-0.83, 1.46]
Peng et al., 2020	20.45455	3.846	10	13.6364	4.196	10	1.2%	1.62 [0.58, 2.66]
Liu et al., 2022 (c)	13.90416	5.39	12	6.15562	0.944943	12	1.2%	1.93 [0.93, 2.93]
Lee et al., 2018	0.011497	0.002	8	0.01217	0.004	8	1.2%	-0.20 [-1.18, 0.78]
Peng et al., 2019 (b)	14.37126	2.395	10	11.018	3.353	10	1.3%	1.10 [0.15, 2.06]
Peng et al., 2019 (a)	14.8503	4.79	10	11.018	3.353	10	1.3%	0.89 [-0.04, 1.82]
Wang et al.,2020(c)	6.660898	1.16565	12	5.828286	1.1656	12	1.3%	0.69 [-0.14, 1.52]
Morissette et al., 2020	1.884899	0.177	14	1.83817	0.266	14	1.3%	0.20 [-0.54, 0.94]
**Subtotal (95% CI)**			**255**			**255**	**33.5%**	**1.60 [0.82, 2.39]**
Heterogeneity: Tau² = 4.07; Chi² = 294.29, df = 29 (P < 0.00001); I² = 90%								
Test for overall effect: Z = 3.99 (P < 0.0001)								
**1.1.3 Propanoic Acid**								
Liu et al., 2022 (c)	11.74429	1.079934	12	2.15987	0.27	12	0.7%	11.76 [8.03, 15.48]
Tian et al., 2021 (c)	8.5	0.921	10	1.8	0.246	10	0.8%	9.52 [6.11, 12.93]
Tian et al., 2021 (b)	12	1.56	10	1.8	0.246	10	0.8%	8.75 [5.60, 11.90]
Liu et al., 2022 (b)	7.559544	0.809951	12	2.15987	0.27	12	0.9%	8.64 [5.85, 11.43]
Kapoor et al. 2022 (c)	0.303184	0.134	3	0.03512	0.002	3	0.9%	2.26 [-0.45, 4.97]
Kapoor et al. 2002 (d)	0.029368	0.01	3	0.00979	0.007	3	1.0%	1.81 [-0.56, 4.19]
Wang et al.,2020(b)	5.9396	0.5399	12	2.15983	0.54	12	1.0%	6.76 [4.52, 9.00]
Liu et al., 2022 (a)	5.6696	0.674959	12	2.15987	0.27	12	1.0%	6.59 [4.40, 8.78]
da Silva-Maia et al., 2019 (a)	22.31313	0.256	7	26	1.138	7	1.0%	-4.18 [-6.29, -2.08]
Kapoor et al 2022 (a)	0.17168	0.135	3	0.00979	0.007	3	1.1%	1.35 [-0.71, 3.42]
Wang et al.,2020 (a)	7.424406	1.08	12	2.15983	0.54	12	1.1%	5.95 [3.94, 7.96]
Tian et al., 2021 (a)	8.26	1.741	10	1.8	0.246	10	1.1%	4.98 [3.04, 6.91]
Kapoor et al. 2022 (b)	0.094367	0.071	3	0.03512	0.002	3	1.1%	0.94 [-0.90, 2.79]
Hu et al., 2019 (a)	6.098765	0.395	10	4.54321	0.321	10	1.1%	4.14 [2.46, 5.82]
Hu et al., 2019 (b)	6.1	0.4253	10	4.54321	0.321	10	1.1%	3.96 [2.33, 5.58]
Hu et al., 2019 (c)	6.2	0.5214	10	4.54321	0.321	10	1.2%	3.67 [2.12, 5.21]
Kaur et al., 2022 (a)	1.7	0.38	4	2.2	0.88	4	1.2%	-0.64 [-2.10, 0.81]
Kaur et al., 2022 (b)	2.4	0.66	4	2.2	0.88	4	1.2%	0.22 [-1.17, 1.62]
Liu et al., 2020 (a)	3.923954	0.669	6	4.92776	0.578	6	1.2%	-1.48 [-2.83, -0.14]
Su et al., 2019	18.10606	2.598	6	13.1061	3.711	6	1.2%	1.44 [0.11, 2.77]
Liu et al., 2020 (b)	4.228137	0.943	6	4.92776	0.578	6	1.2%	-0.83 [-2.03, 0.38]
Wang et al.,2020(c)	3.914	0.6749	12	2.15983	0.54	12	1.2%	2.77 [1.60, 3.94]
da Silva-Maia et al., 2019 (b)	25.123	0.623	7	26	1.138	7	1.2%	-0.89 [-2.01, 0.22]
Silva-maia et al.,2018	8.695652	3.704	7	10.4348	4.762	7	1.2%	-0.38 [-1.44, 0.68]
Lee et al., 2018	0.281015	0.037	8	0.23958	0.051	8	1.2%	0.88 [-0.16, 1.92]
Peng et al., 2019 (a)	14.37126	1.916	10	10.5389	3.832	10	1.2%	1.21 [0.24, 2.18]
Peng et al., 2019 (b)	11.97605	1.437	10	10.5389	3.832	10	1.3%	0.48 [-0.42, 1.37]
Wu et al., 2018	15.02047	1.092	12	12.5626	2.458	12	1.3%	1.25 [0.36, 2.14]
Peng et al., 2020	3.055556	0.833	10	3.19444	0.694	10	1.3%	-0.17 [-1.05, 0.71]
Morissette et al., 2020	0.592058	0.177	14	0.73889	0.266	14	1.3%	-0.63 [-1.39, 0.13]
**Subtotal (95% CI)**			**255**			**255**	**33.2%**	**2.33 [1.45, 3.22]**
Heterogeneity: Tau² = 5.28; Chi² = 341.42, df = 29 (P < 0.00001); I² = 92%								
Test for overall effect: Z = 5.15 (P < 0.00001)								
**Total (95% CI)**			**764**			**764**	**100.0%**	**2.20 [1.73, 2.67]**
Heterogeneity: Tau² = 4.41; Chi² = 922.22, df = 89 (P < 0.00001); I² = 90%								
Test for overall effect: Z = 9.13 (P < 0.00001)								
Test for subgroup differences: Chi² = 3.71, df = 2 (P = 0.16), I² = 46.1%								

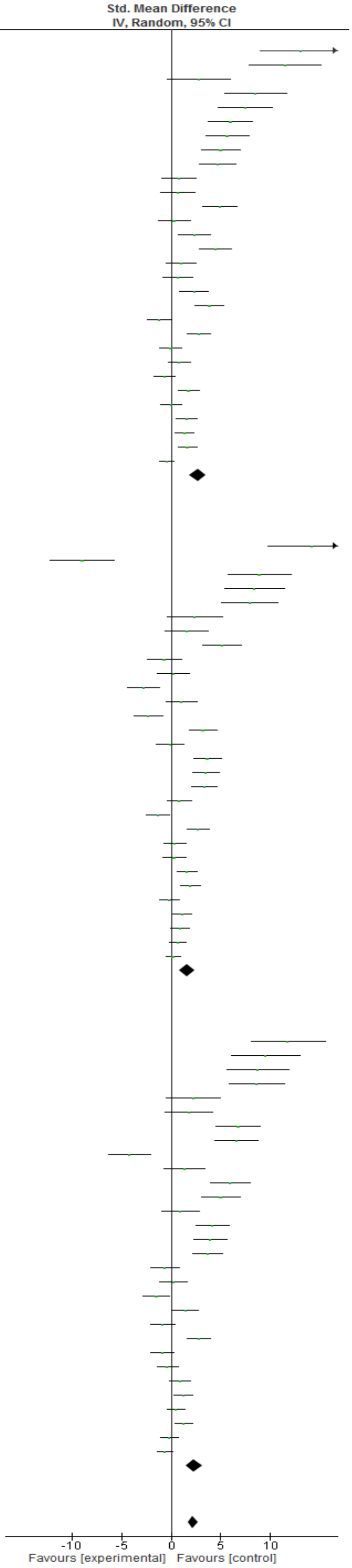

S Figure 5: Forest plot of studies investigating the sub-grouped based effect of anthocyanin supplementation on the SCFA profile. Sub-grouping was on the basis of proportion of acetic acid, butanoic acid and propionic acid. Pooled effect estimates are shown by diamonds. Values are standardized mean differences with 95% CIs determined with the use of random-effects models. Heterogeneity was quantified by I^2, inverse variance and standardised mean difference (SMD).

Study or Subgroup	Experimental Mean	SD	Total	Control Mean	SD	Total	Weight	Std. Mean Difference IV, Random, 95% CI
**1.1.1 Study less than 4 weeks**								
Hu et al., 2019 (a)	10.58544304	0.237	10	6.97785	0.522	10	1.0%	8.52 [5.45, 11.60]
Hu et al., 2019 (b)	9.75245	0.412	10	6.97785	0.522	10	1.2%	5.65 [3.51, 7.79]
Hu et al., 2019 (c)	10.1	0.215	10	6.97785	0.522	10	1.1%	7.49 [4.76, 10.23]
Liu et al., 2020 (a)	17.41007194	6.043	6	17.554	2.014	6	1.5%	-0.03 [-1.16, 1.10]
Liu et al., 2020 (b)	15.25179856	1.583	6	17.554	2.014	6	1.4%	-1.17 [-2.44, 0.10]
Peng et al., 2019 (a)	61.79640719	6.707	10	50.3	7.186	10	1.5%	1.58 [0.55, 2.62]
Peng et al., 2019 (b)	57.96407186	2.874	10	50.3	7.186	10	1.5%	1.34 [0.35, 2.33]
**Subtotal (95% CI)**			**62**			**62**	**9.2%**	**2.97 [1.01, 4.93]**
Heterogeneity: Tau² = 6.09; Chi² = 76.87, df = 6 (P < 0.00001); I² = 92%								
Test for overall effect: Z = 2.98 (P = 0.003)								
**1.1.2 Study equal to or more than 4 weeks**								
da Silva-Maia et al., 2019 (a)	131	4.054	7	134	4.28	7	1.5%	-0.67 [-1.76, 0.41]
da Silva-Maia et al., 2019 (b)	134	4.154	7	134	4.28	7	1.5%	0.00 [-1.05, 1.05]
Kapoor et al.,2022 (a)	0.279591	0.076	3	0.05988	0.047	3	1.0%	2.78 [-0.34, 5.91]
Kapoor et al.,2022 (b)	0.163493	0.134	3	0.12095	0.085	3	1.4%	0.30 [-1.32, 1.93]
Kapoor et al., 2022 (c)	0.272885	0.239	3	0.12096	0.085	3	1.3%	0.68 [-1.05, 2.41]
Kapoor et al., 2022 (d)	0.123307	0.079	3	0.05988	0.047	3	1.3%	0.78 [-0.99, 2.55]
Kaur et al., 2022 (a)	38.1	6	4	29.7	8.4	4	1.4%	1.00 [-0.55, 2.55]
Kaur et al., 2022 (b)	35.8	7.4	4	29.7	8.4	4	1.4%	0.67 [-0.79, 2.13]
Lee et al., 2018	0.042315	0.007	8	0.03724	0.004	8	1.5%	0.84 [-0.19, 1.88]
Liu et al., 2022 (a)	9.5844	1.499	12	4.05	0.809951	12	1.4%	4.44 [2.85, 6.02]
Liu et al., 2022 (b)	10.93434	0.121492	12	4.05	0.809951	12	0.9%	11.48 [7.84, 15.12]
Liu et al., 2022 (c)	11.8792	0.121492	12	4.05	0.809951	12	0.8%	13.05 [8.94, 17.17]
Morissette et al., 2020	2.868214	1.419	14	3.46082	1.33	14	1.5%	-0.42 [-1.17, 0.33]
Peng et al., 2020	20.694	1.806	10	16.3889	2.778	10	1.5%	1.76 [0.69, 2.83]
Silva-maia et al., 2018	121.6931	3.704	7	111.111	4.762	7	1.4%	2.32 [0.86, 3.78]
Su et al., 2019	21.65653	1.675	6	17.0973	1.861	6	1.4%	2.38 [0.76, 4.00]
Tian et al., 2021 (a)	15.7	2.31	10	7.135	0.321	10	1.3%	4.97 [3.04, 6.90]
Tian et al., 2021 (b)	20.5	3.012	10	7.135	0.321	10	1.2%	5.98 [3.73, 8.22]
Tian et al., 2021 (c)	13	1.67	10	7.135	0.321	10	1.3%	4.67 [2.83, 6.51]
Wang et al.,2020(a)	10.15786	0.999	12	6.99394	0.5	12	1.4%	3.87 [2.43, 5.31]
Wang et al.,2020(b)	8.99	0.834	12	6.99394	0.5	12	1.5%	2.80 [1.62, 3.98]
Wang et al.,2020(c)	7.993	0.66	12	6.99394	0.5	12	1.5%	1.65 [0.70, 2.60]
Wu et al., 2018	24.59333	2.021	12	15.8341	1.348	12	1.3%	4.92 [3.20, 6.64]
**Subtotal (95% CI)**			**193**			**193**	**30.7%**	**2.63 [1.73, 3.54]**
Heterogeneity: Tau² = 4.08; Chi² = 199.69, df = 22 (P < 0.00001); I² = 89%								
Test for overall effect: Z = 5.71 (P < 0.00001)								
**1.1.3 Lower Dose**								
Hu et al., 2019 (a)	10.1	0.215	10	6.97785	0.522	10	1.1%	7.49 [4.76, 10.23]
Kapoor et al.,2022 (a)	0.279591	0.076	3	0.05988	0.047	3	1.0%	2.78 [-0.34, 5.91]
Kapoor et al., 2022 (c)	0.123307	0.079	3	0.05988	0.047	3	1.3%	0.78 [-0.99, 2.55]
Kaur et al., 2022 (a)	35.8	7.4	4	29.7	8.4	4	1.4%	0.67 [-0.79, 2.13]
Liu et al., 2020 (a)	17.41007	6.043	6	17.554	2.014	6	1.5%	-0.03 [-1.16, 1.10]
Liu et al., 2022 (a)	9.5844	1.499	12	4.05	0.809951	12	1.4%	4.44 [2.85, 6.02]
Tian et al., 2021 (a)	15.7	2.31	10	7.135	0.321	10	1.3%	4.97 [3.04, 6.90]
Wang et al.,2020(a)	7.993	0.66	12	6.99394	0.5	12	1.5%	1.65 [0.70, 2.60]
**Subtotal (95% CI)**			**60**			**60**	**10.5%**	**2.65 [1.12, 4.19]**
Heterogeneity: Tau² = 4.00; Chi² = 51.58, df = 7 (P < 0.00001); I² = 86%								
Test for overall effect: Z = 3.39 (P = 0.0007)								
**1.1.4 Higher Dose**								
Hu et al., 2019 (c)	10.58544	0.237	10	6.97785	0.522	10	1.0%	8.52 [5.45, 11.60]
Kapoor et al.,2022 (b)	0.272885	0.239	3	0.12096	0.085	3	1.3%	0.68 [-1.05, 2.41]
Kapoor et al., 2022 (d)	0.279591	0.076	3	0.05988	0.047	3	1.0%	2.78 [-0.34, 5.91]
Kaur et al., 2022 (b)	38.1	6	4	29.7	8.4	4	1.4%	1.00 [-0.55, 2.55]
Liu et al., 2020 (b)	15.2518	1.583	6	17.554	2.014	6	1.4%	-1.17 [-2.44, 0.10]
Liu et al., 2022 (c)	11.8792	0.121492	12	4.05	0.809951	12	0.8%	13.05 [8.94, 17.17]
Tian et al., 2021 (c)	13	1.67	10	7.135	0.321	10	1.3%	4.67 [2.83, 6.51]
Wang et al.,2020(c)	10.15786	0.999	12	6.99394	0.5	12	1.4%	3.87 [2.43, 5.31]
**Subtotal (95% CI)**			**60**			**60**	**9.7%**	**3.81 [1.41, 6.20]**
Heterogeneity: Tau² = 10.49; Chi² = 87.56, df = 7 (P < 0.00001); I² = 92%								
Test for overall effect: Z = 3.12 (P = 0.002)								
**1.1.5 High Fat/cholesterol Model**								
Lee et al., 2018	0.04231523	0.007	8	0.03724	0.004	8	1.5%	0.84 [-0.19, 1.88]
Liu et al., 2022 (a)	9.5844	1.499	12	4.05	0.809951	12	1.4%	4.44 [2.85, 6.02]
Liu et al., 2022 (b)	10.93434	0.121492	12	4.05	0.809951	12	0.9%	11.48 [7.84, 15.12]
Liu et al., 2022 (c)	11.8792	0.121492	12	4.05	0.809951	12	0.8%	13.05 [8.94, 17.17]
Morissette et al., 2020	2.8682141	1.419	14	3.46082	1.33	14	1.5%	-0.42 [-1.17, 0.33]
Tian et al., 2021 (a)	15.7	2.31	10	7.135	0.321	10	1.3%	4.97 [3.04, 6.90]
Tian et al., 2021 (b)	20.5	3.012	10	7.135	0.321	10	1.2%	5.98 [3.73, 8.22]
Tian et al., 2021 (c)	13	1.67	10	7.135	0.321	10	1.3%	4.67 [2.83, 6.51]
Wang et al.,2020(a)	10.15786	0.999	12	6.99394	0.5	12	1.4%	3.87 [2.43, 5.31]
Wang et al.,2020(b)	8.99	0.834	12	6.99394	0.5	12	1.5%	2.80 [1.62, 3.98]
Wang et al.,2020(c)	7.993	0.66	12	6.99394	0.5	12	1.5%	1.65 [0.70, 2.60]
Wu et al., 2018	24.59332933	2.021	12	15.8341	1.348	12	1.3%	4.92 [3.20, 6.64]
**Subtotal (95% CI)**			**136**			**136**	**15.6%**	**4.36 [2.80, 5.92]**
Heterogeneity: Tau² = 6.63; Chi² = 155.47, df = 11 (P < 0.00001); I² = 93%								
Test for overall effect: Z = 5.47 (P < 0.00001)								
**1.1.6 Other Animal Models**								
da Silva-Maia et al., 2019 (a)	131	4.054	7	134	4.28	7	1.5%	-0.67 [-1.76, 0.41]
da Silva-Maia et al., 2019 (b)	134	4.154	7	134	4.28	7	1.5%	0.00 [-1.05, 1.05]
Hu et al., 2019 (a)	10.58544	0.237	10	6.97785	0.522	10	1.0%	8.52 [5.45, 11.60]
Hu et al., 2019 (b)	9.75245	0.412	10	6.97785	0.522	10	1.2%	5.65 [3.51, 7.79]
Hu et al., 2019 (c)	10.1	0.215	10	6.97785	0.522	10	1.1%	7.49 [4.76, 10.23]
Kapoor et al.,2022 (a)	0.163493	0.134	3	0.12095	0.085	3	1.4%	0.30 [-1.32, 1.93]
Kapoor et al.,2022 (b)	0.272885	0.239	3	0.12096	0.085	3	1.3%	0.68 [-1.05, 2.41]
Kapoor et al., 2022 (c)	0.123307	0.079	3	0.05988	0.047	3	1.3%	0.78 [-0.99, 2.55]
Kapoor et al., 2022 (d)	0.279591	0.076	3	0.05988	0.047	3	1.0%	2.78 [-0.34, 5.91]
Kaur et al., 2022 (a)	38.1	6	4	29.7	8.4	4	1.4%	1.00 [-0.55, 2.55]
Kaur et al., 2022 (b)	35.8	7.4	4	29.7	8.4	4	1.4%	0.67 [-0.79, 2.13]
Liu et al., 2020 (a)	17.41007	6.043	6	17.554	2.014	6	1.5%	-0.03 [-1.16, 1.10]
Liu et al., 2020 (b)	15.2518	1.583	6	17.554	2.014	6	1.4%	-1.17 [-2.44, 0.10]
Peng et al., 2019 (a)	61.79641	6.707	10	50.3	7.186	10	1.5%	1.58 [0.55, 2.62]
Peng et al., 2019 (b)	57.96407	2.874	10	50.3	7.186	10	1.5%	1.34 [0.35, 2.33]
Peng et al., 2020	20.694	1.806	10	16.3889	2.778	10	1.5%	1.76 [0.69, 2.83]
Silva-maia et al., 2018	121.6931	3.704	7	111.111	4.762	7	1.4%	2.32 [0.86, 3.78]
Su et al., 2019	21.65653	1.675	6	17.0973	1.861	6	1.4%	2.38 [0.76, 4.00]
**Subtotal (95% CI)**			**119**			**119**	**24.3%**	**1.63 [0.80, 2.47]**
Heterogeneity: Tau² = 2.55; Chi² = 101.59, df = 17 (P < 0.00001); I² = 83%								
Test for overall effect: Z = 3.83 (P = 0.0001)								
**Total (95% CI)**			**630**			**630**	**100.0%**	**2.77 [2.26, 3.29]**
Heterogeneity: Tau² = 4.36; Chi² = 697.39, df = 75 (P < 0.00001); I² = 89%								
Test for overall effect: Z = 10.55 (P < 0.00001)								
Test for subgroup differences: Chi² = 11.10, df = 5 (P = 0.05), I² = 55.0%								

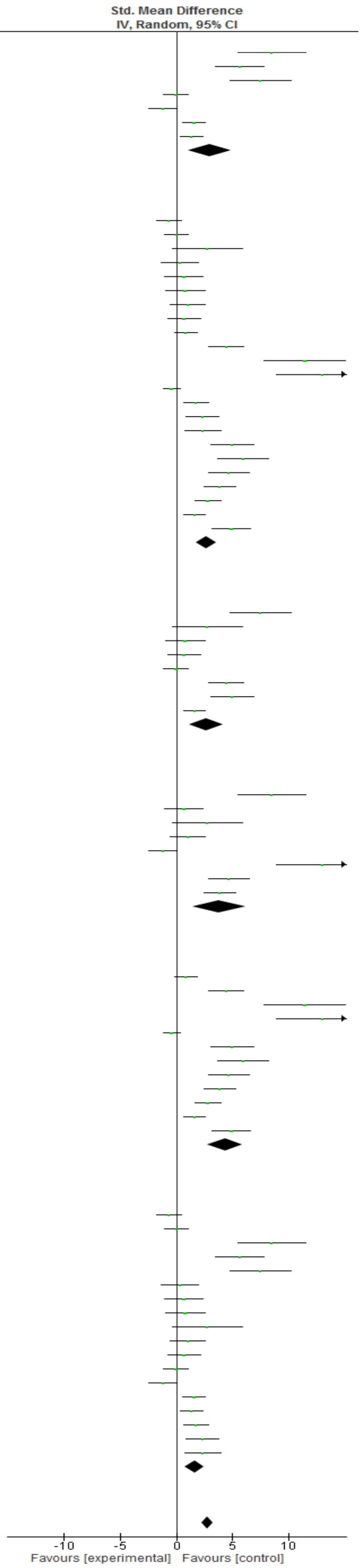

S Figure 6: Forest plot of the sub-group analysis to study effect of anthocyanin supplementation on the acetic acid, SCFA. Sub-grouping was on the basis of duration of the study, dose of the anthocyanin given and disease status of the subjects (animal models). Values are standardized mean differences with 95% CIs determined with the use of random-effects models. Heterogeneity was quantified by I^2, inverse variance and standardised mean difference (SMD).

Study or Subgroup	Experimental Mean	Experimental SD	Experimental Total	Control Mean	Control SD	Control Total	Weight	Std. Mean Difference IV, Random, 95% CI
**1.1.1 Study less than 4 weeks**								
Liu et al., 2020 (a)	17.41007194	6.043	6	17.554	2.014	6	2.0%	-0.03 [-1.16, 1.10]
Liu et al., 2020 (b)	15.25179856	1.583	6	17.554	2.014	6	1.9%	-1.17 [-2.44, 0.10]
Peng et al., 2019 (a)	61.79640719	6.707	10	50.3	7.186	10	2.0%	1.58 [0.55, 2.62]
Peng et al., 2019 (b)	57.96407186	2.874	10	50.3	7.186	10	2.0%	1.34 [0.35, 2.33]
**Subtotal (95% CI)**			**32**			**32**	**8.0%**	**0.47 [-0.73, 1.68]**
Heterogeneity: Tau² = 1.18; Chi² = 14.32, df = 3 (P = 0.003); I² = 79%								
Test for overall effect: Z = 0.77 (P = 0.44)								
**1.1.2 Study equal to or more than 4 weeks**								
da Silva-Maia et al., 2019 (a)	131	4.054	7	134	4.28	7	2.0%	-0.67 [-1.76, 0.41]
da Silva-Maia et al., 2019 (b)	134	4.154	7	134	4.28	7	2.0%	0.00 [-1.05, 1.05]
Kapoor et al.,2022 (a)	0.279591	0.076	3	0.05988	0.047	3	1.0%	2.78 [-0.34, 5.91]
Kapoor et al.,2022 (b)	0.163493	0.134	3	0.12095	0.085	3	1.7%	0.30 [-1.32, 1.93]
Kapoor et al., 2022 (c)	0.272885	0.239	3	0.12096	0.085	3	1.7%	0.68 [-1.05, 2.41]
Kapoor et al., 2022 (d)	0.123307	0.079	3	0.05988	0.047	3	1.6%	0.78 [-0.99, 2.55]
Kaur et al., 2022 (a)	38.1	6	4	29.7	8.4	4	1.8%	1.00 [-0.55, 2.55]
Kaur et al., 2022 (b)	35.8	7.4	4	29.7	8.4	4	1.8%	0.67 [-0.79, 2.13]
Lee et al., 2018	0.042315	0.007	8	0.03724	0.004	8	2.0%	0.84 [-0.19, 1.88]
Morissette et al., 2020	2.868214	1.419	14	3.46082	1.33	14	2.2%	-0.42 [-1.17, 0.33]
Peng et al., 2020	20.694	1.806	10	16.3889	2.778	10	2.0%	1.76 [0.69, 2.83]
Silva-maia et al., 2018	121.6931	3.704	7	111.111	4.762	7	1.8%	2.32 [0.86, 3.78]
Su et al., 2019	21.65653	1.675	6	17.0973	1.861	6	1.7%	2.38 [0.76, 4.00]
Tian et al., 2021 (a)	15.7	2.31	10	7.135	0.321	10	1.6%	4.97 [3.04, 6.90]
Tian et al., 2021 (b)	20.5	3.012	10	7.135	0.321	10	1.4%	5.98 [3.73, 8.22]
Tian et al., 2021 (c)	13	1.67	10	7.135	0.321	10	1.6%	4.67 [2.83, 6.51]
Wang et al.,2020(a)	10.15786	0.999	12	6.99394	0.5	12	1.8%	3.87 [2.43, 5.31]
Wang et al.,2020(b)	8.99	0.834	12	6.99394	0.5	12	2.0%	2.80 [1.62, 3.98]
Wang et al.,2020(c)	7.993	0.66	12	6.99394	0.5	12	2.1%	1.65 [0.70, 2.60]
**Subtotal (95% CI)**			**145**			**145**	**33.7%**	**1.78 [1.01, 2.54]**
Heterogeneity: Tau² = 2.30; Chi² = 110.15, df = 18 (P < 0.00001); I² = 84%								
Test for overall effect: Z = 4.55 (P < 0.00001)								
**1.1.3 Lower Dose**								
Kapoor et al.,2022 (a)	0.1634931	0.076	3	0.12096	0.047	3	1.7%	0.54 [-1.14, 2.22]
Kapoor et al., 2022 (c)	0.123307	0.079	3	0.05988	0.047	3	1.6%	0.78 [-0.99, 2.55]
Kaur et al., 2022 (a)	35.8	7.4	4	29.7	8.4	4	1.8%	0.67 [-0.79, 2.13]
Tian et al., 2021 (a)	15.7	2.31	10	7.135	0.321	10	1.6%	4.97 [3.04, 6.90]
Wang et al.,2020(a)	7.993	0.66	12	6.99394	0.5	12	2.1%	1.65 [0.70, 2.60]
**Subtotal (95% CI)**			**32**			**32**	**8.8%**	**1.65 [0.33, 2.98]**
Heterogeneity: Tau² = 1.66; Chi² = 15.63, df = 4 (P = 0.004); I² = 74%								
Test for overall effect: Z = 2.44 (P = 0.01)								
**1.1.4 Higher Dose**								
Kapoor et al.,2022 (b)	0.272885	0.239	3	0.12096	0.085	3	1.7%	0.68 [-1.05, 2.41]
Kapoor et al., 2022 (d)	0.279591	0.076	3	0.05988	0.047	3	1.0%	2.78 [-0.34, 5.91]
Kaur et al., 2022 (b)	38.1	6	4	29.7	8.4	4	1.8%	1.00 [-0.55, 2.55]
Tian et al., 2021 (c)	13	1.67	10	7.135	0.321	10	1.6%	4.67 [2.83, 6.51]
Wang et al.,2020(c)	10.15786	0.999	12	6.99394	0.5	12	1.8%	3.87 [2.43, 5.31]
**Subtotal (95% CI)**			**32**			**32**	**7.9%**	**2.58 [0.92, 4.24]**
Heterogeneity: Tau² = 2.64; Chi² = 16.71, df = 4 (P = 0.002); I² = 76%								
Test for overall effect: Z = 3.04 (P = 0.002)								
**1.1.5 High Fat/cholesterol Model**								
Lee et al., 2018	0.04231523	0.007	8	0.03724	0.004	8	2.0%	0.84 [-0.19, 1.88]
Morissette et al., 2020	2.8682141	1.419	14	3.46082	1.33	14	2.2%	-0.42 [-1.17, 0.33]
Tian et al., 2021 (a)	15.7	2.31	10	7.135	0.321	10	1.6%	4.97 [3.04, 6.90]
Tian et al., 2021 (b)	20.5	3.012	10	7.135	0.321	10	1.4%	5.98 [3.73, 8.22]
Tian et al., 2021 (c)	13	1.67	10	7.135	0.321	10	1.6%	4.67 [2.83, 6.51]
Wang et al.,2020(a)	10.15786	0.999	12	6.99394	0.5	12	1.8%	3.87 [2.43, 5.31]
Wang et al.,2020(b)	8.99	0.834	12	6.99394	0.5	12	2.0%	2.80 [1.62, 3.98]
Wang et al.,2020(c)	7.993	0.66	12	6.99394	0.5	12	2.1%	1.65 [0.70, 2.60]
**Subtotal (95% CI)**			**88**			**88**	**14.6%**	**2.89 [1.40, 4.37]**
Heterogeneity: Tau² = 4.05; Chi² = 80.35, df = 7 (P < 0.00001); I² = 91%								
Test for overall effect: Z = 3.80 (P = 0.0001)								
**1.1.6 Other Animal Models**								
da Silva-Maia et al., 2019 (a)	131	4.054	7	134	4.28	7	2.0%	-0.67 [-1.76, 0.41]
da Silva-Maia et al., 2019 (b)	134	4.154	7	134	4.28	7	2.0%	0.00 [-1.05, 1.05]
Kapoor et al.,2022 (a)	0.163493	0.134	3	0.12095	0.085	3	1.7%	0.30 [-1.32, 1.93]
Kapoor et al.,2022 (b)	0.272885	0.239	3	0.12096	0.085	3	1.7%	0.68 [-1.05, 2.41]
Kapoor et al., 2022 (c)	0.123307	0.079	3	0.05988	0.047	3	1.6%	0.78 [-0.99, 2.55]
Kapoor et al., 2022 (d)	0.279591	0.076	3	0.05988	0.047	3	1.0%	2.78 [-0.34, 5.91]
Kaur et al., 2022 (a)	38.1	6	4	29.7	8.4	4	1.8%	1.00 [-0.55, 2.55]
Kaur et al., 2022 (b)	35.8	7.4	4	29.7	8.4	4	1.8%	0.67 [-0.79, 2.13]
Liu et al., 2020 (a)	17.41007	6.043	6	17.554	2.014	6	2.0%	-0.03 [-1.16, 1.10]
Liu et al., 2020 (b)	15.2518	1.583	6	17.554	2.014	6	1.9%	-1.17 [-2.44, 0.10]
Peng et al., 2019 (a)	61.79641	6.707	10	50.3	7.186	10	2.0%	1.58 [0.55, 2.62]
Peng et al., 2019 (b)	57.96407	2.874	10	50.3	7.186	10	2.0%	1.34 [0.35, 2.33]
Peng et al., 2020	20.694	1.806	10	16.3889	2.778	10	2.0%	1.76 [0.69, 2.83]
Silva-maia et al., 2018	121.6931	3.704	7	111.111	4.762	7	1.8%	2.32 [0.86, 3.78]
Su et al., 2019	21.65653	1.675	6	17.0973	1.861	6	1.7%	2.38 [0.76, 4.00]
**Subtotal (95% CI)**			**89**			**89**	**27.1%**	**0.81 [0.24, 1.37]**
Heterogeneity: Tau² = 0.73; Chi² = 36.87, df = 14 (P = 0.0008); I² = 62%								
Test for overall effect: Z = 2.80 (P = 0.005)								
**Total (95% CI)**			**418**			**418**	**100.0%**	**1.61 [1.19, 2.03]**
Heterogeneity: Tau² = 1.98; Chi² = 302.91, df = 55 (P < 0.00001); I² = 82%								
Test for overall effect: Z = 7.52 (P < 0.00001)								
Test for subgroup differences: Chi² = 13.16, df = 5 (P = 0.02), I² = 62.0%								

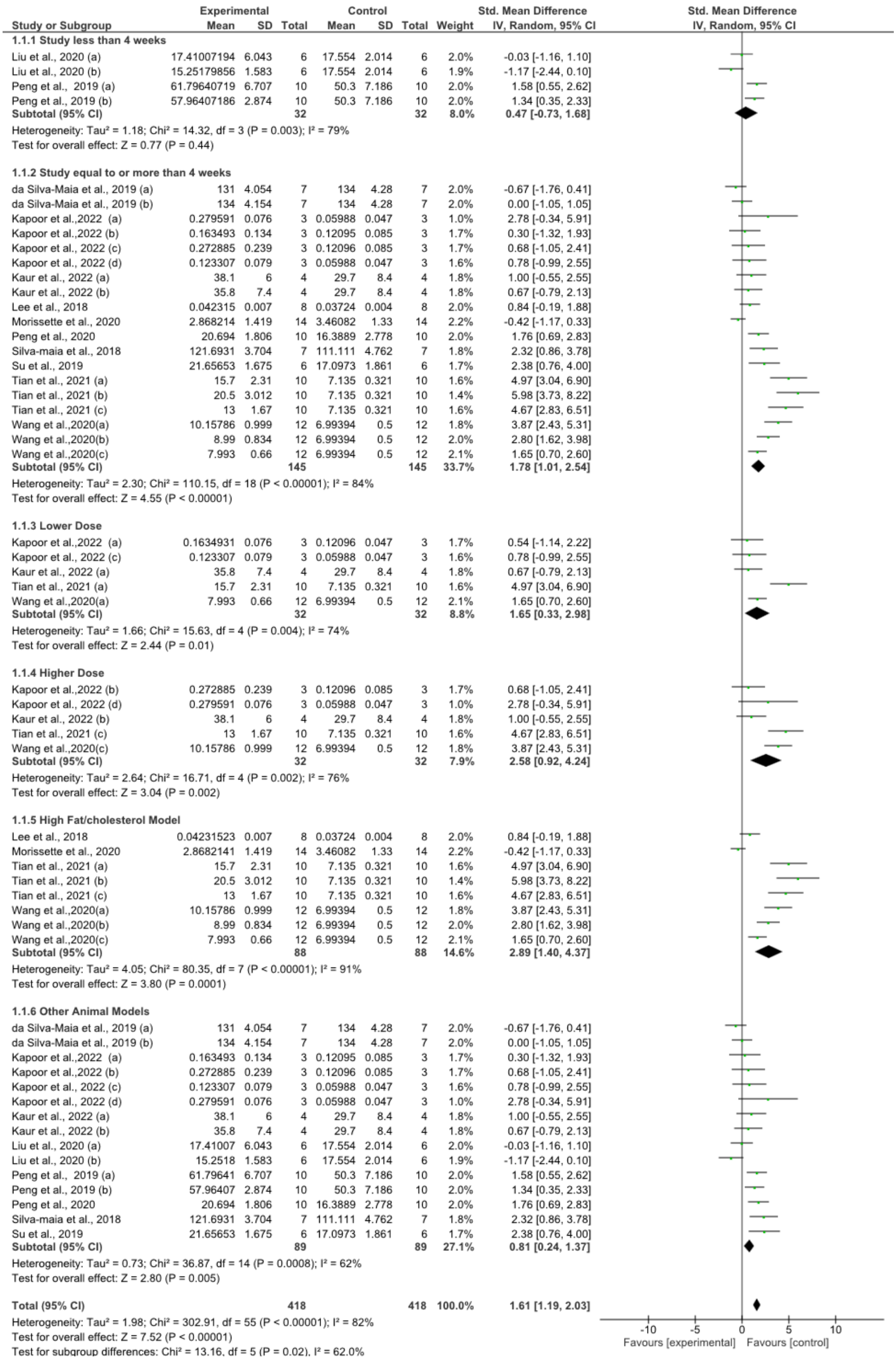

S Figure 7: Forest plot of the sub-group analysis after elimination of high-influencer studies, for understanding the effect of anthocyanin supplementation on the acetic acid, SCFA type. Sub-grouping was on the basis of duration of the study, dose of the anthocyanin given and disease status of the subjects (animal models). Values are standardized mean differences with 95% CIs determined with the use of random-effects models. Heterogeneity was quantified by I^2, inverse variance and standardised mean difference (SMD).

	Experimental			Control				Std. Mean Difference
**Study or Subgroup**	**Mean**	**SD**	**Total**	**Mean**	**SD**	**Total**	**Weight**	**IV, Random, 95% CI**
**3.1.1 Study less than 4 weeks**								
Hu et al., 2019 (a)	2.503876	0.054	10	2.11628	0.039	10	1.0%	7.88 [5.02, 10.74]
Hu et al., 2019 (b)	2.2215	0.021569	10	2.11628	0.039	10	1.4%	3.20 [1.79, 4.61]
Hu et al., 2019 (c)	1.8235	0.021234	10	2.11628	0.039	10	0.9%	-8.93 [-12.14, -5.72]
Liu et al., 2020 (a)	4.163424	2.412	6	2.56809	0.584	6	1.4%	0.84 [-0.36, 2.04]
Liu et al., 2020 (b)	2.918288	1.323	6	2.56809	0.584	6	1.5%	0.32 [-0.83, 1.46]
Peng et al., 2019 (a)	14.8503	4.79	10	11.018	3.353	10	1.5%	0.89 [-0.04, 1.82]
Peng et al., 2019 (b)	14.37126	2.395	10	11.018	3.353	10	1.5%	1.10 [0.15, 2.06]
**Subtotal (95% CI)**			**62**			**62**	**9.3%**	**0.93 [-0.80, 2.66]**
Heterogeneity: Tau² = 4.66; Chi² = 69.72, df = 6 (P < 0.00001); I² = 91%								
Test for overall effect: Z = 1.06 (P = 0.29)								
**3.1.2 Study more than 4 weeks**								
da Silva-Maia et al., 2019 (a)	65	2.534	7	77	6.414	7	1.4%	-2.30 [-3.76, -0.85]
da Silva-Maia et al., 2019 (b)	63	1.823	7	77	6.414	7	1.4%	-2.78 [-4.38, -1.18]
Kapoor et al.,2022 (a)	0.571131	0.119	3	0.35506	0.105	3	1.2%	1.54 [-0.64, 3.73]
Kapoor et al.,2022 (b)	0.216875	0.121	3	0.18935	0.032	3	1.4%	0.25 [-1.37, 1.87]
Kapoor et al., 2022 (c)	0.58665	0.184	3	0.18935	0.032	3	1.0%	2.41 [-0.41, 5.23]
Kapoor et al., 2022 (d)	0.281609	0.057	3	0.35506	0.105	3	1.3%	-0.70 [-2.43, 1.04]
Kaur et al., 2022 (a)	0.69	0.16	4	0.71	0.32	4	1.4%	-0.07 [-1.46, 1.32]
Kaur et al., 2022 (b)	1.21	0.51	4	0.71	0.32	4	1.4%	1.02 [-0.53, 2.58]
Lee et al., 2018	0.011497	0.002	8	0.01217	0.004	8	1.5%	-0.20 [-1.18, 0.78]
Liu et al., 2022 (a)	8.63947	0.80995	12	6.15562	0.944943	12	1.5%	2.73 [1.56, 3.89]
Liu et al., 2022 (b)	12.82423	2.5648	12	6.15562	0.944943	12	1.4%	3.33 [2.03, 4.64]
Liu et al., 2022 (c)	13.90416	5.39	12	6.15562	0.944943	12	1.5%	1.93 [0.93, 2.93]
Morissette et al., 2020	1.884899	0.177	14	1.83817	0.266	14	1.5%	0.20 [-0.54, 0.94]
Peng et al., 2020	20.45455	3.846	10	13.6364	4.196	10	1.5%	1.62 [0.58, 2.66]
Silva-maia et al., 2018	30.30303	3.207	7	37.5758	6.414	7	1.4%	-1.34 [-2.54, -0.14]
Su et al., 2019	14.31818	6.309	6	11.7424	6.309	6	1.5%	0.38 [-0.77, 1.52]
Tian et al., 2021 (a)	6	0.821	10	2.1	0.622	10	1.3%	5.13 [3.15, 7.11]
Tian et al., 2021 (b)	9	0.92	10	2.1	0.622	10	1.0%	8.42 [5.38, 11.46]
Tian et al., 2021 (c)	6.4	0.21	10	2.1	0.622	10	0.9%	8.87 [5.68, 12.06]
Wang et al.,2020(a)	10.65743	1.4987	12	5.828286	1.1656	12	1.4%	3.47 [2.13, 4.81]
Wang et al.,2020(b)	9.6583	0.83261	12	5.828286	1.1656	12	1.4%	3.65 [2.27, 5.04]
Wang et al.,2020(c)	6.660898	1.16565	12	5.828286	1.1656	12	1.5%	0.69 [-0.14, 1.52]
Wu et al., 2018	64.75091	3.215	12	19.2875	2.985	12	0.7%	14.15 [9.70, 18.60]
**Subtotal (95% CI)**			**193**			**193**	**30.4%**	**1.81 [0.90, 2.73]**
Heterogeneity: Tau² = 4.20; Chi² = 224.57, df = 22 (P < 0.00001); I² = 90%								
Test for overall effect: Z = 3.89 (P < 0.0001)								
**3.1.3 Lower Dose**								
Hu et al., 2019 (b)	1.8235	0.021234	10	2.11628	0.039	10	0.9%	-8.93 [-12.14, -5.72]
Kapoor et al.,2022 (a)	0.216875	0.121	3	0.18935	0.032	3	1.4%	0.25 [-1.37, 1.87]
Kapoor et al., 2022 (c)	0.281609	0.057	3	0.35506	0.105	3	1.3%	-0.70 [-2.43, 1.04]
Kaur et al., 2022 (a)	0.69	0.16	4	0.71	0.32	4	1.4%	-0.07 [-1.46, 1.32]
Liu et al., 2020 (a)	4.163424	2.412	6	2.56809	0.584	6	1.4%	0.84 [-0.36, 2.04]
Liu et al., 2022 (a)	8.63947	0.80995	12	6.15562	0.944943	12	1.5%	2.73 [1.56, 3.89]
Tian et al., 2021 (a)	6	0.821	10	2.1	0.622	10	1.3%	5.13 [3.15, 7.11]
Wang et al.,2020(a)	6.660898	1.16565	12	5.828286	1.1656	12	1.5%	0.69 [-0.14, 1.52]
**Subtotal (95% CI)**			**60**			**60**	**10.7%**	**0.31 [-1.27, 1.89]**
Heterogeneity: Tau² = 4.44; Chi² = 69.02, df = 7 (P < 0.00001); I² = 90%								
Test for overall effect: Z = 0.39 (P = 0.70)								
**3.1.4 Higher Dose**								
Hu et al., 2019 (c)	2.503876	0.054	10	2.11628	0.039	10	1.0%	7.88 [5.02, 10.74]
Kapoor et al.,2022 (b)	0.58665	0.184	3	0.18935	0.032	3	1.0%	2.41 [-0.41, 5.23]
Kapoor et al., 2022 (d)	0.571131	0.119	3	0.35506	0.105	3	1.2%	1.54 [-0.64, 3.73]
Liu et al., 2020 (b)	1.21	0.51	4	0.71	0.32	4	1.4%	1.02 [-0.53, 2.58]
Liu et al., 2022 (c)	2.918288	1.323	6	2.56809	0.584	6	1.5%	0.32 [-0.83, 1.46]
Morissette et al., 2020	13.90416	5.39	12	6.15562	0.944943	12	1.5%	1.93 [0.93, 2.93]
Tian et al., 2021 (c)	6.4	0.21	10	2.1	0.622	10	0.9%	8.87 [5.68, 12.06]
Wang et al.,2020(c)	10.65743	1.4987	12	5.828286	1.1656	12	1.4%	3.47 [2.13, 4.81]
**Subtotal (95% CI)**			**60**			**60**	**9.9%**	**3.08 [1.50, 4.66]**
Heterogeneity: Tau² = 4.10; Chi² = 48.62, df = 7 (P < 0.00001); I² = 86%								
Test for overall effect: Z = 3.82 (P = 0.0001)								
**3.1.5 High Fat/Cholesterol Model**								
Lee et al., 2018	0.011497	0.002	8	0.01217	0.004	8	1.5%	-0.20 [-1.18, 0.78]
Liu et al., 2022 (a)	8.63947	0.80995	12	6.15562	0.944943	12	1.5%	2.73 [1.56, 3.89]
Liu et al., 2022 (b)	12.82423	2.5648	12	6.15562	0.944943	12	1.4%	3.33 [2.03, 4.64]
Liu et al., 2022 (c)	13.90416	5.39	12	6.15562	0.944943	12	1.5%	1.93 [0.93, 2.93]
Morissette et al., 2020	1.884899	0.177	14	1.83817	0.266	14	1.5%	0.20 [-0.54, 0.94]
Tian et al., 2021 (a)	6	0.821	10	2.1	0.622	10	1.3%	5.13 [3.15, 7.11]
Tian et al., 2021 (b)	9	0.92	10	2.1	0.622	10	1.0%	8.42 [5.38, 11.46]
Tian et al., 2021 (c)	6.4	0.21	10	2.1	0.622	10	0.9%	8.87 [5.68, 12.06]
Wang et al.,2020(a)	10.65743	1.4987	12	5.828286	1.1656	12	1.4%	3.47 [2.13, 4.81]
Wang et al.,2020(b)	9.6583	0.83261	12	5.828286	1.1656	12	1.4%	3.65 [2.27, 5.04]
Wang et al.,2020(c)	6.660898	1.16565	12	5.828286	1.1656	12	1.5%	0.69 [-0.14, 1.52]
Wu et al., 2018	64.75091	3.215	12	19.2875	2.985	12	0.7%	14.15 [9.70, 18.60]
**Subtotal (95% CI)**			**136**			**136**	**15.6%**	**3.64 [2.31, 4.97]**
Heterogeneity: Tau² = 4.67; Chi² = 138.78, df = 11 (P < 0.00001); I² = 92%								
Test for overall effect: Z = 5.35 (P < 0.00001)								
**3.1.6 Other Animal Model**								
da Silva-Maia et al., 2019 (a)	65	2.534	7	77	6.414	7	1.4%	-2.30 [-3.76, -0.85]
da Silva-Maia et al., 2019 (b)	63	1.823	7	77	6.414	7	1.4%	-2.78 [-4.38, -1.18]
Hu et al., 2019 (a)	2.503876	0.054	10	2.11628	0.039	10	1.0%	7.88 [5.02, 10.74]
Hu et al., 2019 (b)	2.2215	0.021569	10	2.11628	0.039	10	1.4%	3.20 [1.79, 4.61]
Hu et al., 2019 (c)	1.8235	0.021234	10	2.11628	0.039	10	0.9%	-8.93 [-12.14, -5.72]
Kapoor et al.,2022 (a)	0.571131	0.119	3	0.35506	0.105	3	1.2%	1.54 [-0.64, 3.73]
Kapoor et al.,2022 (b)	0.216875	0.121	3	0.18935	0.032	3	1.4%	0.25 [-1.37, 1.87]
Kapoor et al., 2022 (c)	0.58665	0.184	3	0.18935	0.032	3	1.0%	2.41 [-0.41, 5.23]
Kapoor et al., 2022 (d)	0.281609	0.057	3	0.35506	0.105	3	1.3%	-0.70 [-2.43, 1.04]
Kaur et al., 2022 (a)	0.69	0.16	4	0.71	0.32	4	1.4%	-0.07 [-1.46, 1.32]
Kaur et al., 2022 (b)	1.21	0.51	4	0.71	0.32	4	1.4%	1.02 [-0.53, 2.58]
Liu et al., 2020 (a)	4.163424	2.412	6	2.56809	0.584	6	1.4%	0.84 [-0.36, 2.04]
Liu et al., 2020 (b)	2.918288	1.323	6	2.56809	0.584	6	1.5%	0.32 [-0.83, 1.46]
Peng et al., 2019 (a)	14.8503	4.79	10	11.018	3.353	10	1.5%	0.89 [-0.04, 1.82]
Peng et al., 2019 (b)	14.37126	2.395	10	11.018	3.353	10	1.5%	1.10 [0.15, 2.06]
Peng et al., 2020	20.45455	3.846	10	13.6364	4.196	10	1.5%	1.62 [0.58, 2.66]
Silva-maia et al., 2018	30.30303	3.207	7	37.5758	6.414	7	1.4%	-1.34 [-2.54, -0.14]
Su et al., 2019	14.31818	6.309	6	11.7424	6.309	6	1.5%	0.38 [-0.77, 1.52]
**Subtotal (95% CI)**			**119**			**119**	**24.1%**	**0.33 [-0.57, 1.24]**
Heterogeneity: Tau² = 3.14; Chi² = 124.76, df = 17 (P < 0.00001); I² = 86%								
Test for overall effect: Z = 0.72 (P = 0.47)								
**Total (95% CI)**			**630**			**630**	**100.0%**	**1.61 [1.12, 2.10]**
Heterogeneity: Tau² = 3.94; Chi² = 720.47, df = 75 (P < 0.00001); I² = 90%								
Test for overall effect: Z = 6.44 (P < 0.00001)								
Test for subgroup differences: Chi² = 23.13, df = 5 (P = 0.0003), I² = 78.4%								

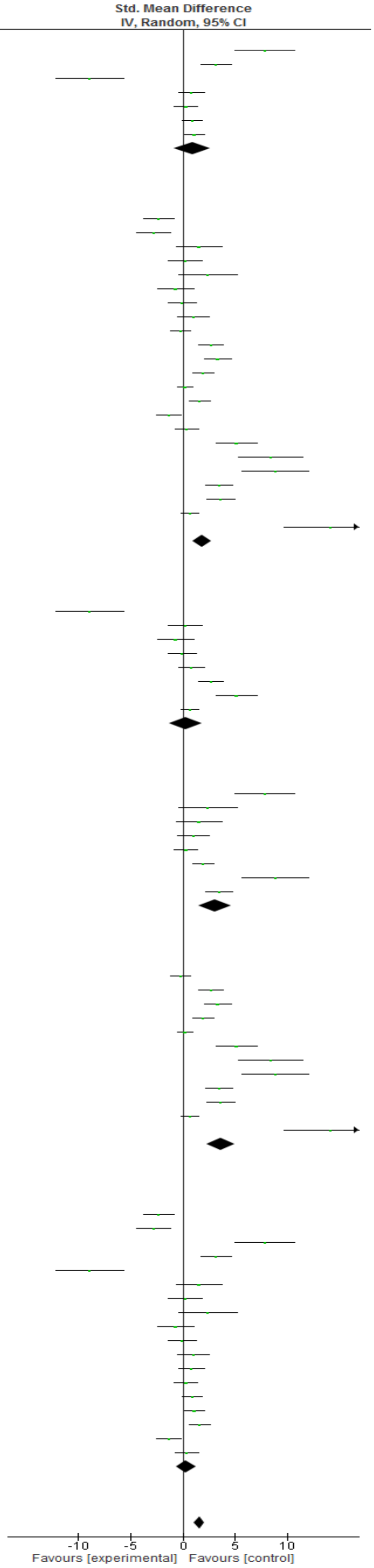

S Figure 8: Forest plot of the sub-group analysis to study effect of anthocyanin supplementation on the butanoic acid, SCFA type. Sub-grouping was on the basis of duration of the study, dose of the anthocyanin given and disease status of the subjects (animal models). Values are standardized mean differences with 95% CIs determined with the use of random-effects models. Heterogeneity was quantified by I^2, inverse variance and standardised mean difference (SMD).

Study or Subgroup	Experimental Mean	Experimental SD	Experimental Total	Control Mean	Control SD	Control Total	Weight	Std. Mean Difference IV, Random, 95% CI
**3.1.1 Study less than 4 weeks**								
Liu et al., 2020 (a)	4.163424	2.412	6	2.56809	0.584	6	2.0%	0.84 [-0.36, 2.04]
Liu et al., 2020 (b)	2.918288	1.323	6	2.56809	0.584	6	2.0%	0.32 [-0.83, 1.46]
Peng et al., 2019 (a)	14.8503	4.79	10	11.018	3.353	10	2.1%	0.89 [-0.04, 1.82]
Peng et al., 2019 (b)	14.37126	2.395	10	11.018	3.353	10	2.0%	1.10 [0.15, 2.06]
**Subtotal (95% CI)**			**32**			**32**	**8.0%**	**0.82 [0.30, 1.34]**
Heterogeneity: Tau² = 0.00; Chi² = 1.10, df = 3 (P = 0.78); I² = 0%								
Test for overall effect: Z = 3.11 (P = 0.002)								
**3.1.2 Study more than 4 weeks**								
da Silva-Maia et al., 2019 (a)	65	2.534	7	77	6.414	7	1.9%	-2.30 [-3.76, -0.85]
da Silva-Maia et al., 2019 (b)	63	1.823	7	77	6.414	7	1.8%	-2.78 [-4.38, -1.18]
Kapoor et al.,2022 (a)	0.571131	0.119	3	0.35506	0.105	3	1.5%	1.54 [-0.64, 3.73]
Kapoor et al.,2022 (b)	0.216875	0.121	3	0.18935	0.032	3	1.8%	0.25 [-1.37, 1.87]
Kapoor et al., 2022 (c)	0.58665	0.184	3	0.18935	0.032	3	1.3%	2.41 [-0.41, 5.23]
Kapoor et al., 2022 (d)	0.281609	0.057	3	0.35506	0.105	3	1.7%	-0.70 [-2.43, 1.04]
Kaur et al., 2022 (a)	0.69	0.16	4	0.71	0.32	4	1.9%	-0.07 [-1.46, 1.32]
Kaur et al., 2022 (b)	1.21	0.51	4	0.71	0.32	4	1.8%	1.02 [-0.53, 2.58]
Lee et al., 2018	0.011497	0.002	8	0.01217	0.004	8	2.0%	-0.20 [-1.18, 0.78]
Morissette et al., 2020	1.884899	0.177	14	1.83817	0.266	14	2.1%	0.20 [-0.54, 0.94]
Peng et al., 2020	20.45455	3.846	10	13.6364	4.196	10	2.0%	1.62 [0.58, 2.66]
Silva-maia et al., 2018	30.30303	3.207	7	37.5758	6.414	7	2.0%	-1.34 [-2.54, -0.14]
Su et al., 2019	14.31818	6.309	6	11.7424	6.309	6	2.0%	0.38 [-0.77, 1.52]
Tian et al., 2021 (a)	6	0.821	10	2.1	0.622	10	1.6%	5.13 [3.15, 7.11]
Tian et al., 2021 (b)	9	0.92	10	2.1	0.622	10	1.2%	8.42 [5.38, 11.46]
Tian et al., 2021 (c)	6.4	0.21	10	2.1	0.622	10	1.1%	8.87 [5.68, 12.06]
Wang et al.,2020(a)	10.65743	1.4987	12	5.828286	1.1656	12	1.9%	3.47 [2.13, 4.81]
Wang et al.,2020(b)	9.6583	0.83261	12	5.828286	1.1656	12	1.9%	3.65 [2.27, 5.04]
Wang et al.,2020(c)	6.660898	1.16565	12	5.828286	1.1656	12	2.1%	0.69 [-0.14, 1.52]
**Subtotal (95% CI)**			**145**			**145**	**33.5%**	**1.30 [0.36, 2.25]**
Heterogeneity: Tau² = 3.72; Chi² = 163.11, df = 18 (P < 0.00001); I² = 89%								
Test for overall effect: Z = 2.69 (P = 0.007)								
**3.1.3 Lower Dose**								
Kapoor et al.,2022 (a)	0.216875	0.121	3	0.18935	0.032	3	1.8%	0.25 [-1.37, 1.87]
Kapoor et al., 2022 (c)	0.281609	0.057	3	0.35506	0.105	3	1.7%	-0.70 [-2.43, 1.04]
Kaur et al., 2022 (a)	0.69	0.16	4	0.71	0.32	4	1.9%	-0.07 [-1.46, 1.32]
Tian et al., 2021 (a)	6	0.821	10	2.1	0.622	10	1.6%	5.13 [3.15, 7.11]
Wang et al.,2020(a)	6.660898	1.16565	12	5.828286	1.1656	12	2.1%	0.69 [-0.14, 1.52]
**Subtotal (95% CI)**			**32**			**32**	**9.1%**	**0.97 [-0.57, 2.50]**
Heterogeneity: Tau² = 2.47; Chi² = 23.20, df = 4 (P = 0.0001); I² = 83%								
Test for overall effect: Z = 1.23 (P = 0.22)								
**3.1.4 Higher Dose**								
Kapoor et al.,2022 (b)	0.58665	0.184	3	0.18935	0.032	3	1.3%	2.41 [-0.41, 5.23]
Kapoor et al., 2022 (d)	0.571131	0.119	3	0.35506	0.105	3	1.5%	1.54 [-0.64, 3.73]
Morissette et al., 2020	13.90416	5.39	12	6.15562	0.944943	12	2.0%	1.93 [0.93, 2.93]
Tian et al., 2021 (c)	6.4	0.21	10	2.1	0.622	10	1.1%	8.87 [5.68, 12.06]
Wang et al.,2020(c)	10.65743	1.4987	12	5.828286	1.1656	12	1.9%	3.47 [2.13, 4.81]
**Subtotal (95% CI)**			**40**			**40**	**7.8%**	**3.32 [1.53, 5.11]**
Heterogeneity: Tau² = 3.02; Chi² = 19.03, df = 4 (P = 0.0008); I² = 79%								
Test for overall effect: Z = 3.64 (P = 0.0003)								
**3.1.5 High Fat/Cholesterol Model**								
Lee et al., 2018	0.011497	0.002	8	0.01217	0.004	8	2.0%	-0.20 [-1.18, 0.78]
Morissette et al., 2020	1.884899	0.177	14	1.83817	0.266	14	2.1%	0.20 [-0.54, 0.94]
Tian et al., 2021 (a)	6	0.821	10	2.1	0.622	10	1.6%	5.13 [3.15, 7.11]
Tian et al., 2021 (b)	9	0.92	10	2.1	0.622	10	1.2%	8.42 [5.38, 11.46]
Tian et al., 2021 (c)	6.4	0.21	10	2.1	0.622	10	1.1%	8.87 [5.68, 12.06]
Wang et al.,2020(a)	10.65743	1.4987	12	5.828286	1.1656	12	1.9%	3.47 [2.13, 4.81]
Wang et al.,2020(b)	9.6583	0.83261	12	5.828286	1.1656	12	1.9%	3.65 [2.27, 5.04]
Wang et al.,2020(c)	6.660898	1.16565	12	5.828286	1.1656	12	2.1%	0.69 [-0.14, 1.52]
**Subtotal (95% CI)**			**88**			**88**	**13.9%**	**3.34 [1.65, 5.03]**
Heterogeneity: Tau² = 5.10; Chi² = 97.54, df = 7 (P < 0.00001); I² = 93%								
Test for overall effect: Z = 3.88 (P = 0.0001)								
**3.1.6 Other Animal Model**								
da Silva-Maia et al., 2019 (a)	65	2.534	7	77	6.414	7	1.9%	-2.30 [-3.76, -0.85]
da Silva-Maia et al., 2019 (b)	63	1.823	7	77	6.414	7	1.8%	-2.78 [-4.38, -1.18]
Kapoor et al.,2022 (a)	0.571131	0.119	3	0.35506	0.105	3	1.5%	1.54 [-0.64, 3.73]
Kapoor et al.,2022 (b)	0.216875	0.121	3	0.18935	0.032	3	1.8%	0.25 [-1.37, 1.87]
Kapoor et al., 2022 (c)	0.58665	0.184	3	0.18935	0.032	3	1.3%	2.41 [-0.41, 5.23]
Kapoor et al., 2022 (d)	0.281609	0.057	3	0.35506	0.105	3	1.7%	-0.70 [-2.43, 1.04]
Kaur et al., 2022 (a)	0.69	0.16	4	0.71	0.32	4	1.9%	-0.07 [-1.46, 1.32]
Kaur et al., 2022 (b)	1.21	0.51	4	0.71	0.32	4	1.8%	1.02 [-0.53, 2.58]
Liu et al., 2020 (a)	4.163424	2.412	6	2.56809	0.584	6	2.0%	0.84 [-0.36, 2.04]
Liu et al., 2020 (b)	2.918288	1.323	6	2.56809	0.584	6	2.0%	0.32 [-0.83, 1.46]
Peng et al., 2019 (a)	14.8503	4.79	10	11.018	3.353	10	2.1%	0.89 [-0.04, 1.82]
Peng et al., 2019 (b)	14.37126	2.395	10	11.018	3.353	10	2.0%	1.10 [0.15, 2.06]
Peng et al., 2020	20.45455	3.846	10	13.6364	4.196	10	2.0%	1.62 [0.58, 2.66]
Silva-maia et al., 2018	30.30303	3.207	7	37.5758	6.414	7	2.0%	-1.34 [-2.54, -0.14]
Su et al., 2019	14.31818	6.309	6	11.7424	6.309	6	2.0%	0.38 [-0.77, 1.52]
**Subtotal (95% CI)**			**89**			**89**	**27.6%**	**0.17 [-0.49, 0.83]**
Heterogeneity: Tau² = 1.17; Chi² = 50.67, df = 14 (P < 0.00001); I² = 72%								
Test for overall effect: Z = 0.51 (P = 0.61)								
**Total (95% CI)**			**426**			**426**	**100.0%**	**1.30 [0.83, 1.78]**
Heterogeneity: Tau² = 2.67; Chi² = 399.01, df = 55 (P < 0.00001); I² = 86%								
Test for overall effect: Z = 5.34 (P < 0.00001)								
Test for subgroup differences: Chi² = 20.54, df = 5 (P = 0.0010), I² = 75.7%								

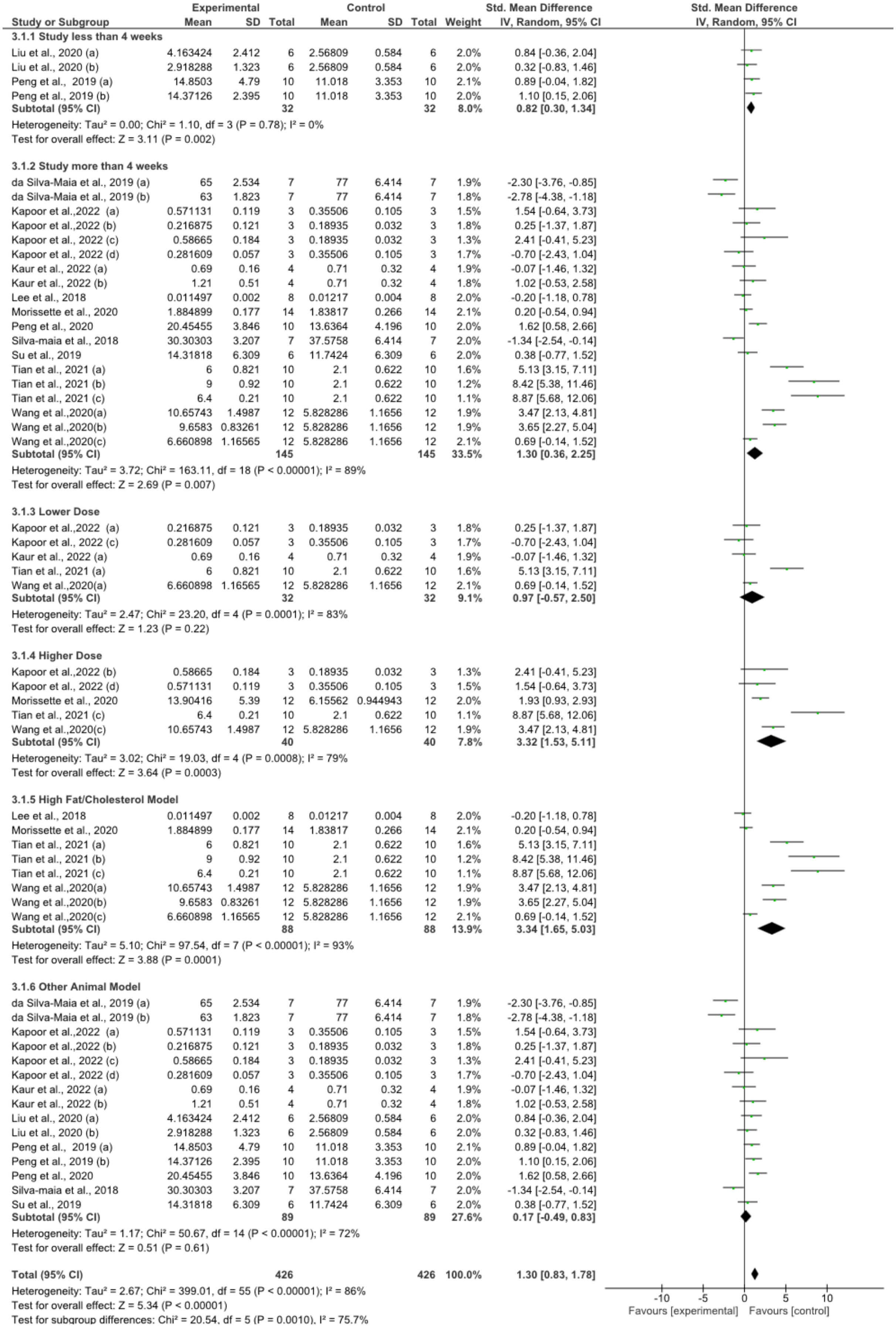

S Figure 9: Forest plot of the sub-group analysis after elimination of high-influencer studies, for understanding the effect of anthocyanin supplementation on the butanoic acid, SCFA type. Sub-grouping was on the basis of duration of the study, dose of the anthocyanin given and disease status of the subjects (animal models). Values are standardized mean differences with 95% CIs determined with the use of random-effects models. Heterogeneity was quantified by I^2, inverse variance and standardised mean difference (SMD).

Study or Subgroup	Experimental Mean	SD	Total	Control Mean	SD	Total	Weight	Std. Mean Difference IV, Random, 95% CI
**2.1.1 Study less than 4 week**								
Hu et al., 2019 (a)	6.09876543	0.395	10	4.54321	0.321	10	1.4%	4.14 [2.46, 5.82]
Hu et al., 2019 (b)	6.1	0.4253	10	4.54321	0.321	10	1.4%	3.96 [2.33, 5.58]
Hu et al., 2019 (c)	6.2	0.5214	10	4.54321	0.321	10	1.4%	3.67 [2.12, 5.21]
Liu et al., 2020 (a)	3.92395437	0.669	6	4.92776	0.578	6	1.4%	-1.48 [-2.83, -0.14]
Liu et al., 2020 (b)	4.22813688	0.943	6	4.92776	0.578	6	1.4%	-0.83 [-2.03, 0.38]
Peng et al., 2019 (a)	14.37125749	1.916	10	10.5389	3.832	10	1.5%	1.21 [0.24, 2.18]
Peng et al., 2019 (b)	11.9760479	1.437	10	10.5389	3.832	10	1.5%	0.48 [-0.42, 1.37]
**Subtotal (95% CI)**			**62**			**62**	**10.0%**	**1.52 [0.01, 3.03]**
Heterogeneity: Tau² = 3.69; Chi² = 61.09, df = 6 (P < 0.00001); I² = 90%								
Test for overall effect: Z = 1.97 (P = 0.05)								
**2.1.2 Study equal to or more than 4 week**								
da Silva-Maia et al., 2019 (a)	22.31313131	0.256	7	26	1.138	7	1.3%	-4.18 [-6.29, -2.08]
da Silva-Maia et al., 2019 (b)	25.123	0.623	7	26	1.138	7	1.5%	-0.89 [-2.01, 0.22]
Kapoor et al.,2022 (a)	0.17168	0.135	3	0.00979	0.007	3	1.3%	1.35 [-0.71, 3.42]
Kapoor et al.,2022 (b)	0.303184	0.134	3	0.03512	0.002	3	1.1%	2.26 [-0.45, 4.97]
Kapoor et al., 2022 (c)	0.029368	0.01	3	0.0097	0.007	3	1.2%	1.82 [-0.56, 4.20]
Kapoor et al., 2022 (d)	0.279591	0.076	3	0.03512	0.002	3	0.9%	3.64 [-0.22, 7.50]
Kaur et al., 2022 (a)	2.4	0.66	4	2.2	0.88	4	1.4%	0.22 [-1.17, 1.62]
Kaur et al., 2022 (b)	0.28101528	0.037	8	0.23958	0.051	8	1.5%	0.88 [-0.16, 1.92]
Lee et al., 2018	5.6696	0.674959	12	2.15987	0.27	12	1.3%	6.59 [4.40, 8.78]
Liu et al., 2022 (a)	7.5595442	0.809951	12	2.15987	0.27	12	1.1%	8.64 [5.85, 11.43]
Liu et al., 2022 (b)	11.744291	1.079934	12	2.15987	0.27	12	0.9%	11.76 [8.03, 15.48]
Liu et al., 2022 (c)	0.592058	0.177	14	0.73889	0.266	14	1.5%	-0.63 [-1.39, 0.13]
Morissette et al., 2020	3.055556	0.833	10	3.19444	0.694	10	1.5%	-0.17 [-1.05, 0.71]
Peng et al., 2020	8.695652	3.704	7	10.4348	4.762	7	1.5%	-0.38 [-1.44, 0.68]
Silva-maia et al., 2018	18.10606	2.598	6	13.1061	3.711	6	1.4%	1.44 [0.11, 2.77]
Su et al., 2019	8.26	1.741	10	1.8	0.246	10	1.3%	4.98 [3.04, 6.91]
Tian et al., 2021 (a)	12	1.56	10	1.8	0.246	10	1.1%	8.75 [5.60, 11.90]
Tian et al., 2021 (b)	8.5	0.921	10	1.8	0.246	10	1.0%	9.52 [6.11, 12.93]
Tian et al., 2021 (c)	3.914	0.6749	12	2.15983	0.54	12	1.5%	2.77 [1.60, 3.94]
Wang et al.,2020(a)	5.9396	0.5399	12	2.15983	0.54	12	1.2%	6.76 [4.52, 9.00]
Wang et al.,2020(b)	7.42440605	1.08	12	2.15983	0.54	12	1.3%	5.95 [3.94, 7.96]
Wang et al.,2020(c)	15.02047	1.092	12	12.5626	2.458	12	1.5%	1.25 [0.36, 2.14]
Wu et al., 2018	15.02047	1.092	12	12.5626	2.458	12	1.5%	1.25 [0.36, 2.14]
**Subtotal (95% CI)**			**201**			**201**	**29.7%**	**2.82 [1.74, 3.91]**
Heterogeneity: Tau² = 5.94; Chi² = 276.54, df = 22 (P < 0.00001); I² = 92%								
Test for overall effect: Z = 5.11 (P < 0.00001)								
**2.1.3 Lower Dose**								
Hu et al., 2019 (b)	6.098765	0.395	10	4.54321	0.321	10	1.4%	4.14 [2.46, 5.82]
Kapoor et al.,2022 (a)	0.17168	0.135	3	0.00979	0.007	3	1.3%	1.35 [-0.71, 3.42]
Kapoor et al., 2022 (c)	0.029368	0.01	3	0.0097	0.007	3	1.2%	1.82 [-0.56, 4.20]
Kaur et al., 2022 (a)	1.7	0.38	4	2.2	0.88	4	1.4%	-0.64 [-2.10, 0.81]
Liu et al., 2020 (a)	3.923954	0.669	6	4.92776	0.578	6	1.4%	-1.48 [-2.83, -0.14]
Liu et al., 2022 (a)	5.6696	0.674959	12	2.15987	0.27	12	1.3%	6.59 [4.40, 8.78]
Tian et al., 2021 (a)	8.26	1.741	10	1.8	0.246	10	1.3%	4.98 [3.04, 6.91]
Wang et al.,2020(a)	3.914	0.6749	12	2.15983	0.54	12	1.5%	2.77 [1.60, 3.94]
**Subtotal (95% CI)**			**60**			**60**	**10.7%**	**2.39 [0.48, 4.30]**
Heterogeneity: Tau² = 6.75; Chi² = 72.68, df = 7 (P < 0.00001); I² = 90%								
Test for overall effect: Z = 2.45 (P = 0.01)								
**2.1.4 Higher Dose**								
Hu et al., 2019 (c)	6.2	0.5214	10	4.54321	0.321	10	1.4%	3.67 [2.12, 5.21]
Kapoor et al.,2022 (b)	0.303184	0.134	3	0.03512	0.002	3	1.1%	2.26 [-0.45, 4.97]
Kapoor et al., 2022 (d)	0.279591	0.076	3	0.03512	0.002	3	0.9%	3.64 [-0.22, 7.50]
Kaur et al., 2022 (b)	2.2	0.4412	4	2.2	0.88	4	1.4%	0.00 [-1.39, 1.39]
Liu et al., 2020 (b)	4.228137	0.943	6	4.92776	0.578	6	1.4%	-0.83 [-2.03, 0.38]
Liu et al., 2022 (c)	11.74429	1.079934	12	2.15987	0.27	12	0.9%	11.76 [8.03, 15.48]
Tian et al., 2021 (c)	8.5	0.921	10	1.8	0.246	10	1.0%	9.52 [6.11, 12.93]
Wang et al.,2020(c)	7.424406	1.08	12	2.15983	0.54	12	1.3%	5.95 [3.94, 7.96]
**Subtotal (95% CI)**			**60**			**60**	**9.5%**	**4.24 [1.60, 6.87]**
Heterogeneity: Tau² = 12.73; Chi² = 94.37, df = 7 (P < 0.00001); I² = 93%								
Test for overall effect: Z = 3.15 (P = 0.002)								
**2.1.5 High Fat/Cholesterol Model**								
Lee et al., 2018	0.281015	0.037	8	0.23958	0.051	8	1.5%	0.88 [-0.16, 1.92]
Liu et al., 2022 (a)	5.6696	0.674959	12	2.15987	0.27	12	1.3%	6.59 [4.40, 8.78]
Liu et al., 2022 (b)	7.559544	0.809951	12	2.15987	0.27	12	1.1%	8.64 [5.85, 11.43]
Liu et al., 2022 (c)	11.74429	1.079934	12	2.15987	0.27	12	0.9%	11.76 [8.03, 15.48]
Morissette et al., 2020	0.592058	0.177	14	0.73889	0.266	14	1.5%	-0.63 [-1.39, 0.13]
Tian et al., 2021 (a)	8.26	1.741	10	1.8	0.246	10	1.3%	4.98 [3.04, 6.91]
Tian et al., 2021 (b)	12	1.56	10	1.8	0.246	10	1.1%	8.75 [5.60, 11.90]
Tian et al., 2021 (c)	8.5	0.921	10	1.8	0.246	10	1.0%	9.52 [6.11, 12.93]
Wang et al.,2020(a)	7.424406	1.08	12	2.15983	0.54	12	1.3%	5.95 [3.94, 7.96]
Wang et al.,2020(b)	5.9396	0.5399	12	2.15983	0.54	12	1.2%	6.76 [4.52, 9.00]
Wang et al.,2020(c)	3.914	0.6749	12	2.15983	0.54	12	1.5%	2.77 [1.60, 3.94]
Wu et al., 2018	15.02047	1.092	12	12.5626	2.458	12	1.5%	1.25 [0.36, 2.14]
**Subtotal (95% CI)**			**136**			**136**	**15.1%**	**5.27 [3.38, 7.16]**
Heterogeneity: Tau² = 9.87; Chi² = 197.10, df = 11 (P < 0.00001); I² = 94%								
Test for overall effect: Z = 5.46 (P < 0.00001)								
**2.1.6 Other Animal Models**								
da Silva-Maia et al., 2019 (a)	22.31313	0.256	7	26	1.138	7	1.3%	-4.18 [-6.29, -2.08]
da Silva-Maia et al., 2019 (b)	25.123	0.623	7	26	1.138	7	1.5%	-0.89 [-2.01, 0.22]
Hu et al., 2019 (a)	6.098765	0.395	10	4.54321	0.321	10	1.4%	4.14 [2.46, 5.82]
Hu et al., 2019 (b)	6.1	0.4253	10	4.54321	0.321	10	1.4%	3.96 [2.33, 5.58]
Hu et al., 2019 (c)	6.2	0.5214	10	4.54321	0.321	10	1.4%	3.67 [2.12, 5.21]
Kapoor et al.,2022 (a)	0.17168	0.135	3	0.00979	0.007	3	1.3%	1.35 [-0.71, 3.42]
Kapoor et al.,2022 (b)	0.094367	0.071	3	0.03512	0.002	3	1.3%	0.94 [-0.90, 2.79]
Kapoor et al., 2022 (c)	0.303184	0.134	3	0.03512	0.002	3	1.1%	2.26 [-0.45, 4.97]
Kapoor et al., 2022 (d)	0.029368	0.01	3	0.0097	0.007	3	1.2%	1.82 [-0.56, 4.20]
Kaur et al., 2022 (a)	1.7	0.38	4	2.2	0.88	4	1.4%	-0.64 [-2.10, 0.81]
Kaur et al., 2022 (b)	2.2	0.4412	4	2.2	0.88	4	1.4%	0.00 [-1.39, 1.39]
Liu et al., 2020 (a)	3.923954	0.669	6	4.92776	0.578	6	1.4%	-1.48 [-2.83, -0.14]
Liu et al., 2020 (b)	4.228137	0.943	6	4.92776	0.578	6	1.4%	-0.83 [-2.03, 0.38]
Peng et al., 2019 (a)	14.37126	1.916	10	10.5389	3.832	10	1.5%	1.21 [0.24, 2.18]
Peng et al., 2019 (b)	11.97605	1.437	10	10.5389	3.832	10	1.5%	0.48 [-0.42, 1.37]
Peng et al., 2020	3.055556	0.833	10	3.19444	0.694	10	1.5%	-0.17 [-1.05, 0.71]
Silva-maia et al., 2018	8.695652	3.704	7	10.4348	4.762	7	1.5%	-0.38 [-1.44, 0.68]
Su et al., 2019	18.10606	2.598	6	13.1061	3.711	6	1.4%	1.44 [0.11, 2.77]
**Subtotal (95% CI)**			**119**			**119**	**24.9%**	**0.65 [-0.15, 1.45]**
Heterogeneity: Tau² = 2.40; Chi² = 104.95, df = 17 (P < 0.00001); I² = 84%								
Test for overall effect: Z = 1.59 (P = 0.11)								
**Total (95% CI)**			**638**			**638**	**100.0%**	**2.55 [1.98, 3.12]**
Heterogeneity: Tau² = 5.46; Chi² = 861.26, df = 75 (P < 0.00001); I² = 91%								
Test for overall effect: Z = 8.79 (P < 0.00001)								
Test for subgroup differences: Chi² = 27.75, df = 5 (P < 0.0001), I² = 82.0%								

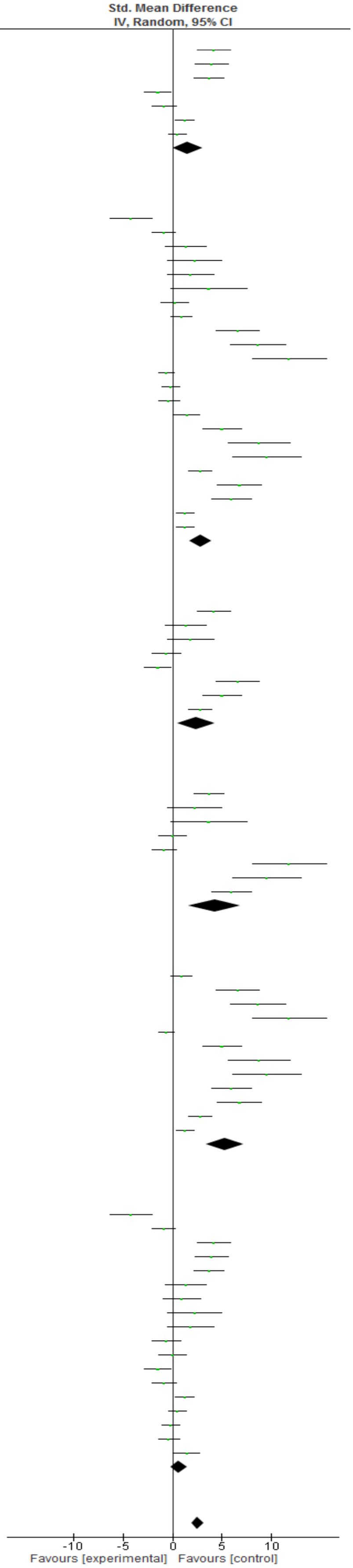

S Figure 10: Forest plot of the sub-group analysis to study effect of anthocyanin supplementation on the propionic acid, SCFA type. Sub-grouping was on the basis of duration of the study, dose of the anthocyanin given and disease status of the subjects (animal models). Values are standardized mean differences with 95% CIs determined with the use of random-effects models. Heterogeneity was quantified by I^2, inverse variance and standardised mean difference (SMD).

Study or Subgroup	Experimental Mean	Experimental SD	Experimental Total	Control Mean	Control SD	Control Total	Weight	Std. Mean Difference IV, Random, 95% CI
**2.1.1 Study less than 4 week**								
Liu et al., 2020 (a)	3.92395437	0.669	6	4.92776	0.578	6	1.9%	-1.48 [-2.83, -0.14]
Liu et al., 2020 (b)	4.22813688	0.943	6	4.92776	0.578	6	1.9%	-0.83 [-2.03, 0.38]
Peng et al., 2019 (a)	14.37125749	1.916	10	10.5389	3.832	10	2.0%	1.21 [0.24, 2.18]
Peng et al., 2019 (b)	11.9760479	1.437	10	10.5389	3.832	10	2.0%	0.48 [-0.42, 1.37]
**Subtotal (95% CI)**			**32**			**32**	**7.9%**	**-0.08 [-1.22, 1.06]**
Heterogeneity: Tau² = 1.04; Chi² = 13.30, df = 3 (P = 0.004); I² = 77%								
Test for overall effect: Z = 0.14 (P = 0.89)								
**2.1.2 Study equal to or more than 4 week**								
da Silva-Maia et al., 2019 (a)	22.31313131	0.256	7	26	1.138	7	1.7%	-4.18 [-6.29, -2.08]
da Silva-Maia et al., 2019 (b)	25.123	0.623	7	26	1.138	7	2.0%	-0.89 [-2.01, 0.22]
Kapoor et al.,2022 (a)	0.17168	0.135	3	0.00979	0.007	3	1.7%	1.35 [-0.71, 3.42]
Kapoor et al.,2022 (b)	0.303184	0.134	3	0.03512	0.002	3	1.5%	2.26 [-0.45, 4.97]
Kapoor et al., 2022 (c)	0.029368	0.01	3	0.0097	0.007	3	1.6%	1.82 [-0.56, 4.20]
Kapoor et al., 2022 (d)	0.279591	0.076	3	0.03512	0.002	3	1.1%	3.64 [-0.22, 7.50]
Kaur et al., 2022 (a)	2.4	0.66	4	2.2	0.88	4	1.9%	0.22 [-1.17, 1.62]
Kaur et al., 2022 (b)	0.28101528	0.037	8	0.23958	0.051	8	2.0%	0.88 [-0.16, 1.92]
Lee et al., 2018	5.6696	0.674959	12	2.15987	0.27	12	1.6%	6.59 [4.40, 8.78]
Morissette et al., 2020	3.055556	0.833	10	3.19444	0.694	10	2.0%	-0.17 [-1.05, 0.71]
Peng et al., 2020	8.695652	3.704	7	10.4348	4.762	7	2.0%	-0.38 [-1.44, 0.68]
Silva-maia et al., 2018	18.10606	2.598	6	13.1061	3.711	6	1.9%	1.44 [0.11, 2.77]
Su et al., 2019	8.26	1.741	10	1.8	0.246	10	1.7%	4.98 [3.04, 6.91]
Tian et al., 2021 (a)	12	1.56	10	1.8	0.246	10	1.3%	8.75 [5.60, 11.90]
Tian et al., 2021 (b)	8.5	0.921	10	1.8	0.246	10	1.2%	9.52 [6.11, 12.93]
Tian et al., 2021 (c)	3.914	0.6749	12	2.15983	0.54	12	2.0%	2.77 [1.60, 3.94]
Wang et al.,2020(a)	5.9396	0.5399	12	2.15983	0.54	12	1.6%	6.76 [4.52, 9.00]
Wang et al.,2020(b)	7.42440605	1.08	12	2.15983	0.54	12	1.7%	5.95 [3.94, 7.96]
Wang et al.,2020(c)	15.02047	1.092	12	12.5626	2.458	12	2.0%	1.25 [0.36, 2.14]
Wu et al., 2018	15.02047	1.092	12	12.5626	2.458	12	2.0%	1.25 [0.36, 2.14]
**Subtotal (95% CI)**			**163**			**163**	**34.5%**	**2.40 [1.34, 3.47]**
Heterogeneity: Tau² = 4.93; Chi² = 196.01, df = 19 (P < 0.00001); I² = 90%								
Test for overall effect: Z = 4.42 (P < 0.00001)								
**2.1.3 Lower Dose**								
Kapoor et al.,2022 (a)	0.17168	0.135	3	0.00979	0.007	3	1.7%	1.35 [-0.71, 3.42]
Kapoor et al., 2022 (c)	0.029368	0.01	3	0.0097	0.007	3	1.6%	1.82 [-0.56, 4.20]
Kaur et al., 2022 (a)	1.7	0.38	4	2.2	0.88	4	1.9%	-0.64 [-2.10, 0.81]
Tian et al., 2021 (a)	8.26	1.741	10	1.8	0.246	10	1.7%	4.98 [3.04, 6.91]
Wang et al.,2020(a)	3.914	0.6749	12	2.15983	0.54	12	2.0%	2.77 [1.60, 3.94]
**Subtotal (95% CI)**			**32**			**32**	**8.8%**	**2.03 [0.16, 3.91]**
Heterogeneity: Tau² = 3.70; Chi² = 23.84, df = 4 (P < 0.0001); I² = 83%								
Test for overall effect: Z = 2.13 (P = 0.03)								
**2.1.4 Higher Dose**								
Kapoor et al.,2022 (b)	0.303184	0.134	3	0.03512	0.002	3	1.5%	2.26 [-0.45, 4.97]
Kapoor et al., 2022 (d)	0.279591	0.076	3	0.03512	0.002	3	1.1%	3.64 [-0.22, 7.50]
Kaur et al., 2022 (b)	2.2	0.4412	4	2.2	0.88	4	1.9%	0.00 [-1.39, 1.39]
Tian et al., 2021 (c)	8.5	0.921	10	1.8	0.246	10	1.2%	9.52 [6.11, 12.93]
Wang et al.,2020(c)	7.424406	1.08	12	2.15983	0.54	12	1.7%	5.95 [3.94, 7.96]
**Subtotal (95% CI)**			**32**			**32**	**7.4%**	**4.15 [0.73, 7.57]**
Heterogeneity: Tau² = 13.24; Chi² = 40.38, df = 4 (P < 0.00001); I² = 90%								
Test for overall effect: Z = 2.38 (P = 0.02)								
**2.1.5 High Fat/Cholesterol Model**								
Lee et al., 2018	0.281015	0.037	8	0.23958	0.051	8	2.0%	0.88 [-0.16, 1.92]
Morissette et al., 2020	0.592058	0.177	14	0.73889	0.266	14	2.0%	-0.63 [-1.39, 0.13]
Tian et al., 2021 (a)	8.26	1.741	10	1.8	0.246	10	1.7%	4.98 [3.04, 6.91]
Tian et al., 2021 (b)	12	1.56	10	1.8	0.246	10	1.3%	8.75 [5.60, 11.90]
Tian et al., 2021 (c)	8.5	0.921	10	1.8	0.246	10	1.2%	9.52 [6.11, 12.93]
Wang et al.,2020(a)	7.424406	1.08	12	2.15983	0.54	12	1.7%	5.95 [3.94, 7.96]
Wang et al.,2020(b)	5.9396	0.5399	12	2.15983	0.54	12	1.6%	6.76 [4.52, 9.00]
Wang et al.,2020(c)	3.914	0.6749	12	2.15983	0.54	12	2.0%	2.77 [1.60, 3.94]
**Subtotal (95% CI)**			**88**			**88**	**13.6%**	**4.60 [2.30, 6.90]**
Heterogeneity: Tau² = 9.87; Chi² = 128.81, df = 7 (P < 0.00001); I² = 95%								
Test for overall effect: Z = 3.92 (P < 0.0001)								
**2.1.6 Other Animal Models**								
da Silva-Maia et al., 2019 (a)	22.31313	0.256	7	26	1.138	7	1.7%	-4.18 [-6.29, -2.08]
da Silva-Maia et al., 2019 (b)	25.123	0.623	7	26	1.138	7	2.0%	-0.89 [-2.01, 0.22]
Hu et al., 2019 (a)	6.098765	0.395	10	4.54321	0.321	10	1.8%	4.14 [2.46, 5.82]
Kapoor et al.,2022 (b)	0.094367	0.071	3	0.03512	0.002	3	1.8%	0.94 [-0.90, 2.79]
Kapoor et al., 2022 (c)	0.303184	0.134	3	0.03512	0.002	3	1.5%	2.26 [-0.45, 4.97]
Kapoor et al., 2022 (d)	0.029368	0.01	3	0.0097	0.007	3	1.6%	1.82 [-0.56, 4.20]
Kaur et al., 2022 (a)	1.7	0.38	4	2.2	0.88	4	1.9%	-0.64 [-2.10, 0.81]
Kaur et al., 2022 (b)	2.2	0.4412	4	2.2	0.88	4	1.9%	0.00 [-1.39, 1.39]
Liu et al., 2020 (a)	3.923954	0.669	6	4.92776	0.578	6	1.9%	-1.48 [-2.83, -0.14]
Liu et al., 2020 (b)	4.228137	0.943	6	4.92776	0.578	6	1.9%	-0.83 [-2.03, 0.38]
Peng et al., 2019 (a)	14.37126	1.916	10	10.5389	3.832	10	2.0%	1.21 [0.24, 2.18]
Peng et al., 2019 (b)	11.97605	1.437	10	10.5389	3.832	10	2.0%	0.48 [-0.42, 1.37]
Peng et al., 2020	3.055556	0.833	10	3.19444	0.694	10	2.0%	-0.17 [-1.05, 0.71]
Silva-maia et al., 2018	8.695652	3.704	7	10.4348	4.762	7	2.0%	-0.38 [-1.44, 0.68]
Su et al., 2019	18.10606	2.598	6	13.1061	3.711	6	1.9%	1.44 [0.11, 2.77]
**Subtotal (95% CI)**			**96**			**96**	**27.8%**	**0.19 [-0.56, 0.93]**
Heterogeneity: Tau² = 1.59; Chi² = 65.81, df = 14 (P < 0.00001); I² = 79%								
Test for overall effect: Z = 0.49 (P = 0.62)								
**Total (95% CI)**			**443**			**443**	**100.0%**	**1.93 [1.34, 2.52]**
Heterogeneity: Tau² = 4.26; Chi² = 536.96, df = 56 (P < 0.00001); I² = 90%								
Test for overall effect: Z = 6.42 (P < 0.00001)								
Test for subgroup differences: Chi² = 28.52, df = 5 (P < 0.0001), I² = 82.5%								

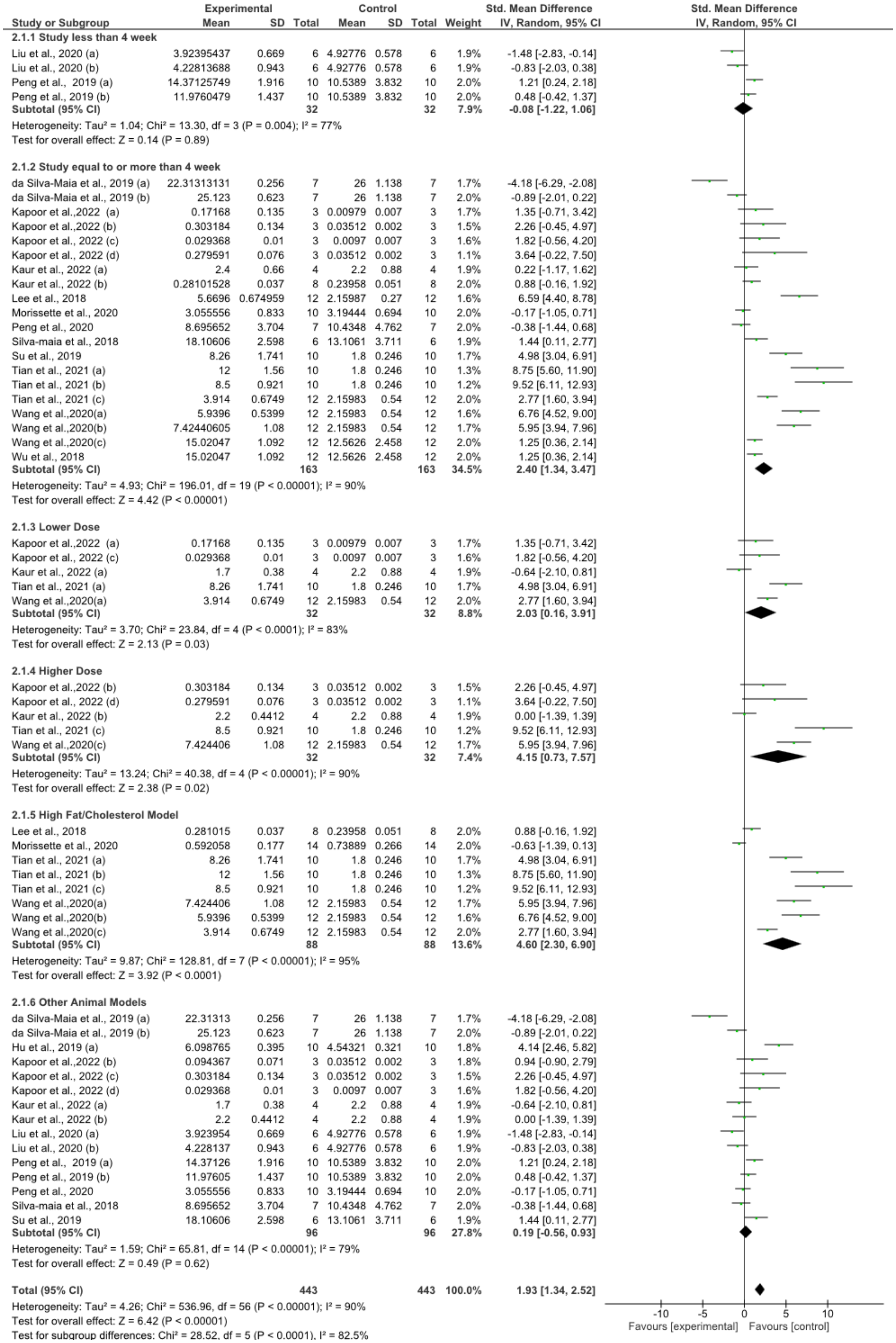

S Figure 11: Forest plot of the sub-group analysis after elimination of high-influencer studies, for understanding the effect of anthocyanin supplementation on the propionic acid, SCFA type. Sub-grouping was on the basis of duration of the study, dose of the anthocyanin given and disease status of the subjects (animal models). Values are standardized mean differences with 95% CIs determined with the use of random-effects models. Heterogeneity was quantified by I^2, inverse variance and standardised mean difference (SMD).

Question: Should Anthocyanin Rich vs Normal Diet be used for SCFA (Acetic Acid)?
Bibliography: . [Intervention] for. Cochrane Database of Systematic Reviews [Year], Issue [Issue].

Quality assessment							Summary of Findings				
Participants (studies) Follow up	Risk of bias	Inconsistency	Indirectness	Imprecision	Publication bias	Overall quality of evidence	Study event rates (%)		Relative effect (95% CI)	Anticipated absolute effects	
							With Normal Diet	With Anthocyanin Rich		Risk with Normal Diet	Risk difference with Anthocyanin Rich (95% CI)
**Acetic Acid** (Better indicated by lower values)											
1060 (23 studies)	no serious risk of bias	serious[1]	no serious indirectness	no serious imprecision	undetected	⊕⊕⊕⊕ **HIGH**[1] due to inconsistency, large effect	530	530	-		The mean acetic acid in the intervention groups was **1.52 standard deviations higher** (0.86 to 2.17 higher)

[1] High heterogeneity

Question: Should Anthocyanin Rich vs Normal Diet be used for SCFA (Propoinic Acid)?
Bibliography: . [Intervention] for. Cochrane Database of Systematic Reviews [Year], Issue [Issue].

Quality assessment							Summary of Findings				
Participants (studies) Follow up	Risk of bias	Inconsistency	Indirectness	Imprecision	Publication bias	Overall quality of evidence	Study event rates (%)		Relative effect (95% CI)	Anticipated absolute effects	
							With Normal Diet	With Anthocyanin Rich		Risk with Normal Diet	Risk difference with Anthocyanin Rich (95% CI)
**Propoinic Acid** (Better indicated by lower values)											
1048 (25 studies)	no serious risk of bias	serious[1]	no serious indirectness	no serious imprecision	undetected	⊕⊕⊕⊕ **HIGH**[1] due to inconsistency, large effect	524	524	-		The mean propoinic acid in the intervention groups was **1.44 standard deviations higher** (0.56 to 2.23 higher)

[1] High heterogeneity

Question: Should Anthocyanin Rich vs Normal Diet be used for SCFA (Butanoic Acid)?
Bibliography: . [Intervention] for. Cochrane Database of Systematic Reviews [Year], Issue [Issue].

Quality assessment							Summary of Findings				
Participants (studies) Follow up	Risk of bias	Inconsistency	Indirectness	Imprecision	Publication bias	Overall quality of evidence	Study event rates (%)		Relative effect (95% CI)	Anticipated absolute effects	
							With Normal Diet	With Anthocyanin Rich		Risk with Normal Diet	Risk difference with Anthocyanin Rich (95% CI)
**Butanoic Acid** (Better indicated by lower values)											
1048 (23 studies)	no serious risk of bias	serious[1]	no serious indirectness	no serious imprecision	undetected	⊕⊕⊕⊕ **HIGH**[1] due to inconsistency, large effect	524	524	-		The mean butanoic acid in the intervention groups was **1.15 standard deviations higher** (0.41 to 1.9 higher)

[1] High heterogeneity

S Figure 12: SCFA data quality assessment using GRADE tool.

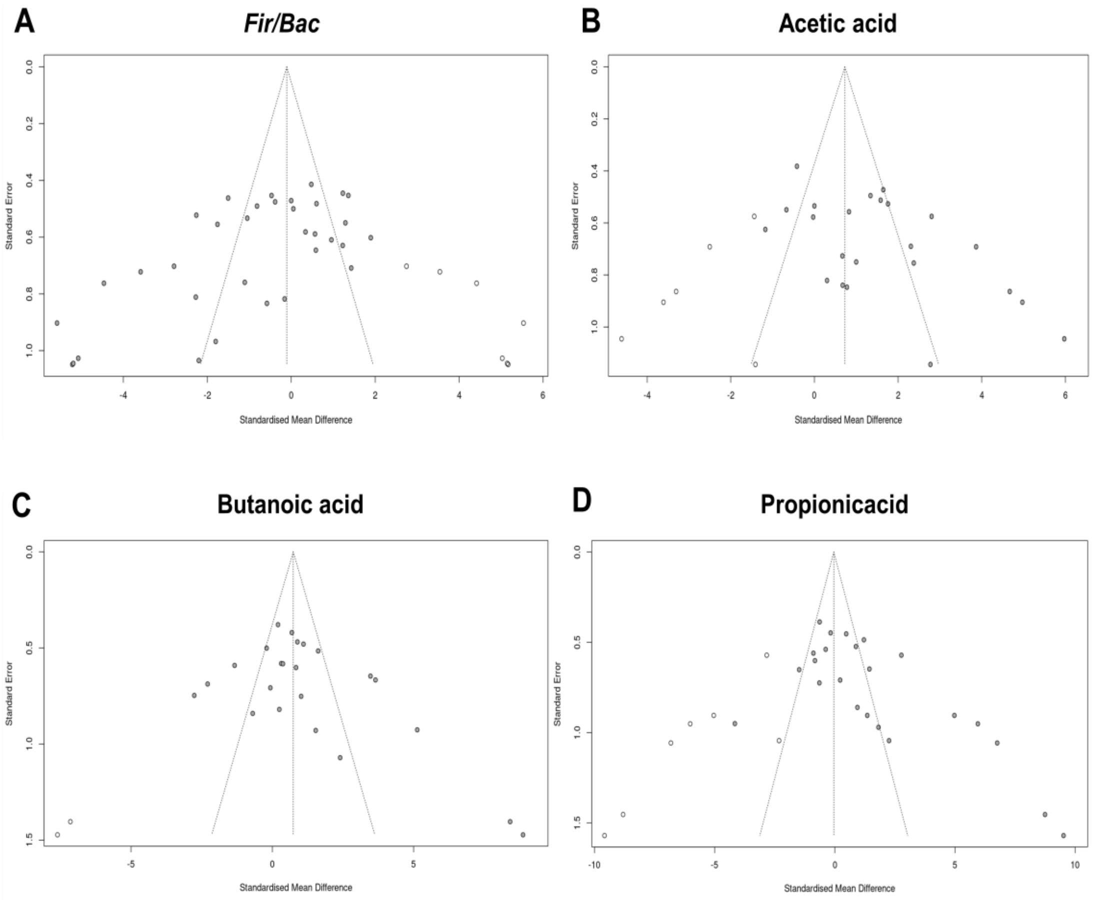

S Figure 13: **Funnel plots for publication bias assessment(A)** For *Fir/Bac*using data from 41 studies. **(B)**For Acetic acid using data from 29 studies. **(C)** For Butanoic acid using data from 25 studies. **(D)**For Propionic acid using data from 30 studies.

The plot was created by putting the standard error on the y-axis and SMD on the x-axis

A

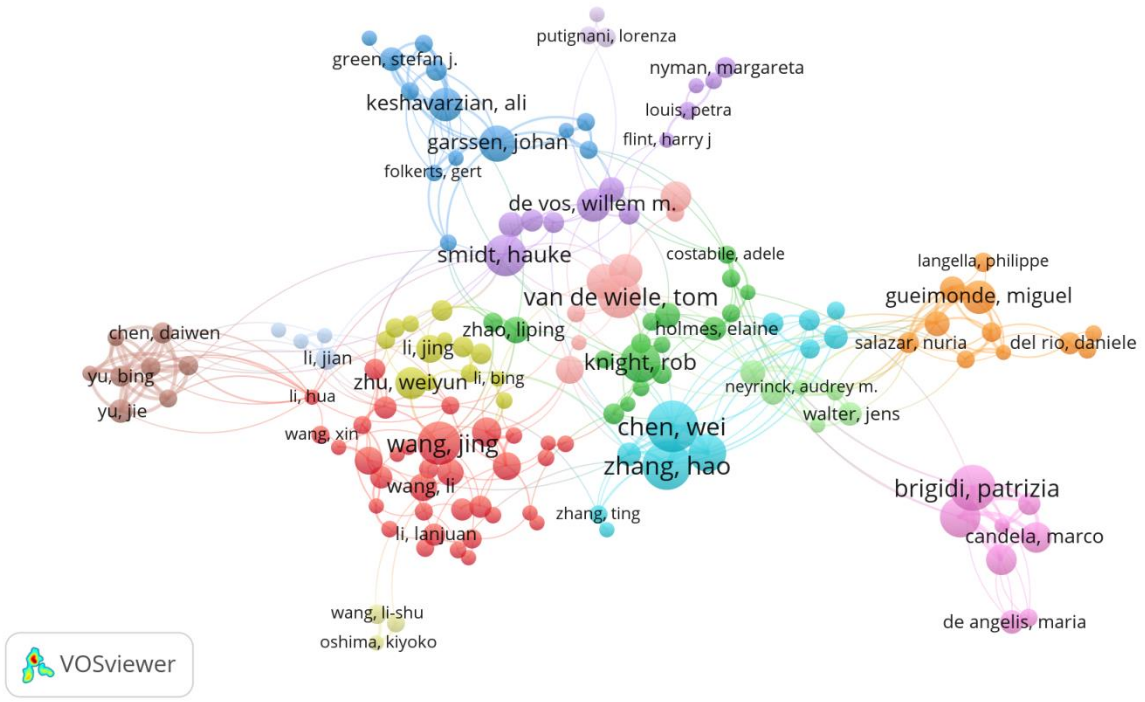

B

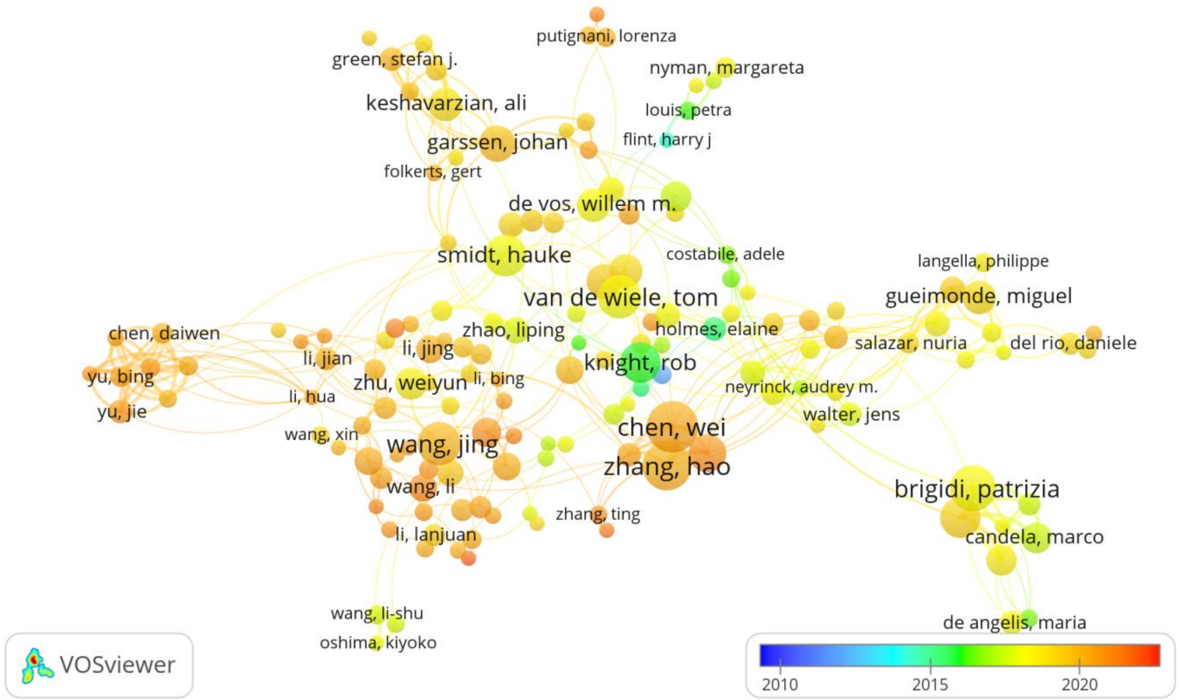

S Figure 14: Bibliographic coupling of leading researchers in anthocyanin, gut microbiota and SCFA research (A) Network visualization **(B)** Overlay visualization (year wise)

A

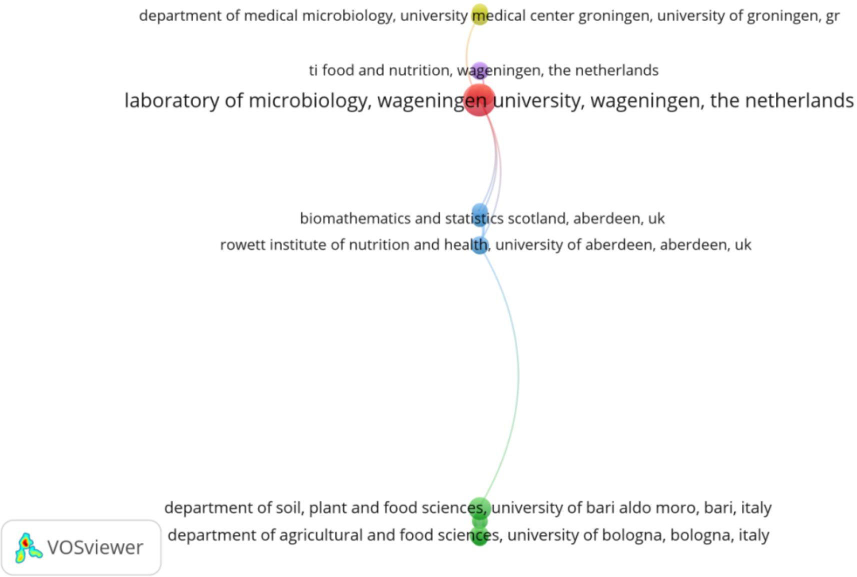

B

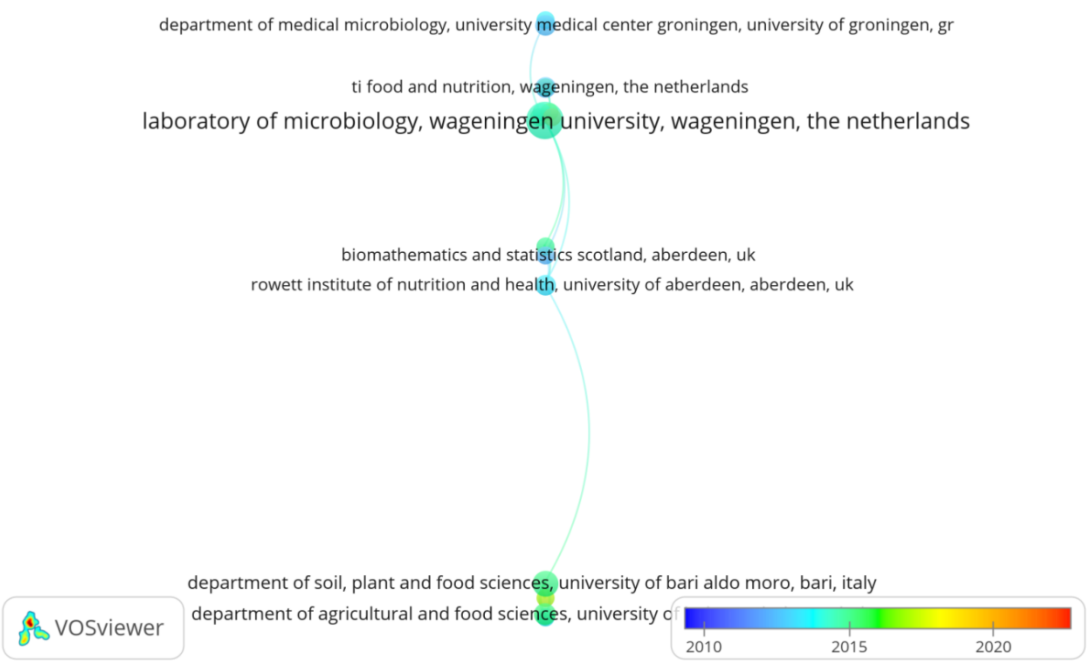

S Figure 15: Institutional collaborations for anthocyanin, gut microbiota and SCFA research (A) Network visualization (B) Overlay visualization (year wise)

A

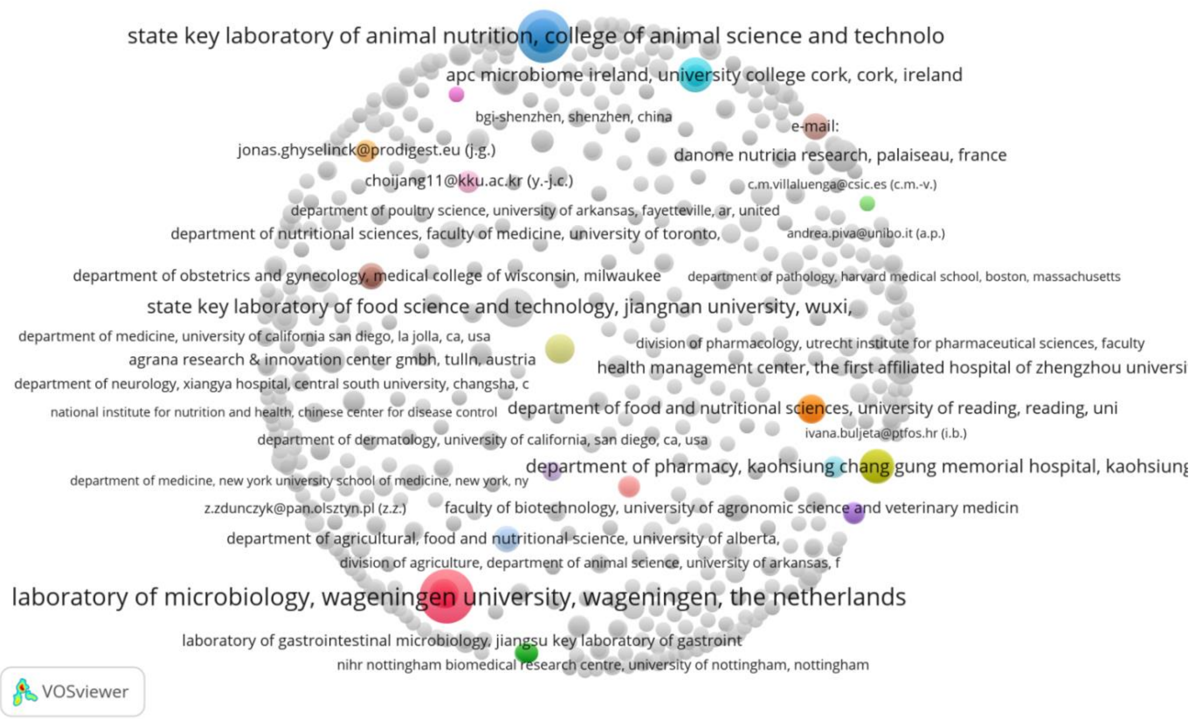

B

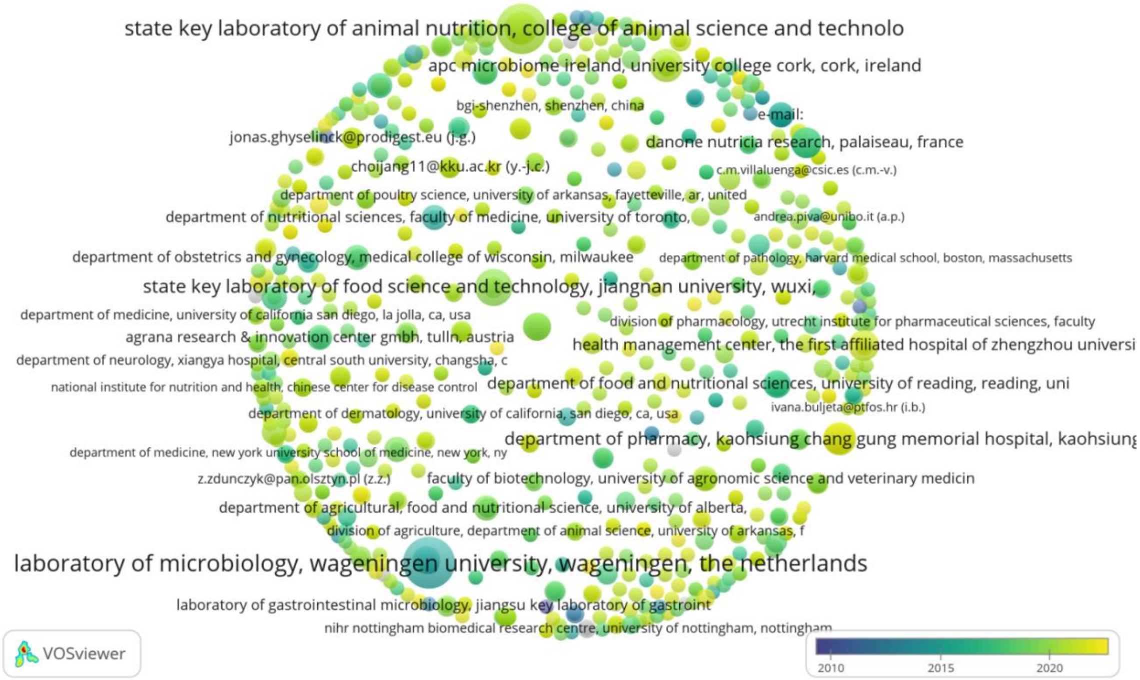

S Figure 16: Independent institutional collaborations for anthocyanin, gut microbiota and SCFA research (A) Network visualization **(B)** Overlay visualization (year wise)
